# Influence of Single Nucleotide Polymorphisms of ELOVL on Biomarkers of Metabolic Alterations in the Mexican Population

**DOI:** 10.3390/nu12113389

**Published:** 2020-11-04

**Authors:** María Luisa Maycotte-Cervantes, Adriana Aguilar-Galarza, Miriam Aracely Anaya-Loyola, Ma. de Lourdes Anzures-Cortes, Lorenza Haddad-Talancón, Akram Sharim Méndez-Rangel, Teresa García-Gasca, Víctor Manuel Rodríguez-García, Ulisses Moreno-Celis

**Affiliations:** 1 Facultad de Ciencias Naturales, Universidad Autónoma de Querétaro, Juriquilla, Querétaro CP 76230, Mexico; maru.maycotte@gmail.com (M.L.M.-C.); adrianaag.ga@gmail.com (A.A.-G.); aracely.anaya@uaq.mx (M.A.A.-L.); tggasca@uaq.edu.mx (T.G.-G.); 2 Código 46 SA de CV, Cuernavaca, Morelos 62498, Mexico; lourdes@codigo46.com.mx (M.d.L.A.-C.); lorenza@codigo46.com.mx (L.H.-T.); akramsharim@gmail.com (A.S.M.-R.); 3 Tecnologico de Monterrey, Escuela de Ingeniería y Ciencias, San Pablo, Querétaro 76130, Mexico

**Keywords:** biomarkers, ELOVL, metabolic alterations, Mexican population, Single Nucleotide Polymorphisms

## Abstract

The elongation of very long chain fatty acids (ELOVL) is a family of seven enzymes that have specific functions in the synthesis of fatty acids. Some have been shown to be related to insulin secretion (ELOVL2), and in the lipid profile (ELOVL6) and patients with various pathologies. The present work focused on the study of ELOVL polymorphs with clinical markers of non-communicable chronic diseases in the Mexican population. A sample of 1075 participants was obtained, who underwent clinical, biochemical, and nutritional evaluation, and a genetic evaluation of 91 genetic variants of ELOVL was considered (2–7). The results indicate a 33.16% prevalence of obesity by body mass index, 13.84% prevalence of insulin resistance by homeostatic model assessment (HOMA) index, 7.85% prevalence of high cholesterol, and 20.37% prevalence of hypercholesterolemia. The deprived alleles showed that there is no association between them and clinical disease risk markers, and the notable finding of the association studies is that the ELOVL2 variants are exclusive in men and ELVOL7 in women. There is also a strong association of ELOVL6 with various markers. The present study shows, for the first time, the association between the different ELOVLs and clinical markers of chronic non-communicable diseases.

## 1. Introduction

The elongases of very long chain fatty acid (ELOVL) family of fatty acid elongases includes seven enzymes that catalyze elongation of the carbon chain of fatty acids (FAs) during their condensation phase. Other studies have reported that ELOVLs are specific to the type of FA that elongate; ELOVL1, ELOVL3, ELOVL6, and ELOVL7 preferably elongate saturated fatty acids (SFAs) and monounsaturated fatty acids (MFAs); whereas ELOVL2 and ELOVL5 are specific to polyunsaturated fatty acids (PUFAs), and ELOVL4 elongates PUFAs and SFAs of very long chains [1,2]. Given the important role of lipids in the metabolism, their associations with the development of various pathological processes, such as hepatic steatosis, obesity, insulin resistance and diabetes mellitus 2, and cancer, are currently being studied [2]. As an example, the importance of ELOVL2 in pancreatic insulin secretion has been demonstrated in animal models [3]. An ELOVL3 knockout model has also been shown to be related to adiponectin reduction, adipose tissue expansion, and diet-induced resistance to obesity, in addition to a reduction in liver lipids and a decrease in triglycerides (TGs) in blood [4]. By comparison, the determination of the metabolic function of ELOVL4 has shown some influence on ceramides and very long chain fatty acids (VLFAs), mainly in the skin, brain, and eyes of mice [5,6]. Furthermore, possible participation in the pathophysiology of some diseases has not yet been evaluated. Moreover, ELOVL5 has been identified as an intermediate in the signaling for the export of glucose receptors to the membrane and the increase in the activity that promotes insulin resistance, mainly suppressing the activity of the transcriptional factor mTorc1. Studies have also reported that ELOVL5 regulates the levels of triglycerides in the liver [7]. The activity of ELOVL6 is the best studied, in addition to its importance in adipogenesis as a target of the transcription factor SRBP1 [8] and its effect in preventing insulin resistance when it is knocked down or knocked out in transgenic mice [9]. ELOVL7 is the least studied of all fatty acid elongases, but it has been associated with pathologies such as cancer and Parkinson’s [10].

Polymorphisms in the ELOVL enzymes affect the fatty acid composition of breast milk [11]. A study of a Costa Rican population found no association between the Single Nucleotide Polymorphisms (SNPs) of ELOVL2 (rs2295601, rs10498676, and rs3734397), ELOVL4 (rs17239120), and ELOVL5 (rs17544464, rs2115564, rs2294867, and rs761179) and the risk of acute myocardial infarction, but found an association with the metabolism of lipids [12]. Thus, the present work is designed to determine the relationship between 91 SNPs of ELOVL found in a Mexican population and the biomarkers of chronic non-communicable diseases. 

## 2. Materials and Methods 

A total of 1075 Mexican subjects participating in the SUSALUD-UAQ (University Health Program from the Autonomous University of Queretaro) program were sampled, comprising 563 women (52.3%) and 512 men (47.6%) of 18 to 30 years old. Subjects with previously diagnosed health problems were not included for analysis and those who did not have complete clinical, biochemical, and genetic information were excluded from the study. This study was approved by the Bioethics Committee (52FCN2017) of the Natural Science Department of the Autonomous University of Queretaro under the guidelines of the Declaration of Helsinki [13].

### 2.1. Evaluation of Nutritional Status and Body Composition

Anthropometric data was collected, including weight, height, and waist and hip circumferences, following the standard procedures of the World Health Organization, 2006 [14]. Height measurement was performed with a wireless transmission stadiometer (Brand SECA, Model 264; Hamburg, Germany). Waist circumference was measured by placing a tape measure on a line that is at the midpoint between the upper iliac crest and the border inferior costal, at the end of a normal expiration. Weight and body composition were determined using a multifrequency bioelectrical impedance device (SECA, model mBCA 515; Hamburg, Germany). Medical staff determined blood pressure of patients subject to the following conditions: without physical exercise and after resting for 10 to 15 min before measurement, back and arm supported, legs not crossed. The reference values of the Treatment of High Blood Cholesterol in Adults (ATPIII) 2005 revision (130/85 mmHg) were considered.

### 2.2. Evaluation of Biochemical Markers

Blood samples were collected from each subject to obtain plasma (BD Vacutainer Plus) and serum (BD Vacutainer SST II). Following collection of the blood samples, a complete blood count was performed in a Cell-dyn 1400 device (Abbot Mark, IL, USA). Plasma and serum were obtained by centrifugation of the whole blood sample at 2500 rpm for 10 min, and then aliquoted in 1.5 mL cryovials for subsequent analyses and stored at −70 °C (REVCO, Thermo Scientific, Waltham, MA, USA). The serum was used to analyze the concentrations of glucose, total cholesterol, triglycerides, and high-density lipoprotein (HDL), whereas the concentration of low-density lipoprotein (LDL) was calculated using the formula of Fridelwald (i.e., LDL = CT- (TG/5) + HDL)) in patients with TG < 400 mg/dL [15]. For those samples with TG > 400 mg/dL, the determination was made using colorimetric analysis. All biochemical determinations were carried out in duplicate in an automated Mindray BS 120 device (Medical International Limited, Shenzhen, China) using colorimetric enzymatic methods (SPINREACT S.A./S.A.U, Girona, Spain). Fasting glucose concentrations of less than 100 md/dL were considered normal [16]. 

The reference values from the National High Blood Pressure Education Program Working Group on High Blood Pressure in Children and Adolescents, 2004, were used for diagnostics of the lipid profile [17].

The plasma was used to analyze insulin levels. The insulin levels were analyzed according to the manufacturer’s instructions using an ELISA kit (Insulin ELISA 80-INSHU-E01.1, ALPCO INMUNOASSAYS). The readings were compiled using a Multiskan ascent spectrophotometer (Thermo, electron corporation) at a wavelength of 450 nm.

The homeostatic model assessment (*HOMA*) index was calculated according to the following equation:

(1) HOMA (IR)=[Insulin(μU)/mL) × Glucose(mmol/L)]/22.5

The cut-off values for insulin and *HOMA* index were based on those suggested by Munguía-Romero et al. (2013) [18].

### 2.3. DNA Purification and Genotyping

Genomic DNA was extracted using the Agencourt DNAdvance kit according to the manufacturer’s instructions (Beckmann Coulter, CA, USA). The extracted DNA was quantified and analyzed for its quality using the Spectrophotometer 190 (Molecular Devices, CA, USA) device. As quality criteria, a concentration higher than 40 ng/µl and an absorbance ratio 260/280 of 1.8–2 were used. The integrity of the DNA was also taken into account by electrophoretic running. Next, the Infinium HTS Automated Protocol (Illumina) [19] was followed with the objective of genotyping the samples. For this, the beadchip Global Screening Array-24 + V1.0/HTS CODIGO46_2017_01 was used. Genotypes described in Table 1 were determined using Genome Studio software. 

### 2.4. Genetic and Statistical Analyses

Allelic and genotypic frequencies were calculated using GenAlEx. Null alleles were removed from the dataset for further analysis and the remaining markers were tested in reference to the Hardy–Weinberg equilibrium (HWE). Private alleles were also identified and quantified for the analyzed data. SNPs were examined for associations with the studied biomarkers, therefore means and their standard deviations were analyzed with Student’s t-tests. We also tested the statistical homogeneity of the effects on body mass index (BMI) in the corresponding regression model between clinical biomarkers. The strength of association between variables was measured by calculating the odds ratio (OR) and 95% confidence intervals using logistic regressions performed with SPSS (ver. 9.6) [20] statistical software; the regression coefficients were tested for significance and the *p*-value used to reject the null hypothesis was 0.05. The multivariate logistic regression model used to calculate the risk associations was controlled for potential confounders such as sex and age. The recessive genotypes were compared to both dominant and heterozygous genotypes, and tested for statistical significance (*p*-value = 0.05).

## 3. Results

### 3.1. Characteristics of the Subjects

From the original sample of 1075, 476 participants were eliminated due to having incomplete data for the study, so a final sample of 599 subjects was used. Table 2 shows the general characteristics of the population, in which it can be seen that 311 of the subjects (51.9%) were women and 288 (48.1%) were men, with an average age of 19.1 ± 1.9 years. A comparison of means was carried out in both populations to establish that there were no significant differences in the variables independent of the sex. Significant statistical differences occurred in variables typically classified for both of the sexes.

### 3.2. Nutritional Alterations of Subjects

**Figure 1:**
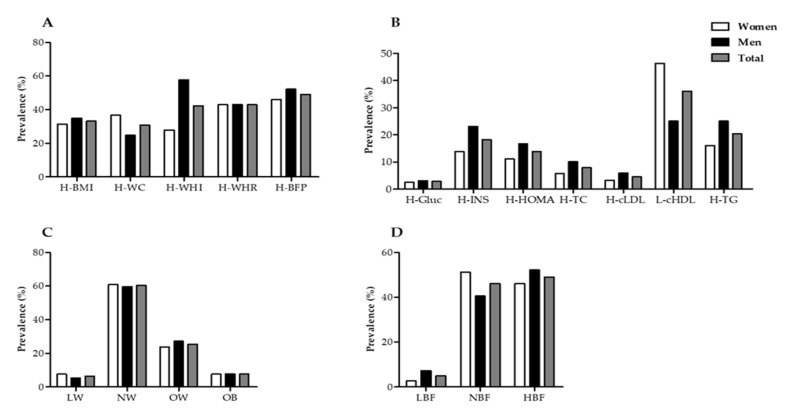
Distribution of nutritional status (**A**) Prevalence of anthropometric and body composition alterations. H-BMI: body mass index > 25.0 kg/m^2^; H-WC: waist circumference (women > 0.80 cm and men > 90 cm); H-WHI: waist–hip index (women > 0.85 cm and men > 95 cm); H-WHR: waist–height ratio > 0.50; H-BFP (women > 25% and men > 20%). (**B**) Prevalence of biochemical alterations. H-Gluc: high glucose; >100 mg/dL; H-INS: high insulin, >14 µU/mL for women and >11 µU/mL for men; H-HOMA: high HOMA index: >2.9 for women and >2.3 for men; H-TC: high total cholesterol, >200 mg/dL; H-cLDL: elevated low-density lipoproteins, >130 mg/dL. L-cHDL: low high-density lipoproteins, ≤50 mg/dL for women and ≤40 mg/dL for men; H-TG: high triglycerides > 150 mg/dL. (**C**) Nutritional status according to BMI. LW: low weight: BMI ≤ 18.5 kg/m^2^; NW: Standard weight: BMI 18.5–24.9 kg/m^2^; OW: Overweight: BMI 25–29.9 kg/m^2^; OB: obesity: BMI ≥ 30 kg/m^2^. (**D**) Nutritional status according to the percentage of body fat for men: LBF: low body fat < 8%; NBF: body fat 8.1–20%; HBF: high body fat 20.1–25%; for women: low body fat < 15%; NBF: body fat 15–35%; HBF: high body fat > 35% [21].

Metabolic alterations according to anthropometric and body composition variables (Figure 1A) indicate the evaluated population has a prevalence of overweight and obesity according to the BMI of 33.16%, which was 31.46% for women and 34.98% for men. Similarly, with respect to the High Waist–Hip Index (H-WHI), 42.35% of the population was above the recommended level, and was significantly higher in men (57.84%) than in women (27.92. Furthermore, the alteration of body composition with the highest prevalence in all of the population (n = 599) was due to the percentage of high body fat, which was 49.04% in the total population (46.10% in women and 52.17% in men). 

Significant alterations were also found in biochemical variables (Figure 1B). According to glucose (H-Gluc) values, the prevalence of high levels was low (2.84%), and was 2.57% for women and 3.12% for men. High insulin (H-INS) levels prevailed in 18.2% of the population. It is worth noting that men had higher prevalence (23.12%) than women (13.84%) in this marker. This was similar to the case of the high HOMA index (H-HOMA), which was found be more prevalent in men (16.67%) than in women (11.15%). Regarding lipid metabolism markers, high total cholesterol (H-TC) had a prevalence of 7.85%, and was higher in men (10.07%) than in women (5.79%). Similarly, the high levels of low-density cholesterol (H-cLDL) had a prevalence of 4.51%, with values of 3.33% for women and 5.90% for men. The opposite case was observed in low levels of high-density cholesterol (L-cHDL), the overall prevalence of which was observed to 36.0%, and was higher in women (46.30%) than in men (25.0%). The prevalence of high triglycerides (H-TG) was observed to be 20.37%, and was higher in men (25.0%) than in women (16.08%).

Figure 1C shows the nutritional status of the study subjects according to their BMI, in which values of 6.46% for low weight (LW) were obtained, 7.62% for women and 5.30% for men; 60.34% for normal weight (NW), 59.72% for women and 60.93% for men; and for overweight (OW) the total prevalence was 25.47%, and slightly higher in men (27.21%) than in women (23.84%). For obesity (OB), the prevalence of the population was 7.69%, with 7.62% for women and 7.77% for men. Nutritional status was determined according to the percentage of body fat (Figure 1D), in which a prevalence of low body fat of 4.9% was observed, with values of 2.71% for women and 7.75% for men. By comparison, the total prevalence for normal fat percentage was 46.09%, with values of 51.19% for women and 40.58% for men. Regarding the percentage of high fat, a prevalence of 49.04% was found in the total population, with a higher proportion of men (52.17%) than women (46.10%) presenting this condition.

### 3.3. Allelic Frequencies, HWE Analysis, and Private Alleles

From the 91 analyzed SNP polymorphisms of ELOVL, only 77 were used for further analysis after null alleles were excluded from the dataset. The allelic and genotypic frequencies analyzed in the sample (n = 599) are shown in Table S1 in the Supplementary Material. ELOVL2 and ELOVL6 were highly diverse with a total of 15 analyzed markers found for the former and 37 for the latter. Most markers showed a significant deviation from the HWE (*p* > 0.05) after sequential Bonferroni correction. 

Private alleles were identified after the population was analyzed independently by males and females, and once separated, both subpopulations were analyzed against all biomarkers. This was because some biomarkers behave differentially among sexes. Most of the private alleles were found to have a positive input on the biomarkers (Table 3), with the exception of two SNPs from ELOVL5, rs72938776 (allele A, freq. 0.019) and rs72940713 (allele G, freq. 0.019), which were significantly (*p* > 0.05) associated with the waist–height ratio (WHR) and percentage of body fat (%BF). 

### 3.4. Association of SNPs with Clinical Markers of Risk to Chronic Non-Communicable Diseases

To facilitate the analysis of the results, only those whose associations were statistically significant are presented in the following tables and all of the results of the association analyses and their respective *p*-values are shown in the Supplementary Material (S2). For the general population, the associations resulted in five variants associated with risk for ELOVL2, one for ELOVL4, two for ELOVL5, nine for ELOVL6, and three for ELOVL7, giving a total of 20 variants associated with risk markers (Table 4). By comparison, according to the results, 12 genetic variants were found to be protective factors for the risk markers for chronic non-communicable diseases, of which three are for ELOVL2, one for ELOVL3, one for ELOVL5, and seven for ELOVL6 (Table 5). The highest levels of risk identified by the analysis for the total population were found in ELOVL5 and ELOVL6.

When the population was divided by sex for posterior analyses, the results showed 15 SNPs of ELOVL for women were related to clinical markers of chronic non-communicable diseases as risk factors (two for ELOVL5, eight for ELOVL6, and five for ELOVL7) in which the variant rs72938776 of ELOVL5 obtained an OR of 11.37 for high LDL, whereas the rs9370194 variant of the same ELOVL was found with an OR of 2.92 for high total cholesterol. Regarding ELOVL6, the variants rs59634436 (OR = 2.761), rs10033691 (OR = 2.102), rs2005701 (OR = 1.717), and rs11937052 (OR = 2.0) were associated with high BMI; similarly, rs2005701 (OR = 2.163) was associated with elevated waist circumference. Regarding the association with biochemical markers, it was observed that rs76145164 was associated with elevated triglycerides (OR = 5.667) and rs72679246 with high glucose (OR = 19.2), and high total cholesterol had an association with rs17041272 (OR = 3.1), whereas rs10033691 (OR = 4.479) and rs78160528 (OR = 3.605) were associated with high LDL. The SNP of ELOVL7 rs1563517 was associated with elevated waist circumference (OR = 1.716) and high waist–height index (OR = 1.806) and rs4700398 showed associations with high BMI (OR 1.964). Regarding biochemical markers, rs1563517 was also associated with elevated insulin (OR = 3.126) and rs76641655 with high triglycerides (OR = 4.452), whereas rs115159664 was associated with high total cholesterol (OR = 7.125) and high LDL (OR = 8.111), and rs12188996 was associated with high LDL with an OR of 10.963 (Table 6).

Interestingly, in women, protective factors were found only in ELOVL2 and ELOVL6 variants. For ELOVL2, rs2281591 is associated with protection for a high waist circumference (OR = 0.610). The two SNPs of ELOVL6 are rs6533491, which is a protective factor for high waist circumference (OR = 0.526) and high waist–height radius (OR = 0.559); and rs11098065, which shows protection against low HDL (OR = 0.582) (Table 7).

In men, the results indicate that 15 SNPs are associated with clinical markers of risk for chronic non-communicable diseases (five for ELOVL2, one for ELOVL4, two for ELOVL5, and seven for ELOVL6). Regarding ELOVL2, the results surprisingly showed that the five SNPs with significant risk associations—rs8523, rs3734398, rs2236212, rs3798713, and rs4532436—are related to high levels of insulin and cholesterol, in addition to a high HOMA index, with an OR ranging from 2.080 to 3.154, thus showing an important relationship. Similarly, for ELOVL4 it was found that rs12196014 is associated with high triglycerides (OR = 1.978). For ELOVL5, rs41273878 and rs114271869 were associated with high triglycerides (OR = 4.176) and low HDL (OR = 5.274), respectively. Regarding the SNPs of ELOVL6, it was observed that rs114422025 is associated with a high HOMA index (OR = 4.929); rs59634436 is associated with a high percentage of body fat (OR = 2.333), with elevated levels of insulin (OR = 2.87) and blood glucose (OR = 7.407), and with a high HOMA index (OR = 3.635). In addition, rs10033691 was found to have associations with ORs for the same markers of 3.299, 2.824, 5.765, and 2.957, respectively. rs78160528 is associated with low HDL (OR = 5.299) and high LDL (OR = 5.889). rs11937052 has associations with elevated waist circumference (OR = 2.441), elevated body fat percentage (OR = 2.101), elevated glucose (OR = 5.577), and low HDL levels (OR = 2.105). rs9997926 was found to be a risk factor for elevated insulin (OR = 5.714), high BMI (OR = 2.068), and elevated waist circumference (OR = 2.131). Finally, for ELOVL6, rs17041272 risk was associated with elevated body fat percentage with an OR = 1.933 (Table 8).

The consistency in the results of ELOVL2 is surprising, because in the risk factors (Table 8) in the associations that indicate protection, the six variants that were found to have significant associations—rs17606561, rs2281591, rs9295757, rs3798721, rs3798722, and rs9393903—are only associated with elevated insulin levels and an elevated HOMA index, with an OR ranging from 0.204 to 0.422. We also found protective associations in ELOVL3, for which rs10748816 was associated with the radius waist height (OR = 5.08); and in ELOVL5, for which rs9370194 was associated with a high percentage of fat (OR = 0.537). By comparison, the results of protective associations of ELOVL6 show that rs11098065 is associated with high levels of insulin (OR = 0.436), as is rs6815102 (OR = 0.478); rs766216 also shows protective associations with elevated total cholesterol (OR = 0.34) and elevated LDL levels (OR = 0.177). rs4326075 is associated with H-LDL (OR = 0.552), whereas rs59111930 was found to be associated with protection against high triglyceride levels (OR = 0.5) and low HDL levels (OR = 0.582). Finally, rs6824447 was found to be a protective factor for high BMI (OR = 0.516), high waist circumference (OR = 0.551), high total cholesterol (OR = 0.268), and high LDL levels (OR = 0.227) (Table 9). It is interesting to note that ELOVL2 SNPs are present in the male population whereas ELOVL7 SNPs are only present in women.

## 4. Discussion

The current work presents, for the first time, a global study in human populations of the association between genetic variants of ELOVL and clinical markers of chronic non-communicable diseases.

Mexico ranks first in the world in childhood obesity and second in adult obesity, according to data from the National Health and Nutrition Survey (ENSANUT, for its acronym in Spanish) 2016. The combined prevalence of overweight and obesity in adolescents between 12 and 19 years was 36.3%, which is 1.4 percentage points higher than the prevalence in 2012 (34.9%). In adults over 20 years of age, the combined prevalence of overweight and obesity was 71.2% in 2012 and 72.5% in 2016, representing a non-significant difference of 1.3 percentage points [22,23]. This shows a clear trend of increase in the prevalence of overweight and obesity. Furthermore, this trend is reflected in our results, which show a 33% prevalence of overweight and obesity in the population with an average age of 19 years, but a marked difference when the percentage of body fat is analyzed, which shows a prevalence of high fat of 49% in the total population evaluated.

Similar studies in the Mexican population have reported the cumulative prevalence of overweight and obesity as 32% [24,25]. With respect to the WHR, 42.35% of the population was found to be above the recommended level, with this ratio considerably higher in men than in women [26]. Similarly, the body composition alteration with the highest prevalence in the population was the elevated body fat percentage, with 49.04% of young people experiencing the alteration (Figure 1A). This may be worrying because one of the main causes of obesity comorbidities is lipotoxicity or ectopic fat accumulation [27,28,29]. According to Murguía-Romero (2013) [17], the prevalence of elevated insulin found in a sample of young Mexicans was 9.5% in women and 12.4% in men, and in the case of high HOMA-IR, it was found to be 12.1% in women and 15.3% in men. Our results suggest that the prevalence of obesity and insulin resistance have increased considerably [30]. The results obtained are similar to those reported in other studies, both nationally, 35% [31], and at the state level, 33% [32]. It is known that the BMI underestimates the real prevalence of obesity due to its low specificity in the diagnosis, so the nutritional status of the sample was evaluated according to the %BF.

As mentioned previously, few studies have reported certain correlations between the SNPs found in other human populations and the metabolism of PUFA. The current work found the presence of private alleles of ELOVL5, which could be directly related to the %BF and the risk of cardiovascular diseases in females. Although these and other alleles of the ELOVLs have a low frequency represented within the population, this finding might also reveal the evolutionary process that was undergone by these markers. The frequencies of these alleles within the sampled population, and their HWE deviations and negative health impacts, suggest a possible negative selection process of these makers within the studied population [33].

There was a difference in the associations of SNPs with clinical markers between men and women, shown by the difference in ELOVL2 associated with clinical markers only in men, and ELOVL7 only in women. Although there is no scientific evidence for genetic variants of ELOVL5 related to chronic non-communicable diseases, we observe that ELOVL5 increases the risk for elevated blood levels of LDL (rs72938776) and total cholesterol (rs9370194) by 11.0 and 2.9 times, respectively. For the same gene in men, the associations were for SNP rs41273878 with high triglycerides (OR = 4.1) and rs114271869 with low LDL (OR = 5.2). ELOVL5 in experimental mice has been shown to regulate the degradation of hepatic triglycerides, which is related to an increase in lipase levels in adipocytes [34]. Similarly, epigenetic association studies have shown promising results in the analysis of the gene regulation of ELOVL in relation to type 2 diabetes mellitus [35]. 

For ELOVL6 it was observed that, of the eight SNPs of ELOVL6, four are related to a high body mass index in women (rs59634436, rs10033691, rs2005701, and rs11937052). Four different SMPs are related to %BF in men (rs59634436, rs10033691, rs11937052, and rs17041272), of which only rs17041272 has been studied for its participation in insulin sensitivity in the Spanish population; results show this SNP to be at risk for insulin resistance. This provides a starting point for the specific investigation of this genetic variant [36]. Similarly, it was observed that mice deficient in ELOVL6 and fed a high fat diet developed obesity but not hyperinsulinemia, hyperglycemia, or insulin resistance [37]. Our findings suggest a difference in ELOVL6 activity in men compared to women, because although the association of SNPs in women was skewed more towards anthropometric markers than in men, an important correlation was observed between elevated insulin levels, and the index of elevated HOMA and elevated glucose.

Regarding the genetic variants of ELOVL7 only associated with risk markers in women, until now no scientific information has existed to support or rule out the role of this enzyme in the physiopathogenesis of any chronic non-communicable disease. The one exception is the relationship attributed to prostate cancer, in which it was observed that enzyme’s overexpression occurred in tumor cells. It was found that eliminating the enzyme significantly decreased the growth of these cells, leading the authors to propose a relationship with the androgenic pathway of ELOVL7 [38].

ELOVL2 is clearly one of the most-studied members of the ELOVL family due to its effects on different pathological processes. It has been observed that changes in the methylation of ELOVL2 are related to the aging process [39]. Similarly, protective effects have been attributed to ELOVL2 in pancreatic beta cells of both rodents and humans, through an increase in the oxidation of mitochondrial palmitate, thus avoiding cell death induced by glycolipoxidation [40]. In another study conducted in experimental animals, it was discovered that ELOVL2 is necessary for glucose-mediated insulin secretion, which depends on the endogenous production of docosahexaenoic acid in which ELOVL2 is actively involved [3]. In a study carried out in a population of Tunisia, in which two SNPs of desaturases (FAD1 and FAD2) and one of ELOVL2 (rs3756963) were analyzed, the authors observed that the minor allele C is related to low triglyceride levels and low body mass index, which confers a protective factor to the population with this variant [41]. Similarly, in another study carried out in Italian children and adolescents, a similar behavior was observed in the association of genetic variants of ELOVL2 with indicators of obesity and insulin resistance, in addition to alterations in the blood lipid profile [42]. Like the aforementioned authors, our results show an important association of ELOVL2 with markers of insulin resistance and elevated total cholesterol, which indicates the value of a larger study to specifically identify the role of this enzyme in the pathophysiology of chronic non-communicable diseases.

## 5. Conclusions

In the present study, a tendency to increase the prevalence of clinical markers of chronic non-communicable diseases in the Mexican population was observed. Although these markers are subject to an environmental influence, we also observed the genetic component and, in particular, the association of ELOVL2, ELOVL5, ELOVL6, and ELOVL7. In addition, results highlighted the differences in the associations between men and women, such as in the cases of ELOVL2 and ELOVL7. These findings indicate the value of a more in-depth study of these genetic variants and their metabolic and physiological functions.

## Figures and Tables

**Table 1:** Genetic markers analyzed.

Gene	Genetic Variant	Alleles	Functional Consequence
ELOVL2	rs8523	(A/G)	genic downstream transcript variant, 3′ UTR variant.
ELOVL2	rs3734396	(A/G)	genic downstream transcript variant, 3′ UTR variant.
ELOVL2	rs17606561	(A/G)	genic downstream transcript variant, 3′ UTR variant.
ELOVL2	rs3734398	(T/C)	genic downstream transcript variant, 3′ UTR variant.
ELOVL2	rs2281591	(A/G)	intron variant, genic downstream transcript variant.
ELOVL2	rs2236212	(G/C)	intron variant, genic downstream transcript variant.
ELOVL2	rs3798713	(G/C)	intron variant.
ELOVL2	rs7765206	(A/C)	intron variant, genic upstream transcript variant.
ELOVL2	rs116279801	(T/C)	intron variant, genic upstream transcript variant.
ELOVL2	rs9295757	(T/G)	intron variant, genic upstream transcript variant.
ELOVL2	rs3798721	(A/C)	intron variant, genic upstream transcript variant.
ELOVL2	rs16870899	(A/G)	intron variant, genic upstream transcript variant.
ELOVL2	rs3798722	(A/G)	intron variant, genic upstream transcript variant.
ELOVL2	rs9393903	(A/G)	intron variant, upstream transcript variant, genic upstream transcript variant.
ELOVL2	rs4532436	(G/C)	genic downstream transcript variant, 3′ UTR variant.
ELOVL2	rs12195587	(A/G)	downstream gene transcription variant, synonym variant, coding sequence variant.
ELOVL3	rs10748816	(A/G)	intron variant.
ELOVL3	rs36103207	(A/G)	nonsense variant, coding sequence variant.
ELOVL4	rs3812153	(T/C)	coding sequence variant, nonsense variant.
ELOVL4	6:80628844	(A/G)	
ELOVL4	rs117891930	(T/C)	intron variant.
ELOVL4	rs144198896	(A/G)	intron variant.
ELOVL4	rs80246554	(T/C)	intron variant.
ELOVL4	rs12196014	(A/G)	intron variant.
ELOVL4	rs9448863	(A/G)	intron variant.
ELOVL4	rs16891339	(A/G)	intron variant.
ELOVL5	rs41273878	(A/C)	coding sequence variant, synonym variant, downstream gene transcription variant.
ELOVL5	rs41273880	(T/C)	coding sequence variant, nonsense variant, synonym variant, downstream gene transcription variant.
ELOVL5	rs72938776	(A/G)	intron variant, genic downstream transcript variant.
ELOVL5	rs182937551	(T/C)	downstream gene transcription variant, intron variant
ELOVL5	rs36054518	(A/G)	intron variant.
ELOVL5	rs209487	(T/G)	intron variant.
ELOVL5	rs115397424	(A/G)	intron variant.
ELOVL5	rs13208390	(T/G)	intron variant.
ELOVL5	rs72940713	(T/C)	intron variant.
ELOVL5	rs114271869	(T/G)	intron variant.
ELOVL5	rs2073040	(A/G)	intron variant, genic downstream transcript variant.
ELOVL5	rs9370194	(C/T)	intron variant.
ELOVL6	rs11098065	(A/G)	intron variant.
ELOVL6	rs17041284	(T/C)	intron variant.
ELOVL6	rs7662161	(T/C)	intron variant.
ELOVL6	rs77958351	(A/G)	intron variant.
ELOVL6	rs77808755	(A/G)	intron variant.
ELOVL6	rs59634436	(A/C)	intron variant.
ELOVL6	rs78160528	(T/C)	intron variant.
ELOVL6	rs16997129	(T/C)	intron variant.
ELOVL6	rs3813827	(A/G)	intron variant.
ELOVL6	rs11737840	(T/C)	intron variant.
ELOVL6	rs10033691	(T/C)	intron variant.
ELOVL6	rs2005701	(T/C)	intron variant.
ELOVL6	rs76145164	(T/C)	intron variant.
ELOVL6	rs76338299	(T/G)	intron variant.
ELOVL6	rs6533491	(T/C)	intron variant.
ELOVL6	rs72679222	(A/G)	intron variant.
ELOVL6	rs11937052	(A/G)	intron variant.
ELOVL6	rs11098070	(A/G)	intron variant.
ELOVL6	rs80343897	(T/C)	intron variant.
ELOVL6	rs373773495	(T/C)	intron variant.
ELOVL6	rs114422025	(T/C)	intron variant.
ELOVL6	rs6533495	(A/G)	intron variant.
ELOVL6	rs28722886	(T/C)	intron variant.
ELOVL6	rs6533497	(T/C)	intron variant.
ELOVL6	rs77504516	(A/G)	intron variant.
ELOVL6	rs6815102	(T/C)	intron variant.
ELOVL6	rs4326075	(A/C)	intron variant.
ELOVL6	rs116418972	(A/G)	intron variant.
ELOVL6	rs11729740	(T/C)	intron variant.
ELOVL6	rs2035415	(T/C)	intron variant.
ELOVL6	rs17041402	(A/C)	intron variant.
ELOVL6	rs59111930	(A/G)	intron variant.
ELOVL6	rs74874270	(A/G)	intron variant.
ELOVL6	rs1384331	(T/G)	intron variant.
ELOVL6	rs72679246	(A/C)	intron variant.
ELOVL6	rs78563565	(T/C)	intron variant.
ELOVL6	rs6533498	(A/C)	intron variant.
ELOVL6	rs9997926	(C/T)	intron variant.
ELOVL6	rs6824447	(A/G)	upstream transcription variant.
ELOVL6	rs17041272	(C/G)	3′ UTR region variant.
ELOVL7	rs75621404	(A/G)	intron variant, genic downstream transcript variant.
ELOVL7	rs143990657	(A/G)	downstream gene transcription variant, intron variant.
ELOVL7	rs115862620	(T/C)	intron variant, genic downstream transcript variant.
ELOVL7	rs1563517	(T/G)	intron variant, genic downstream transcript variant.
ELOVL7	rs12188996	(A/C)	downstream gene transcription variant, intron variant.
ELOVL7	rs60258111	(T/C)	intron variant, genic downstream transcript variant.
ELOVL7	rs16878426	(T/C)	downstream gene transcription variant, intron variant.
ELOVL7	rs6872863	(A/G)	intron variant, genic downstream transcript variant.
ELOVL7	rs76641655	(T/G)	intron variant, genic downstream transcript variant, coding sequence variant, synonym variant.
ELOVL7	rs145299240	(T/C)	downstream gene transcription variant, intron variant, genic upstream transcript variant.
ELOVL7	rs114011218	(T/C)	upstream gene transcription variant, intron variant, genic downstream transcript variant.
ELOVL7	rs115159664	(T/C)	upstream gene transcription variant, intron variant, genic downstream transcript variant.
ELOVL7	rs4700398	(A/G)	upstream gene transcription variant, intron variant.

**Table 2:** General characteristics of the population.

Biomarkers	Total (N = 599)		Women (N = 311)		Men (N = 288)		
	Mean	S.D.	Mean	S.D.	Mean	S.D.	*p*-Value
Age (years)	19.18	1.95	19.07	1.80	19.29	2.09	0.162
Weight (kg)	65.07	13.70	59.88	12.06	70.65	13.18	0.000
BMI (kg/m^2^)	23.76	4.30	23.50	4.44	20.04	4.14	0.127
Waist circumference (cm)	80.98	11.83	78.24	11.62	83.92	11.36	0.000
Waist–Hip Ratio	0.83	0.07	0.81	0.07	0.86	0.06	0.000
Waist–height Ratio	0.49	0.07	0.49	0.07	0.49	0.07	0.970
Body Fat (%)	26.41	0.56	31.39	7.34	21.08	8.06	0.000
Glucose (mg/dL)	83.53	9.04	82.25	9.03	84.91	8.86	0.000
Insulin (µg/mL)	7.87	5.65	7.99	0.07	7.73	5.14	0.581
HOMA-IR Index	1.63	1.20	1.63	1.24	1.63	1.15	0.999
Triglycerides (mg/dL)	105.18	64.11	95.57	53.58	114.45	72.76	0.001
Total Cholesterol (mg/dL)	157.43	30.19	157.44	27.71	157.41	32.71	0.989
HDL (mg/dL)	50.72	12.55	53.25	13.33	48.02	11.07	0.000
LDL (mg/dL)	85.51	23.71	84.63	22.50	85.47	24.95	0.346

S.D.: Standard deviation; BMI: Body mass index; HDL: high-density lipoprotein; LDL: low-density lipoprotein. Student’s t-test of statistical significance, *p*-value < 0.05.

**Table 3:** Single Nucleotide Polymorphisms private alleles with significant associations with biomarkers.

Sex	Gene	Biomarker	Clinical Diagnosis	Locus	Allele	Frequency
Males	**ELOVL2**	%BF	Low body fat	rs116279801	A	0.025
		Glucose	Normal levels	rs7765206	A	0.028
		HOMA	No insulin resistance	rs7765206	A	0.031
	**ELOVL4**	WHR	Without cardiovascular risk	rs16891339	G	0.023
		LDL	Without cardiovascular risk	rs117891930	A	0.020
			Without cardiovascular risk	rs80246554	G	0.037
			Without cardiovascular risk	rs12196014	A	0.095
	**ELOVL5**	WHR	Without cardiovascular risk	rs36054518	G	0.021
		TG	Normal levels	rs36054518	G	0.021
	**ELOVL6**	Glucose	Normal levels	rs76145164	A	0.021
			Normal levels	rs76338299	A	0.053
		HOMA	No insulin resistance	rs77958351	A	0.021
		Cholesterol	Normal levels	rs76145164	A	0.021
			Normal levels	rs74874270	G	0.031
		LDL	Without cardiovascular risk	rs76145164	A	0.021
			Without cardiovascular risk	rs74874270	G	0.030
	**ELOVL7**	HOMA	No insulin resistance	rs115159664	G	0.024
		TG	Normal levels	rs115159664	G	0.023
Females	**ELOVL2**	Cholesterol	Normal levels	rs7765206	A	0.031
			Normal levels	rs16870899	G	0.022
		LDL	Without cardiovascular risk	587/ELOVL2	A	0.071
	**ELOVL5**	WHR	Without cardiovascular risk	rs72938776	A	0.019
		%BF	High levels	rs72938776	A	0.018
			High levels	rs72940713	G	0.019
	**ELOVL6**	Glucose	Normal levels	rs76145164	A	0.020
			Normal levels	rs74874270	G	0.029
		HOMA	No insulin resistance	rs17041402	C	0.023
		Cholesterol	Normal levels	rs17041402	C	0.022
			Normal levels	rs74874270	G	0.027
			Normal levels	rs78563565	G	0.024
		TG	Normal levels	rs17041402	C	0.025
		LDL	Without cardiovascular risk	rs11729740	A	0.062
			Without cardiovascular risk	rs17041402	C	0.022
			Without cardiovascular risk	rs74874270	G	0.027
			Without cardiovascular risk	rs78563565	G	0.023
	**ELOVL7**	WHI	Normal distribution	rs76641655	C	0.022

**Table 4:** Association between SNP of elongases of very long chain fatty acids (ELOVL) and clinical markers of chronic non-communicable diseases (risk factors).

Gene	SNP	Clinical Marker	OR	95% CI		*p*-Value
**ELOVL2**	rs8523	H-Insulin	2.048	1.238	3.388	0.005
		H-HOMA	1.847	1.132	3.013	0.013
		H-Cholesterol	2.628	1.400	4.935	0.002
	rs3734398	H-BMI	1.441	1.020	2.036	0.038
		H-Insulin	1.894	1.146	3.130	0.012
		H-HOMA	1.780	1.088	2.912	0.020
		H-Cholesterol	2.341	1.251	4.378	0.006
	rs2236212	H-Insulin	1.922	1.135	3.254	0.014
		H-HOMA	1.995	1.184	3.361	0.009
		H-Cholesterol	2.432	1.236	4.785	0.008
		H-LDL	2.856	1.136	7.181	0.020
	rs3798713	H-HOMA	1.765	1.058	2.944	0.028
		H-Cholesterol	2.485	1.263	4.888	0.007
		H-LDL	2.915	1.159	7.330	0.018
	rs4532436	H-Insulin	2.248	1.357	3.722	0.001
		H-HOMA	2.025	1.237	3.315	0.004
		H-Cholesterol	2.654	1.418	4.966	0.002
**ELOVL4**	rs80246554	H-HOMA	2.622	1.164	5.908	0.016
**ELOVL5**	rs72938776	H-BMI	12.447	1.488	104.127	0.003
		H-Insulin	6.973	1.145	42.469	0.015
	rs72940713	H-BMI	7.32	1.506	35.579	0.004
		H-%BF	8.561	1.064	68.899	0.016
		H-Insulin	6.953	1.142	42.346	0.015
**ELOVL6**	rs59634436	H-BMI	2.129	1.256	3.608	0.004
		H-%BF	1.879	1.091	3.238	0.021
		H-Insulin	2.237	1.168	4.284	0.013
		H-Glucose	3.597	1.225	10.561	0.013
		H-HOMA	2.023	1.052	3.888	0.032
	rs78160528	H-LDL	4.642	1.258	17.12	0.012
	rs10033691	H-BMI	1.844	1.14	2.983	0.012
		H-%BF	2.27	1.371	3.759	0.001
		H-Insulin	2.094	1.132	3.874	0.017
		H-Glucose	3.687	1.325	10.265	0.008
	rs76145164	H-Triglycerides	3.073	1.264	7.471	0.009
	rs11937052	H-BMI	1.797	1.113	2.901	0.015
		H-Waist	1.776	1.104	2.858	0.017
	rs114422025	H-HOMA	4.678	1.445	15.141	0.005
	rs72679246	H-Glucose	6.144	1.263	29.902	0.011
	rs9997926	H-WHR	1.975	1.098	3.553	0.021
	rs17041272	H-BMI	1.569	1	2.46	0.049
		H-%BF	1.794	1.133	2.839	0.012
		H-Insulin	2.147	1.198	3.847	0.009
		H-HOMA	1.956	1.096	3.491	0.021
**ELOVL7**	rs1563517	H- Waist	1.517	1.049	2.194	0.027
		H-Insulin	1.844	1.107	3.074	0.018
	rs115159664	H-LDL	3.847	1.06	13.959	0.028
	rs4700398	H-BMI	1.57	1.109	2.221	0.011
		H-WHI	1.438	1.036	1.996	0.03
		H-%BF	1.473	1.058	2.051	0.022

H-BMI: high body mass index > 25.0 kg/m^2^; H-Waist: high waist circumference (women > 0.80 cm and men > 90 cm); H-WHI: high waist–hip Index (women > 0.85 cm and men > 95 cm); H-WHR: high waist–height ratio > 0.50; H-%BF: high body fat percent (women > 35% and men > 20%). H-Glucose: high glucose; >100 mg/dL; H-INS: high insulin (>14 µU/mL for women and >11µU/mL for men); H-HOMA: high HOMA index (>2.9 for women and >2.3 for men); H-Cholesterol: high total cholesterol (>200 mg/dL); H-LDL: elevated low-density lipoproteins (>130 mg/dL); L-HDL: low high-density lipoproteins (≤50 mg/dL for women and ≤40 mg/dL for men); H-Triglycerides: high triglycerides (>150 mg/dL). The statistical analysis applied to this dataset was a multinominal regression (*p* ≤ 0.05).

**Table 5:** Association between SNP of ELOVL and clinical markers of chronic non-communicable diseases (protective factors).

Gene	SNP	Clinical Marker	OR	95% CI		*p*-Value
**ELOVL2**	rs17606561	H-HOMA	0.521	0.298	0.914	0.021
		H-Triglycerides	0.625	0.404	0.968	0.034
	rs2281591	H-Waist	0.641	0.445	0.924	0.017
		H-Insulin	0.517	0.297	0.899	0.018
		H-HOMA	0.479	0.276	0.831	0.008
	rs9393903	H-HOMA	0.560	0.322	0.972	0.038
**ELOVL3**	rs10748816	H-WHR	0.578	0.401	0.832	0.003
**ELOVL5**	rs2073040	L-HDL	0.686	0.475	0.992	0.045
**ELOVL6**	rs11098065	H-Insulin	0.499	0.289	0.861	0.011
		L-HDL	0.686	0.487	0.966	0.031
	rs7662161	H-LDL	0.331	0.123	0.89	0.022
	rs6533491	H-Waist	0.676	0.476	0.958	0.028
		H-WHR	0.704	0.508	0.977	0.035
	rs80343897	H-Glucose	0.202	0.045	0.895	0.02
		L-HDL	0.654	0.462	0.924	0.016
	rs6815102	H-Insulin	0.575	0.349	0.949	0.029
	rs4326075	L-HDL	0.552	0.312	0.977	0.042
	rs6824447	H-Cholesterol	0.447	0.242	0.825	0.008
		H-LDL	0.435	0.196	0.968	0.036

H-BMI: high body mass index > 25.0 kg/m^2^; H-Waist: high waist circumference (women > 0.80 cm and men > 90 cm); H-WHI: high waist–hip Index (women > 0.85 cm and men > 95 cm); H-WHR: high waist–height ratio > 0.50; H-%BF: high body fat percent (women > 35% and men > 20%). H-Glucose: high glucose; >100 mg/dL; H-INS: high insulin (>14 µU/mL for women and >11µU/mL for men); H-HOMA: high HOMA index (>2.9 for women and >2.3 for men); H-Cholesterol: high total cholesterol (>200 mg/dL); H-LDL: elevated low-density lipoproteins (>130 mg/dL); L-HDL: low high-density lipoproteins (≤50 mg/dL for women and ≤40 mg/dL for men); H-Triglycerides: high triglycerides (>150 mg/dL). The statistical analysis applied to this dataset was a multinominal regression (*p* ≤ 0.05).

**Table 6:** Association of SNPs with clinical markers of chronic non-communicable diseases in women (risk factors).

Gene	SNP	Clinical Marker	OR	95% CI		*p*-Value
**ELOVL5**	rs72938776	H-LDL	11.037	1.044	116.696	0.013
	rs9370194	H-Cholesterol	2.926	1.085	7.892	0.027
**ELOVL6**	rs59634436	H-BMI	2.761	1.314	5.802	0.006
	rs10033691	H-BMI	2.102	1.076	4.106	0.027
		H-LDL	4.479	1.21	16.584	0.015
	rs78160528	H-LDL	3.605	0.412	31.569	0.218
	rs2005701	H-BMI	1.717	1.004	2.937	0.047
		H-Waist	2.163	1.287	3.634	0.003
	rs76145164	H-Triglycerides	5.667	1.576	20.37	0.003
	rs11937052	H-BMI	2	1.03	3.884	0.038
	rs72679246	H-Glucose	19.2	3.085	119.485	0.001
	rs17041272	H-Cholesterol	3.1	1.101	8.73	0.025
**ELOVL7**	rs1563517	H-Waist	1.716	1.051	2.801	0.03
		H-WHR	1.806	1.075	3.033	0.025
		H-Insulin	3.126	1.441	6.778	0.003
	rs12188996	H-LDL	10.963	1.037	115.915	0.013
	rs76641655	H-Triglycerides	4.452	1.152	17.204	0.019
	rs115159664	H-Cholesterol	7.125	1.714	29.621	0.002
		H-LDL	8.111	1.503	43.76	0.004
	rs4700398	H-BMI	1.964	1.196	3.225	0.007

H-BMI: high body mass index > 25.0 kg/m^2^; H-Waist: high waist circumference (women > 0.80 cm and men > 90 cm); H-WHI: high waist–hip Index (women > 0.85 cm and men > 95 cm); H-WHR: high waist–height ratio > 0.50; H-%BF: high body fat percent (women > 35% and men > 20%). H-Glucose: high glucose; >100 mg/dL; H-INS: high insulin (>14 µU/mL for women and >11µU/mL for men); H-HOMA: high HOMA index (>2.9 for women and >2.3 for men); H-Cholesterol: high total cholesterol (>200 mg/dL); H-LDL: elevated low-density lipoproteins (>130 mg/dL); L-HDL: low high-density lipoproteins (≤50 mg/dL for women and ≤40 mg/dL for men); H-Triglycerides: high triglycerides (>150 mg/dL). The statistical analysis applied to this dataset was a multinominal regression (*p* ≤ 0.05).

**Table 7:** Association of SNPs with clinical markers of chronic non-communicable diseases in women (protective factors).

Gene	SNP	Clinical Marker	OR	95% CI		*p*-Value
**ELOVL2**	rs2281591	H-Waist	0.610	0.377	0.988	0.044
**ELOVL6**	rs6533491	H-Waist	0.526	0.329	0.841	0.007
		H-WHR	0.559	0.338	0.925	0.023
	rs11098065	L-HDL	0.582	0.369	0.919	0.02

H-BMI: high body mass index > 25.0 kg/m^2^; H-Waist: high waist circumference (women > 0.80 cm and men > 90 cm); H-WHI: high waist–hip Index (women > 0.85 cm and men > 95 cm); H-WHR: high waist–height ratio > 0.50; H-%BF: high body fat percent (women > 35% and men > 20%). H-Glucose: high glucose; > 100 mg/dL; H-INS: high insulin (>14 µU/mL for women and >11µU/mL for men); H-HOMA: high HOMA index (>2.9 for women and >2.3 for men); H-Cholesterol: high total cholesterol (>200 mg/dL); H-LDL: elevated low-density lipoproteins (>130 mg/dL); L-HDL: low high-density lipoproteins (≤50 mg/dL for women and ≤40 mg/dL for men); H-Triglycerides: high triglycerides (>150 mg/dL). The statistical analysis applied to this dataset was a multinominal regression (*p* ≤ 0.05).

**Table 8:** Association of SNPs with clinical markers of chronic non-communicable diseases in men (risk factors).

Gene	SNP	Clinical Marker	OR	95% CI		*p*-Value
**ELOVL2**	rs8523	H-Insulin	2.305	1.172	4.532	0.014
		H-HOMA	2.099	1.094	4.027	0.024
		H-Cholesterol	3.154	1.408	7.061	0.004
	rs3734398	H-Insulin	2.222	1.133	4.360	0.019
		H-HOMA	2.080	1.079	4.009	0.027
		H-Cholesterol	3.010	1.320	6.864	0.006
	rs2236212	H-Insulin	2.198	1.088	4.441	0.026
		H-HOMA	2.415	1.200	4.859	0.012
		H-Cholesterol	2.842	1.173	6.886	0.016
	rs3798713	H-Cholesterol	2.885	1.191	6.990	0.015
	rs4532436	H-Insulin	2.924	1.476	5.792	0.002
		H-HOMA	2.710	1.395	5.267	0.003
		H-Cholesterol	2.935	1.311	6.568	0.007
**ELOVL4**	rs12196014	H-Triglycerides	1.978	1.011	3.871	0.044
**ELOVL5**	rs41273878	H-Triglycerides	4.176	0.912	19.127	0.047
	rs114271869	L-HDL	5.274	1.228	22.651	0.013
**ELOVL6**	rs114422025	H-HOMA	4.929	0.961	25.264	0.036
	rs59634436	H-%BF	2.333	1.027	5.299	0.039
		H-Insulin	2.87	1.212	6.797	0.013
		H-Glucose	7.407	1.876	29.251	0.001
		H-HOMA	3.635	1.567	8.433	0.002
	rs78160528	L-HDL	5.299	1.234	22.757	0.013
		H-LDL	5.889	1.095	31.683	0.02
	rs10033691	H-%BF	3.299	1.493	7.288	0.002
		H-Insulin	2.824	1.232	6.469	0.011
		H-Glucose	5.765	1.475	22.524	0.005
		H-HOMA	2.957	1.342	6.514	0.005
	rs11937052	H-Waist	2.441	1.206	4.94	0.011
		H-%BF	2.101	1.009	4.375	0.044
		H-Glucose	5.577	1.429	21.766	0.006
		L-HDL	2.105	1.035	4.28	0.037
	rs9997926	H-Insulin	5.714	2.035	16.048	0.001
		H-BMI	2.068	1.118	3.826	0.019
		H-Waist	2.131	1.116	4.069	0.02
	rs17041272	H-%BF	1.933	1.016	3.68	0.043

H-BMI: high body mass index > 25.0 kg/m^2^; H-Waist: high waist circumference (women > 0.80 cm and men > 90 cm); H-WHI: high waist–hip Index (women > 0.85 cm and men > 95 cm); H-WHR: high waist–height ratio > 0.50; H-%BF: high body fat percent (women > 35% and men > 20%). H-Glucose: high glucose; >100 mg/dL; H-INS: high insulin (>14 µU/mL for women and >11µU/mL for men); H-HOMA: high HOMA index (>2.9 for women and >2.3 for men); H-Cholesterol: high total cholesterol (>200 mg/dL); H-LDL: elevated low-density lipoproteins (>130 mg/dL); L-HDL: low high-density lipoproteins (≤50 mg/dL for women and ≤40 mg/dL for men); H-Triglycerides: high triglycerides (>150 mg/dL). The statistical analysis applied to this dataset was a multinominal regression (*p* ≤ 0.05).

**Table 9:** Association of SNPs with clinical markers of chronic non-communicable diseases in men (protective factors).

Gene	SNP	Clinical marker	OR	95% CI		*p*-Value
**ELOVL2**	rs17606561	H-Insulin	0.291	0.122	0.693	0.004
		H-HOMA	0.204	0.077	0.539	0.001
	rs2281591	H-Insulin	0.240	0.101	0.572	0.001
		H-HOMA	0.204	0.083	0.503	0.001
	rs9295757	H-HOMA	0.403	0.184	0.881	0.020
	rs3798721	H-HOMA	0.381	0.174	0.833	0.013
	rs3798722	H-Insulin	0.422	0.199	0.894	0.022
		H-HOMA	0.357	0.168	0.759	0.006
	rs9393903	H-Insulin	0.299	0.125	0.713	0.004
		H-HOMA	0.204	0.077	0.539	0.001
**ELOVL3**	rs10748816	H-WHR	0.508	0.298	0.866	0.012
**ELOVL5**	rs9370194	H-%BF	0.537	0.319	0.907	0.019
**ELOVL6**	rs11098065	H-Insulin	0.436	0.21	0.906	0.024
	rs7662161	H-Cholesterol	0.34	0.134	0.863	0.018
		H-LDL	0.177	0.04	0.789	0.011
	rs6815102	H-Insulin	0.478	0.244	0.939	0.03
	rs4326075	H-LDL	0.552	0.312	0.977	0.04
	rs59111930	H-Triglycerides	0.5	0.291	0.859	0.011
		L-HDL	0.582	0.34	0.995	0.047
	rs6824447	H-BFI	0.516	0.313	0.85	0.009
		H-Waist	0.551	0.319	0.952	0.031
		H-Cholesterol	0.268	0.114	0.628	0.001
		H-LDL	0.227	0.072	0.714	0.006

H-BMI: high body mass index > 25.0 kg/m^2^; H-Waist: high waist circumference (women > 0.80 cm and men > 90 cm); H-WHI: high waist–hip Index (women > 0.85 cm and men > 95 cm); H-WHR: high waist–height ratio > 0.50; H-%BF: high body fat percent (women > 35% and men > 20%). H-Glucose: high glucose; >100 mg/dL; H-INS: high insulin (>14 µU/mL for women and >11µU/mL for men); H-HOMA: high HOMA index (>2.9 for women and >2.3 for men); H-Cholesterol: high total cholesterol (>200 mg/dL); H-LDL: elevated low-density lipoproteins (>130 mg/dL); L-HDL: low high-density lipoproteins (≤50 mg/dL for women and ≤40 mg/dL for men); H-Triglycerides: high triglycerides (>150 mg/dL). The statistical analysis applied to this dataset was a multinominal regression (*p* ≤ 0.05).

## Supplementary Materials

The following are available online at https://www.mdpi.com/2072-6643/12/11/3389/s1, S1: Table S1: Allele and genotype frequencies for all SNP markers. S2: Table S2: Association of SNPs with clinical markers of chronic non-communicable diseases.
