# Supplementary material for: Influence of Single Nucleotide Polymorphisms of ELOVL on Biomarkers of Metabolic Alterations in the Mexican Population
Source: Nutrients. 2020 Nov 4;12(11):3389. doi: 10.3390/nu12113389 (PMC7694210; doi:10.3390/nu12113389)
Supplement: Supplementary file 1 [file nutrients-12-03389-s001.pdf]

**Table 1.** Allele and genotype frequencies for all SNP markers.

| Gene | SNP | Genotype | Genotype Frequency (%) | Allele | Allele Frequency (%) |
|---|---|---|---|---|---|
| ELOVL2 | rs8523 | AA | 56.54 | Allele A | 75.00 |
| | | AG | 36.91 | Allele G | 25.00 |
| | | GG | 6.54 | | |
| | rs3734396 | AA | 87.79 | Allele A | 93.48 |
| | | AG | 11.37 | Allele G | 6.52 |
| | | GG | 0.84 | | |
| | rs17606561 | GG | 63.93 | Allele G | 80.00 |
| | | GA | 32.21 | Allele A | 20.00 |
| | | AA | 3.86 | | |
| | rs3734398 | GG | 53.09 | Allele G | 72.50 |
| | | GA | 38.90 | Allele A | 27.50 |
| | | AA | 8.01 | | |
| | GSA-rs2281591 | AA | 60.34 | Allele A | 77.60 |
| | | AG | 34.62 | Allele G | 22.40 |
| | | GG | 5.04 | | |
| | rs2236212 | GG | 43.91 | Allele G | 66.10 |
| | | GC | 44.41 | Allele C | 33.90 |
| | | CC | 11.69 | | |
| | rs3798713 | GG | 44.39 | Allele G | 66.33 |
| | | GC | 43.89 | Allele C | 33.70 |
| | | CC | 11.73 | | |
| | GSA-rs7765206 | CC | 94.82 | Allele C | 97.25 |
| | | CA | 4.84 | Allele A | 2.80 |
| | | AA | 0.33 | | |
| | rs9295757 | CC | 63.09 | Allele C | 79.53 |
| | | CA | 32.89 | Allele A | 20.50 |
| | | AA | 4.03 | | |
| | rs3798721 | AA | 58.82 | Allele A | 77.00 |
| | | AC | 36.30 | Allele C | 23.00 |
| | | CC | 4.87 | | |
| | GSA-rs16870899 | AA | 95.66 | Allele A | 97.75 |
| | | AG | 4.17 | Allele G | 2.30 |
| | | GG | 0.17 | | |
| | rs3798722 | AA | 55.63 | Allele A | 74.70 |
| | | AG | 6.22 | Allele G | 25.29 |
| | | GG | 38.15 | | |
| | rs9393903 | GG | 63.59 | Allele G | 79.95 |
| | | GA | 32.72 | Allele A | 20.05 |
| | | AA | 3.69 | | |
| | rs4532436 | GG | 55.91 | Allele G | 74.58 |
| | | CG | 37.33 | Allele C | 25.42 |
| | | CC | 6.76 | | |

| | | | | | |
|---|---|---|---|---|---|
| | rs12195587 | GG | 86.15 | Allele G | 92.91 |
| | | AG | 13.51 | Allele A | 7.10 |
| | | AA | 0.34 | | |
| ELOVL3 | rs10748816 | AA | 23.79 | Allele G | 51.76 |
| | | GA | 48.91 | Allele A | 48.24 |
| | | GG | 27.30 | | |
| | GSA-rs36103207 | GG | 97.83 | Allele G | 98.92 |
| | | GA | 2.17 | Allele A | 1.09 |
| | | AA | - | | |
| ELOVL4 | rs3812153 | AA | 68.90 | Allele A | 82.78 |
| | | AG | 27.76 | Allele G | 17.22 |
| | | GG | 3.34 | | |
| | GSA-rs117891930 | GG | 96.33 | Allele G | 98.16 |
| | | GA | 3.67 | Allele A | 1.84 |
| | | AA | - | | |
| | GSA-rs80246554 | AA | 93.82 | Allele A | 96.83 |
| | | AG | 6.01 | Allele G | 3.17 |
| | | GG | 0.17 | | |
| | rs12196014 | GG | 84.27 | Allele G | 91.70 |
| | | GA | 14.86 | Allele A | 8.30 |
| | | AA | 0.87 | | |
| | rs9448863 | GG | 68.73 | Allele G | 82.69 |
| | | GA | 27.93 | Allele A | 17.31 |
| | | AA | 3.34 | | |
| | GSA-rs16891339 | AA | 96.82 | Allele A | 98.41 |
| | | AG | 3.18 | Allele G | 1.59 |
| | | GG | - | | |
| ELOVL5 | GSA-rs41273878 | CC | 97.83 | Allele C | 98.92 |
| | | CA | 2.17 | Allele A | 1.09 |
| | | AA | - | | |
| | GSA-rs72938776 | GG | 98.83 | Allele G | 99.33 |
| | | GA | 1.00 | Allele A | 0.67 |
| | | AA | 0.17 | | |
| | GSA-rs36054518 | AA | 96.99 | Allele A | 98.50 |
| | | AG | 3.01 | Allele G | 1.51 |
| | | GG | - | | |
| | GSA-rs72940713 | AA | 98.49 | Allele A | 99.25 |
| | | AG | 1.51 | Allele G | 0.75 |
| | | GG | - | | |
| | GSA-rs114271869 | AA | 97.82 | Allele A | 98.91 |
| | | AC | 2.18 | Allele C | 1.09 |
| | | CC | - | | |
| | rs 2073040 | GG | 28.52 | Allele G | 51.03 |
| | | AG | 45.02 | Allele A | 48.97 |
| | | AA | 26.46 | | |
| | rs 9370194 | CC | 72.05 | Allele C | 84.46 |
| | | CT | 24.83 | Allele T | 15.54 |
| | | TT | 3.13 | | |
| **ELOVL6** | rs11098065 | GG | 57.62 | Allele G | 75.63 |

| | | | | |
|---|---|---|---|---|
| | GA | 36.01 | Allele A | 24.37 |
| | AA | 6.37 | | |
| | AA | 96.66 | Allele A | 98.33 |
| rs17041284 | AG | 3.34 | Allele G | 1.67 |
| | GG | - | | |
| | AA | 59.13 | Allele A | 76.05 |
| rs7662161 | AG | 33.84 | Allele G | 23.95 |
| | GG | 7.04 | | |
| | GG | 97.16 | Allele G | 98.58 |
| GSA-rs77958351 | GA | 2.84 | Allele A | 1.42 |
| | AA | - | | |
| | AA | 89.08 | Allele A | 94.37 |
| rs59634436 | AC | 10.59 | Allele C | 5.63 |
| | CC | 0.34 | | |
| | GG | 96.99 | Allele G | 98.41 |
| GSA-rs78160528 | GA | 2.84 | Allele A | 1.59 |
| | AA | 0.17 | | |
| | AA | 30.49 | Allele A | 55.70 |
| rs16997129 | AG | 50.42 | Allele G | 44.31 |
| | GG | 19.10 | | |
| | GG | 97.33 | Allele G | 98.66 |
| GSA-rs3813827 | GA | 2.67 | Allele A | 1.34 |
| | AA | - | | |
| | GG | 77.72 | Allele G | 88.61 |
| rs11737840 | GA | 21.78 | Allele A | 11.39 |
| | AA | 0.50 | | |
| | AA | 86.48 | Allele A | 92.99 |
| rs10033691 | AG | 13.02 | Allele G | 7.01 |
| | GG | 0.50 | | |
| | AA | 34.29 | Allele A | 57.48 |
| GSA-rs2005701 | AG | 46.39 | Allele G | 42.52 |
| | GG | 19.33 | | |
| | GG | 96.48 | Allele G | 98.16 |
| GSA-rs76145164 | GA | 3.35 | Allele A | 1.84 |
| | AA | 0.17 | | |
| | CC | 90.80 | Allele C | 95.23 |
| GSA-rs76338299 | CA | 8.86 | Allele A | 4.77 |
| | AA | 0.33 | | |
| | GG | 48.16 | Allele G | 69.90 |
| rs6533491 | GA | 43.48 | Allele A | 30.10 |
| | AA | 8.36 | | |
| | AA | 86.14 | Allele A | 93.24 |
| rs11937052 | AG | 13.52 | Allele G | 6.76 |
| | GG | 0.33 | | |
| | GG | 26.01 | Allele G | 53.10 |
| rs11098070 | GA | 54.19 | Allele A | 46.90 |
| | AA | 19.80 | | |
| rs80343897 | AA | 59.30 | Allele A | 77.05 |

| | | | | |
|---|---|---|---|---|
| | AG | 35.51 | Allele G | 22.95 |
| | GG | 5.19 | | |
| GSA-rs114422025 | AA | 97.83 | Allele A | 98.80 |
| | AG | 2.00 | Allele G | 1.20 |
| | GG | 0.17 | | |
| rs6533495 | GG | 29.82 | Allele G | 55.19 |
| | GA | 50.75 | Allele A | 44.81 |
| | AA | 19.43 | | |
| rs28722886 | GG | 56.54 | Allele G | 76.32 |
| | GA | 39.56 | Allele A | 23.68 |
| | AA | 3.90 | | |
| rs6533497 | GG | 82.24 | Allele G | 90.20 |
| | GA | 15.91 | Allele A | 9.80 |
| | AA | 1.84 | | |
| GSA-rs77504516 | AA | 93.97 | Allele A | 96.99 |
| | AG | 6.03 | Allele G | 3.02 |
| | GG | - | | |
| rs6815102 | AA | 41.95 | Allele A | 65.10 |
| | AG | 46.31 | Allele G | 34.90 |
| | GG | 11.74 | | |
| rs4326075 | AA | 59.97 | Allele A | 77.22 |
| | AC | 34.51 | Allele C | 22.78 |
| | CC | 5.53 | | |
| GSA-rs116418972 | AA | 98.16 | Allele A | 99.08 |
| | AG | 1.84 | Allele G | 0.92 |
| | GG | - | | |
| rs11729740 | GG | 88.81 | Allele G | 94.32 |
| | GA | 11.02 | Allele A | 5.68 |
| | AA | 0.17 | | |
| rs2035415 | GG | 27.09 | Allele G | 53.43 |
| | GA | 52.68 | Allele A | 46.57 |
| | AA | 20.23 | | |
| GSA-rs17041402 | AA | 97.16 | Allele A | 98.58 |
| | AC | 2.84 | Allele C | 1.42 |
| | CC | - | | |
| rs59111930 | AA | 47.49 | Allele A | 69.06 |
| | AG | 43.14 | Allele G | 30.94 |
| | GG | 9.36 | | |
| rs74874270 | AA | 94.99 | Allele A | 97.33 |
| | AG | 4.67 | Allele G | 2.67 |
| | GG | 0.33 | | |
| rs1384331 | AA | 34.67 | Allele A | 59.72 |
| | AC | 50.08 | Allele C | 40.29 |
| | CC | 15.24 | | |
| GSA-rs72679246 | AA | 97.59 | Allele A | 98.80 |
| | CA | 2.41 | Allele C | 1.20 |
| | CC | - | | |
| rs78563565 | AA | 95.66 | Allele A | 97.83 |

| | | | | | |
|---|---|---|---|---|---|
| | | AG | 4.34 | Allele G | 2.17 |
| | | GG | - | | |
| | rs6533498 | CC | 47.49 | Allele C | 69.15 |
| | | CA | 9.20 | Allele A | 30.85 |
| | | AA | 43.31 | | |
| | rs 9997926 | CC | 91.36 | Allele C | 95.68 |
| | | CT | 8.64 | Allele T | 4.32 |
| | | TT | - | | |
| | rs 6824447 | GG | 43.47 | Allele G | 64.61 |
| | | AG | 42.27 | Allele A | 35.40 |
| | | AA | 14.26 | | |
| | rs 17041272 | CC | 83.61 | Allele C | 91.30 |
| | | CG | 15.37 | Allele G | 8.70 |
| | | GG | 1.01 | | |
| **ELOVL7** | GSA-rs75621404 | GG | 97.66 | Allele G | 98.83 |
| | | GA | 2.34 | Allele A | 1.17 |
| | | AA | - | | |
| | GSA-rs1563517 | CC | 69.35 | Allele C | 83.50 |
| | | CA | 28.31 | Allele A | 16.50 |
| | | AA | 2.35 | | |
| | GSA-rs12188996 | AA | 98.49 | Allele A | 99.24 |
| | | AC | 1.51 | Allele C | 0.76 |
| | | CC | - | | |
| | GSA-rs60258111 | GG | 98.48 | Allele G | 99.24 |
| | | GA | 1.52 | Allele A | 0.76 |
| | | AA | - | | |
| | GSA-rs16878426 | AA | 98.16 | Allele A | 99.08 |
| | | AG | 1.84 | Allele G | 0.92 |
| | | GG | - | | |
| | rs6872863 | GG | 35.01 | Allele G | 58.88 |
| | | GA | 47.74 | Allele A | 41.12 |
| | | AA | 17.25 | | |
| | GSA-rs76641655 | AA | 96.99 | Allele A | 98.50 |
| | | AC | 3.01 | Allele C | 1.50 |
| | | CC | - | | |
| | GSA-rs114011218 | AA | 97.83 | Allele A | 98.91 |
| | | AG | 2.17 | Allele G | 1.09 |
| | | GG | - | | |
| | GSA-rs115159664 | AA | 96.49 | Allele A | 98.16 |
| | | AG | 3.34 | Allele G | 1.84 |
| | | GG | 0.17 | | |
| | rs4700398 | GG | 51.93 | Allele G | 71.26 |
| | | GA | 38.66 | Allele A | 28.74 |
| | | AA | 9.41 | | |

A: Adenine; T: Thymine; G: Guanine; C: Cytosine.

**Table 2.** Association of SNPs with clinical markers of chronic non-communicable diseases.

| ELOVL2 | | | | | | | | | | | | |
|---|---|---|---|---|---|---|---|---|---|---|---|---|
| **rs8523** | | | | | | | | | | | | |
| | **Total** | | | | **Men** | | | | **Women** | | | |
| **Clinical marker** | **OR** | **95% CI** | | ***p*-value** | **OR** | **95% CI** | | ***p*-value** | **OR** | **95% CI** | | ***p*-value** |
| **H-BMI** | 1.290 | 0.912 | 1.825 | 0.151 | 1.240 | 0.754 | 2.037 | 0.398 | 1.376 | 0.844 | **2.245** | 0.202 |
| **H- Waist** | 1.338 | 0.943 | 1.899 | 0.103 | 1.409 | 0.820 | 2.421 | 0.215 | 1.222 | 0.768 | 1.947 | 0.399 |
| **H-WHI** | 0.920 | 0.662 | 1.278 | 0.620 | 1.104 | 0.683 | 1.783 | 0.688 | 0.883 | 0.535 | 1.458 | 0.628 |
| **H-WHR** | 1.161 | 0.835 | 1.613 | 0.376 | 1.140 | 0.707 | 1.840 | 0.592 | 1.179 | 0.747 | 1.859 | 0.481 |
| **H-%BF** | 0.989 | 0.709 | 1.378 | 0.946 | 1.152 | 0.710 | 1.870 | 0.569 | 0.889 | 0.561 | 1.410 | 0.619 |
| **H-Insulin** | 2.048 | 1.238 | 3.388 | 0.005 | 2.305 | 1.172 | 4.532 | 0.014 | 1.874 | 0.869 | 4.041 | 0.106 |
| **H-Glucose** | 1.481 | 0.563 | 3.892 | 0.424 | 1.881 | 0.494 | 7.157 | 0.349 | 1.165 | 0.286 | 4.747 | 0.831 |
| **H-HOMA** | 1.847 | 1.132 | 3.013 | 0.013 | 2.099 | 1.094 | 4.027 | 0.024 | 1.719 | 0.809 | 3.654 | 0.157 |
| **H-Cholesterol** | 2.628 | 1.400 | 4.935 | 0.002 | 3.154 | 1.408 | 7.061 | 0.004 | 2.222 | 0.800 | 6.169 | 0.118 |
| **H-Triglycerides** | 1.037 | 0.693 | 1.552 | 0.861 | 1.289 | 0.750 | 2.216 | 0.359 | 0.848 | 0.458 | 1.571 | 0.602 |
| **L-HDL** | 0.915 | 0.652 | 1.283 | 0.606 | 0.809 | 0.465 | 1.407 | 0.454 | 0.892 | 0.570 | 1.398 | 0.620 |
| **H-LDL** | 1.424 | 0.657 | 3.085 | 0.369 | 2.210 | 0.816 | 5.986 | 0.112 | 0.767 | 0.212 | 2.775 | 0.687 |
| **rs3734396** | | | | | | | | | | | | |
| | **Total** | | | | **Men** | | | | **Women** | | | |
| **Clinical marker** | **OR** | **95% CI** | | ***p*-value** | **OR** | **95% CI** | | ***p*-value** | **OR** | **95% CI** | | ***p*-value** |
| **H-BMI** | 0.747 | 0.432 | 1.291 | 0.296 | 0.597 | 0.278 | 1.282 | 0.183 | 0.941 | 0.429 | 2.065 | 0.880 |
| **H- Waist** | 0.715 | 0.407 | 1.257 | 0.243 | 0.754 | 0.329 | 1.725 | 0.504 | 0.726 | 0.332 | 1.586 | 0.422 |
| **H-WHI** | 0.847 | 0.511 | 1.402 | 0.518 | 0.821 | 0.416 | 1.618 | 0.569 | 0.668 | 0.279 | 1.602 | 0.365 |
| **H-WHR** | 0.769 | 0.463 | 1.278 | 0.311 | 0.618 | 0.303 | 1.259 | 0.184 | 0.976 | 0.470 | 2.027 | 0.949 |
| **H-%BF** | 0.856 | 0.518 | 1.413 | 0.543 | 0.655 | 0.326 | 1.317 | 0.234 | 1.114 | 0.540 | 2.299 | 0.772 |
| **H-Insulin** | 1.107 | 0.528 | 2.320 | 0.789 | 0.594 | 0.193 | 1.829 | 0.362 | 2.076 | 0.761 | 5.666 | 0.148 |
| **H-Glucose** | | | | | | | | | | | | |
| **H-HOMA** | 1.170 | 0.568 | 2.411 | 0.671 | 0.880 | 0.318 | 2.431 | 0.806 | 1.607 | 0.571 | 4.521 | 0.366 |
| **H-Cholesterol** | 1.286 | 0.553 | 2.988 | 0.559 | 1.019 | 0.335 | 3.106 | 0.873 | 1.690 | 0.463 | 6.167 | 0.423 |
| **H-Triglycerides** | 0.742 | 0.386 | 1.427 | 0.371 | 0.881 | 0.397 | 1.956 | 0.756 | 0.474 | 0.139 | 1.614 | 0.224 |

| | | | | | | | | | | | | |
|---|---|---|---|---|---|---|---|---|---|---|---|---|
| **L-HDL** | 0.983 | 0.590 | 1.640 | 0.949 | 1.231 | 0.578 | 2.620 | 0.591 | 0.906 | 0.442 | 1.855 | 0.787 |
| **H-LDL** | 2.149 | 0.838 | 5.515 | 0.104 | 1.393 | 0.381 | 5.087 | 0.615 | 3.733 | 0.918 | 15.177 | 0.050 |

| **rs17606561** | | | | | | | | | | | | |
|---|---|---|---|---|---|---|---|---|---|---|---|---|
| | **Total** | | | | **Men** | | | | **Women** | | | |
| **Clinical marker** | **OR** | **95% CI** | | ***p*-value** | **OR** | **95% CI** | | ***p*-value** | **OR** | **95% CI** | | ***p*-value** |
| **H-BMI** | 0.826 | 0.575 | 1.186 | 0.301 | 0.841 | 0.498 | 1.417 | 0.516 | 0.826 | 0.499 | 1.367 | 0.458 |
| **H- Waist** | 0.692 | 0.477 | 1.004 | 0.052 | 0.650 | 0.359 | 1.179 | 0.155 | 0.679 | 0.418 | 1.104 | 0.188 |
| **H-WHI** | 1.136 | 0.810 | 1.594 | 0.461 | 1.424 | 0.860 | 2.360 | 0.170 | 1.066 | 0.640 | 1.777 | 0.806 |
| **H-WHR** | 0.849 | 0.604 | 1.194 | 0.347 | 0.904 | 0.549 | 1.486 | 0.691 | 0.804 | 0.503 | 1.284 | 0.362 |
| **H-%BF** | 0.954 | 0.678 | 1.343 | 0.790 | 0.885 | 0.536 | 1.460 | 0.633 | 1.047 | 0.655 | 1.675 | 0.848 |
| **H-Insulin** | 0.602 | 0.346 | 1.049 | 0.071 | 0.291 | 0.122 | 0.693 | 0.004 | 1.298 | 0.600 | 2.808 | 0.509 |
| **H-Glucose** | 0.371 | 0.105 | 1.306 | 0.109 | 0.239 | 0.030 | 1.943 | 0.148 | 0.527 | 0.105 | 2.655 | 0.432 |
| **H-HOMA** | 0.521 | 0.298 | 0.914 | 0.021 | 0.204 | 0.077 | 0.539 | 0.001 | 1.241 | 0.583 | 2.645 | 0.576 |
| **H-Cholesterol** | 0.603 | 0.305 | 1.191 | 0.142 | 0.732 | 0.311 | 1.719 | 0.474 | 0.476 | 0.152 | 1.496 | 0.196 |
| **H-Triglycerides** | 0.625 | 0.404 | 0.968 | 0.034 | 0.698 | 0.388 | 1.256 | 0.231 | 0.574 | 0.295 | 1.116 | 0.100 |
| **L-HDL** | 1.287 | 0.911 | 1.818 | 0.152 | 1.261 | 0.723 | 2.200 | 0.416 | 1.226 | 0.775 | 1.940 | 0.385 |
| **H-LDL** | 0.881 | 0.389 | 1.997 | 0.762 | 0.815 | 0.279 | 2.384 | 0.710 | 1.072 | 0.296 | 3.882 | 0.915 |

| **rs3734398** | | | | | | | | | | | | |
|---|---|---|---|---|---|---|---|---|---|---|---|---|
| | **Total** | | | | **Men** | | | | **Women** | | | |
| **Clinical marker** | **OR** | **95% CI** | | ***p*-value** | **OR** | **95% CI** | | ***p*-value** | **OR** | **95% CI** | | ***p*-value** |
| **H-BMI** | 1.441 | 1.020 | 2.036 | 0.038 | 1.332 | 0.815 | 2.175 | 0.253 | 1.583 | 0.970 | 2.583 | 0.065 |
| **H- Waist** | 1.304 | 0.920 | 1.847 | 0.135 | 1.360 | 0.795 | 2.329 | 0.263 | 1.223 | 0.769 | 1.945 | 0.396 |
| **H-WHI** | 0.857 | 0.618 | 1.187 | 0.354 | 0.965 | 0.603 | 1.546 | 0.883 | 0.815 | 0.495 | 1.343 | 0.423 |
| **H-WHR** | 1.165 | 0.841 | 1.615 | 0.359 | 1.119 | 0.699 | 1.792 | 0.641 | 1.211 | 0.769 | 1.907 | 0.410 |
| **H-%BF** | 1.060 | 0.763 | 1.473 | 0.729 | 1.181 | 0.734 | 1.900 | 0.495 | 0.979 | 0.619 | 1.548 | 0.928 |
| **H-Insulin** | 1.894 | 1.146 | 3.130 | 0.012 | 2.222 | 1.133 | 4.360 | 0.019 | 1.618 | 0.751 | 3.486 | 0.228 |
| **H-Glucose** | 1.282 | 0.488 | 3.369 | 0.614 | 1.540 | 0.405 | 5.857 | 0.525 | 1.061 | 0.261 | 4.321 | 0.934 |
| **H-HOMA** | 1.780 | 1.088 | 2.912 | 0.020 | 2.080 | 1.079 | 4.009 | 0.027 | 1.541 | 0.725 | 3.274 | 0.260 |
| **H-Cholesterol** | 2.341 | 1.251 | 4.378 | 0.006 | 3.010 | 1.320 | 6.864 | 0.006 | 1.717 | 0.648 | 4.553 | 0.274 |

| | | | | | | | | | | | | |
|---|---|---|---|---|---|---|---|---|---|---|---|---|
| **H-Triglycerides** | 1.269 | 0.852 | 1.889 | 0.242 | 1.506 | 0.882 | 2.573 | 0.133 | 1.071 | 0.585 | 1.962 | 0.824 |
| **L-HDL** | 0.934 | 0.669 | 1.306 | 0.692 | 0.894 | 0.522 | 1.530 | 0.683 | 0.906 | 0.580 | 1.415 | 0.664 |
| **H-LDL** | 1.685 | 0.769 | 3.695 | 0.189 | 2.342 | 0.842 | 6.515 | 0.095 | 1.062 | 0.301 | 3.743 | 0.926 |
| | | | | | | **rs2281591** | | | | | | |
| | **Total** | | | | **Men** | | | | **Women** | | | |
| **Clinical marker** | **OR** | **95% CI** | | ***p*-value** | **OR** | **95% CI** | | ***p*-value** | **OR** | **95% CI** | | ***p*-value** |
| **H-BMI** | 0.856 | 0.601 | 1.219 | 0.390 | 0.925 | 0.558 | 1.532 | 0.762 | 0.802 | 0.488 | 1.320 | 0.387 |
| **H- Waist** | 0.641 | 0.445 | 0.924 | 0.017 | 0.650 | 0.367 | 1.153 | 0.140 | 0.610 | 0.377 | 0.988 | 0.044 |
| **H-WHI** | 1.078 | 0.772 | 1.504 | 0.660 | 1.365 | 0.837 | 2.225 | 0.213 | 0.917 | 0.551 | 1.524 | 0.739 |
| **H-WHR** | 0.833 | 0.596 | 1.164 | 0.286 | 1.001 | 0.617 | 1.623 | 0.998 | 0.704 | 0.443 | 1.121 | 0.140 |
| **H-%BF** | 0.938 | 0.670 | 1.312 | 0.708 | 0.910 | 0.559 | 1.482 | 0.706 | 0.976 | 0.613 | 1.554 | 0.918 |
| **H-Insulin** | 0.517 | 0.297 | 0.899 | 0.018 | 0.240 | 0.101 | 0.572 | 0.001 | 1.136 | 0.526 | 2.454 | 0.746 |
| **H-Glucose** | 0.459 | 0.148 | 1.425 | 0.168 | 0.451 | 0.092 | 2.213 | 0.317 | 0.472 | 0.094 | 2.377 | 0.354 |
| **H-HOMA** | 0.479 | 0.276 | 0.831 | 0.008 | 0.204 | 0.083 | 0.503 | 0.001 | 1.108 | 0.521 | 2.358 | 0.791 |
| **H-Cholesterol** | 0.576 | 0.296 | 1.119 | 0.100 | 0.702 | 0.307 | 1.603 | 0.401 | 0.425 | 0.135 | 1.336 | 0.133 |
| **H-Triglycerides** | 0.767 | 0.506 | 1.164 | 0.213 | 0.912 | 0.523 | 1.589 | 0.746 | 0.632 | 0.332 | 1.202 | 0.160 |
| **L-HDL** | 1.189 | 0.846 | 1.672 | 0.319 | 1.157 | 0.668 | 2.001 | 0.604 | 1.175 | 0.746 | 1.850 | 0.488 |
| **H-LDL** | 1.048 | 0.478 | 2.300 | 0.907 | 0.874 | 0.314 | 2.435 | 0.797 | 1.459 | 0.414 | 5.148 | 0.556 |
| | | | | | | **rs2236212** | | | | | | |
| | **Total** | | | | **Men** | | | | **Women** | | | |
| **Clinical marker** | **OR** | **95% CI** | | ***p*-value** | **OR** | **95% CI** | | ***p*-value** | **OR** | **95% CI** | | ***p*-value** |
| **H-BMI** | 1.204 | 0.849 | 1.707 | 0.297 | 0.986 | 0.604 | 1.611 | 0.956 | 1.492 | 0.904 | 2.463 | 0.117 |
| **H- Waist** | 1.091 | 0.768 | 1.550 | 0.628 | 1.092 | 0.636 | 1.874 | 0.751 | 1.063 | 0.665 | 1.699 | 0.800 |
| **H-WHI** | 0.790 | 0.569 | 1.096 | 0.158 | 0.850 | 0.531 | 1.363 | 0.502 | 0.748 | 0.454 | 1.235 | 0.257 |
| **H-WHR** | 0.981 | 0.706 | 1.362 | 0.909 | 0.887 | 0.554 | 1.419 | 0.617 | 1.080 | 0.682 | 1.710 | 0.743 |
| **H-%BF** | 0.947 | 0.680 | 1.318 | 0.747 | 0.903 | 0.562 | 1.451 | 0.675 | 1.009 | 0.635 | 1.605 | 0.969 |
| **H-Insulin** | 1.922 | 1.135 | 3.254 | 0.014 | 2.198 | 1.088 | 4.441 | 0.026 | 1.688 | 0.755 | 3.774 | 0.201 |
| **H-Glucose** | 0.877 | 0.334 | 2.306 | 0.791 | 1.029 | 0.271 | 3.915 | 0.966 | 0.741 | 0.182 | 3.020 | 0.676 |
| **H-HOMA** | 1.995 | 1.184 | 3.361 | 0.009 | 2.415 | 1.200 | 4.859 | 0.012 | 1.643 | 0.744 | 3.626 | 0.217 |

| | | | | | | | | | | | | |
|---|---|---|---|---|---|---|---|---|---|---|---|---|
| **H-Cholesterol** | 2.432 | 1.236 | 4.785 | 0.008 | 2.842 | 1.173 | 6.886 | 0.016 | 2.017 | 0.701 | 5.804 | 0.187 |
| **H-Triglycerides** | 1.162 | 0.776 | 1.739 | 0.467 | 1.519 | 0.879 | 2.625 | 0.136 | 0.855 | 0.466 | 1.571 | 0.615 |
| **L-HDL** | 0.911 | 0.652 | 1.275 | 0.588 | 0.894 | 0.524 | 1.526 | 0.683 | 0.880 | 0.561 | 1.381 | 0.580 |
| **H-LDL** | 2.856 | 1.136 | 7.181 | 0.020 | 2.824 | 0.898 | 8.882 | 0.065 | 3.082 | 0.644 | 14.759 | 0.140 |

| | | | | | | | | | | | | |
|---|---|---|---|---|---|---|---|---|---|---|---|---|
| | | | | | | **rs3798713** | | | | | | |
| | **Total** | | | | **Men** | | | | **Women** | | | |
| **Clinical marker** | **OR** | **95% CI** | | ***p-value*** | **OR** | **95% CI** | | ***p-value*** | **OR** | **95% CI** | | ***p-value*** |
| **H-BMI** | 1.165 | 0.822 | 1.650 | 0.391 | 0.945 | 0.578 | 1.544 | 0.821 | 1.454 | 0.883 | 2.394 | 0.141 |
| **H- Waist** | 1.122 | 0.790 | 1.595 | 0.520 | 1.199 | 0.697 | 2.061 | 0.513 | 1.046 | 0.655 | 1.671 | 0.850 |
| **H-WHI** | 0.797 | 0.574 | 1.106 | 0.174 | 0.881 | 0.549 | 1.412 | 0.599 | 0.728 | 0.441 | 1.200 | 0.214 |
| **H-WHR** | 0.975 | 0.702 | 1.354 | 0.882 | 0.883 | 0.551 | 1.414 | 0.606 | 1.072 | 0.678 | 1.695 | 0.768 |
| **H-%BF** | 0.946 | 0.680 | 1.317 | 0.743 | 0.904 | 0.562 | 1.454 | 0.678 | 1.005 | 0.633 | 1.596 | 0.983 |
| **H-Insulin** | 1.644 | 0.978 | 2.762 | 0.059 | 1.631 | 0.819 | 3.247 | 0.163 | 1.724 | 0.771 | 3.854 | 0.183 |
| **H-Glucose** | 0.702 | 0.267 | 1.846 | 0.472 | 0.658 | 0.173 | 2.502 | 0.538 | 0.762 | 0.187 | 3.102 | 0.704 |
| **H-HOMA** | 1.765 | 1.058 | 2.944 | 0.028 | 1.888 | 0.959 | 3.715 | 0.064 | 1.694 | 0.768 | 3.739 | 0.189 |
| **H-Cholesterol** | 2.485 | 1.263 | 4.888 | 0.007 | 2.885 | 1.191 | 6.990 | 0.015 | 2.074 | 0.721 | 5.966 | 0.169 |
| **H-Triglycerides** | 1.121 | 0.749 | 1.678 | 0.579 | 1.386 | 0.803 | 2.393 | 0.242 | 0.882 | 0.481 | 1.620 | 0.688 |
| **L-HDL** | 0.927 | 0.663 | 1.298 | 0.661 | 0.947 | 0.553 | 1.621 | 0.842 | 0.877 | 0.559 | 1.375 | 0.569 |
| **H-LDL** | 2.915 | 1.159 | 7.330 | 0.018 | 2.864 | 0.910 | 9.007 | 0.062 | 3.167 | 0.661 | 15.162 | 0.130 |

| | | | | | | | | | | | | |
|---|---|---|---|---|---|---|---|---|---|---|---|---|
| | | | | | | **rs7765206** | | | | | | |
| | **Total** | | | | **Men** | | | | **Women** | | | |
| **Clinical marker** | **OR** | **95% CI** | | ***p-value*** | **OR** | **95% CI** | | ***p-value*** | **OR** | **95% CI** | | ***p-value*** |
| **H-BMI** | 1.115 | 0.523 | 2.376 | 0.778 | 1.034 | 0.337 | 3.175 | 0.953 | 1.201 | 0.431 | 3.351 | 0.727 |
| **H- Waist** | 0.520 | 0.210 | 1.291 | 0.153 | 0.223 | 0.029 | 1.736 | 0.119 | 0.706 | 0.242 | 2.058 | 0.523 |
| **H-WHI** | 0.540 | 0.244 | 1.195 | 0.124 | 0.530 | 0.179 | 1.568 | 0.246 | 0.537 | 0.150 | 1.917 | 0.333 |
| **H-WHR** | 0.830 | 0.395 | 1.743 | 0.623 | 0.994 | 0.336 | 2.942 | 0.991 | 0.711 | 0.256 | 1.976 | 0.513 |
| **H-%BF** | 0.905 | 0.433 | 1.890 | 0.790 | 0.912 | 0.311 | 2.674 | 0.868 | 0.904 | 0.328 | 2.496 | 0.847 |
| **H-Insulin** | 0.226 | 0.030 | 1.718 | 0.117 | 0.760 | 0.702 | 0.823 | 0.141 | 0.503 | 0.063 | 4.009 | 0.511 |
| **H-Glucose** | 0.970 | 0.956 | 0.984 | 0.329 | 0.967 | 0.946 | 0.988 | 0.493 | 0.973 | 0.954 | 0.992 | 0.492 |

| | | | | | | | | | | | | |
|---|---|---|---|---|---|---|---|---|---|---|---|---|
| **H-HOMA** | 0.229 | 0.031 | 1.711 | 0.117 | 0.815 | 0.767 | 0.865 | 0.101 | 0.590 | 0.075 | 4.651 | 0.614 |
| **H-Cholesterol** | 0.378 | 0.050 | 2.837 | 0.327 | 0.676 | 0.085 | 5.362 | 0.710 | 0.939 | 0.912 | 0.967 | 0.295 |
| **H-Triglycerides** | 1.939 | 0.888 | 4.233 | 0.092 | 2.364 | 0.791 | 7.059 | 0.114 | 1.659 | 0.518 | 5.313 | 0.391 |
| **L-HDL** | 1.299 | 0.623 | 2.705 | 0.485 | 1.212 | 0.368 | 3.989 | 0.753 | 1.325 | 0.497 | 3.529 | 0.574 |
| **H-LDL** | 1.498 | 0.338 | 6.633 | 0.593 | 1.240 | 0.153 | 10.086 | 0.841 | 1.979 | 0.236 | 16.596 | 0.523 |

**rs9295757**

| | **Total** | | | | **Men** | | | | **Women** | | | |
|---|---|---|---|---|---|---|---|---|---|---|---|---|
| **Clinical marker** | **OR** | **95% CI** | | ***p*-value** | **OR** | **95% CI** | | ***p*-value** | **OR** | **95% CI** | | ***p*-value** |
| **H-BMI** | 0.906 | 0.633 | 1.297 | 0.591 | 0.993 | 0.595 | 1.655 | 0.978 | 0.840 | 0.507 | 1.391 | 0.499 |
| **H- Waist** | 0.740 | 0.512 | 1.070 | 0.109 | 0.773 | 0.435 | 1.373 | 0.381 | 0.688 | 0.423 | 1.119 | 0.132 |
| **H-WHI** | 1.235 | 0.882 | 1.730 | 0.219 | 1.603 | 0.971 | 2.647 | 0.065 | 1.087 | 0.652 | 1.814 | 0.750 |
| **H-WHR** | 0.936 | 0.667 | 1.312 | 0.701 | 1.026 | 0.629 | 1.674 | 0.919 | 0.861 | 0.539 | 1.375 | 0.533 |
| **H-%BF** | 1.011 | 0.720 | 1.421 | 0.948 | 0.980 | 0.598 | 1.606 | 0.937 | 1.060 | 0.662 | 1.697 | 0.809 |
| **H-Insulin** | 0.774 | 0.456 | 1.314 | 0.343 | 0.501 | 0.236 | 1.062 | 0.069 | 1.306 | 0.603 | 2.828 | 0.499 |
| **H-Glucose** | 0.517 | 0.166 | 1.606 | 0.247 | 0.517 | 0.105 | 2.535 | 0.410 | 0.524 | 0.104 | 2.641 | 0.428 |
| **H-HOMA** | 0.678 | 0.399 | 1.152 | 0.150 | 0.403 | 0.184 | 0.881 | 0.020 | 1.234 | 0.579 | 2.629 | 0.587 |
| **H-Cholesterol** | 0.562 | 0.285 | 1.107 | 0.092 | 0.676 | 0.288 | 1.586 | 0.367 | 0.437 | 0.140 | 1.361 | 0.144 |
| **H-Triglycerides** | 0.692 | 0.451 | 1.062 | 0.091 | 0.824 | 0.467 | 1.455 | 0.507 | 0.570 | 0.293 | 1.109 | 0.096 |
| **L-HDL** | 1.229 | 0.871 | 1.734 | 0.241 | 1.238 | 0.713 | 2.149 | 0.450 | 1.175 | 0.742 | 1.859 | 0.494 |
| **H-LDL** | 0.848 | 0.374 | 1.922 | 0.694 | 0.755 | 0.258 | 2.207 | 0.608 | 1.067 | 0.295 | 3.861 | 0.922 |

**rs3798721**

| | **Total** | | | | **Men** | | | | **Women** | | | |
|---|---|---|---|---|---|---|---|---|---|---|---|---|
| **Clinical marker** | **OR** | **95% CI** | | ***p*-value** | **OR** | **95% CI** | | ***p*-value** | **OR** | **95% CI** | | ***p*-value** |
| **H-BMI** | 1.015 | 0.612 | 1.685 | 0.576 | 0.833 | 0.510 | 1.361 | 0.953 | 0.904 | 0.636 | 1.285 | 0.468 |
| **H- Waist** | 0.822 | 0.576 | 1.175 | 0.282 | 0.816 | 0.464 | 1.434 | 0.480 | 0.761 | 0.476 | 1.217 | 0.255 |
| **H-WHI** | 1.114 | 0.800 | 1.552 | 0.523 | 1.629 | 0.992 | 2.676 | 0.053 | 0.958 | 0.580 | 1.581 | 0.867 |
| **H-WHR** | 0.991 | 0.711 | 1.382 | 0.959 | 1.046 | 0.643 | 1.702 | 0.856 | 0.945 | 0.599 | 1.492 | 0.810 |
| **H-%BF** | 0.996 | 0.713 | 1.391 | 0.983 | 0.973 | 0.596 | 1.590 | 0.915 | 1.057 | 0.667 | 1.676 | 0.813 |
| **H-Insulin** | 0.886 | 0.530 | 1.480 | 0.644 | 0.524 | 0.247 | 1.115 | 0.091 | 1.752 | 0.817 | 3.759 | 0.148 |

| | | | | | | | | | | | | |
|---|---|---|---|---|---|---|---|---|---|---|---|---|
| **H-Glucose** | 0.430 | 0.139 | 1.336 | 0.134 | 0.204 | 0.025 | 1.651 | 0.101 | 0.732 | 0.172 | 3.120 | 0.674 |
| **H-HOMA** | 0.692 | 0.415 | 1.156 | 0.159 | 0.381 | 0.174 | 0.833 | 0.013 | 1.419 | 0.673 | 2.992 | 0.358 |
| **H-Cholesterol** | 0.602 | 0.314 | 1.154 | 0.124 | 0.614 | 0.262 | 1.440 | 0.260 | 0.656 | 0.236 | 1.821 | 0.417 |
| **H-Triglycerides** | 0.742 | 0.490 | 1.123 | 0.158 | 0.892 | 0.510 | 1.562 | 0.691 | 0.646 | 0.345 | 1.209 | 0.171 |
| **L-HDL** | 1.214 | 0.865 | 1.703 | 0.263 | 1.135 | 0.654 | 1.971 | 0.653 | 1.136 | 0.725 | 1.780 | 0.579 |
| **H-LDL** | 0.834 | 0.375 | 1.853 | 0.655 | 0.689 | 0.236 | 2.013 | 0.495 | 1.239 | 0.351 | 4.369 | 0.740 |
| **rs16870899** | | | | | | | | | | | | |
| | **Total** | | | | **Men** | | | | **Women** | | | |
| **Clinical marker** | **OR** | **95% CI** | | ***p*-value** | **OR** | **95% CI** | | ***p*-value** | **OR** | **95% CI** | | ***p*-value** |
| **H-BMI** | 1.274 | 0.567 | 2.862 | 0.558 | 1.170 | 0.372 | 3.678 | 0.789 | 1.382 | 0.440 | 4.342 | 0.580 |
| **H- Waist** | 0.519 | 0.193 | 1.398 | 0.188 | 0.243 | 0.031 | 1.902 | 0.146 | 0.758 | 0.228 | 2.521 | 0.652 |
| **H-WHI** | 0.487 | 0.202 | 1.177 | 0.104 | 0.439 | 0.140 | 1.376 | 0.149 | 0.457 | 0.099 | 2.104 | 0.305 |
| **H-WHR** | 0.822 | 0.367 | 1.844 | 0.635 | 1.143 | 0.374 | 3.490 | 0.815 | 0.577 | 0.174 | 1.918 | 0.366 |
| **H-%BF** | 1.132 | 0.507 | 2.525 | 0.762 | 1.073 | 0.351 | 3.279 | 0.902 | 1.177 | 0.371 | 3.737 | 0.783 |
| **H-Insulin** | 0.271 | 0.035 | 2.078 | 0.180 | 0.759 | 0.701 | 0.822 | 0.115 | 0.771 | 0.093 | 6.386 | 0.810 |
| **H-Glucose** | 1.393 | 0.178 | 10.922 | 0.752 | 2.781 | 0.321 | 24.064 | 0.334 | 0.973 | 0.955 | 0.992 | 0.551 |
| **H-HOMA** | 0.553 | 0.127 | 2.400 | 0.423 | 0.375 | 0.048 | 2.960 | 0.336 | 0.840 | 0.104 | 6.793 | 0.871 |
| **H-Cholesterol** | 0.458 | 0.061 | 3.459 | 0.439 | 0.735 | 0.092 | 5.867 | 0.772 | 0.940 | 0.913 | 0.967 | 0.363 |
| **H-Triglycerides** | 1.789 | 0.759 | 4.219 | 0.179 | 1.940 | 0.614 | 6.133 | 0.253 | 1.602 | 0.425 | 6.041 | 0.484 |
| **L-HDL** | 1.113 | 0.496 | 2.499 | 0.795 | 0.896 | 0.240 | 3.348 | 0.870 | 1.371 | 0.450 | 4.177 | 0.579 |
| **H-LDL** | 1.827 | 0.409 | 8.163 | 0.424 | 1.349 | 0.165 | 11.033 | 0.780 | 2.676 | 0.313 | 22.861 | 0.352 |
| **rs3798722** | | | | | | | | | | | | |
| | **Total** | | | | **Men** | | | | **Women** | | | |
| **Clinical marker** | **OR** | **95% CI** | | ***p*-value** | **OR** | **95% CI** | | ***p*-value** | **OR** | **95% CI** | | ***p*-value** |
| **H-BMI** | 0.925 | 0.654 | 1.309 | 0.661 | 1.022 | 0.621 | 1.681 | 0.932 | 0.860 | 0.528 | 1.400 | 0.545 |
| **H- Waist** | 0.751 | 0.527 | 1.070 | 0.113 | 0.715 | 0.409 | 1.248 | 0.238 | 0.723 | 0.453 | 1.154 | 0.175 |
| **H-WHI** | 1.050 | 0.756 | 1.458 | 0.770 | 1.472 | 0.908 | 2.388 | 0.117 | 0.884 | 0.536 | 1.457 | 0.630 |
| **H-WHR** | 0.986 | 0.710 | 1.370 | 0.934 | 1.126 | 0.698 | 1.816 | 0.628 | 0.875 | 0.555 | 1.379 | 0.566 |
| **H-%BF** | 1.000 | 0.718 | 1.393 | 0.998 | 0.989 | 0.610 | 1.601 | 0.963 | 1.043 | 0.659 | 1.651 | 0.857 |

| | | | | | | | | | | | | |
|---|---|---|---|---|---|---|---|---|---|---|---|---|
| H-Insulin | 0.749 | 0.449 | 1.251 | 0.270 | 0.422 | 0.199 | 0.894 | 0.022 | 1.543 | 0.720 | 3.307 | 0.265 |
| H-Glucose | 0.513 | 0.178 | 1.475 | 0.209 | 0.400 | 0.082 | 1.961 | 0.245 | 0.658 | 0.155 | 2.804 | 0.271 |
| H-HOMA | 0.633 | 0.381 | 1.052 | 0.076 | 0.357 | 0.168 | 0.759 | 0.006 | 1.273 | 0.604 | 2.682 | 0.527 |
| H-Cholesterol | 0.583 | 0.308 | 1.104 | 0.095 | 0.617 | 0.270 | 1.407 | 0.249 | 0.588 | 0.212 | 1.632 | 0.305 |
| H-Triglycerides | 0.893 | 0.596 | 1.337 | 0.582 | 1.068 | 0.620 | 1.841 | 0.813 | 0.770 | 0.418 | 1.420 | 0.404 |
| L-HDL | 1.215 | 0.868 | 1.700 | 0.257 | 1.068 | 0.620 | 1.841 | 0.813 | 1.213 | 0.776 | 1.898 | 0.398 |
| H-LDL | 1.003 | 0.461 | 2.182 | 0.994 | 0.771 | 0.277 | 2.148 | 0.620 | 1.691 | 0.468 | 6.116 | 0.420 |
| **rs9393903** | | | | | | | | | | | | |
| | **Total** | | | | **Men** | | | | **Women** | | | |
| **Clinical marker** | **OR** | **95% CI** | | ***p*-value** | **OR** | **95% CI** | | ***p*-value** | **OR** | **95% CI** | | ***p*-value** |
| H-BMI | 0.865 | 0.603 | 1.240 | 0.430 | 0.841 | 0.498 | 1.417 | 0.516 | 0.904 | 0.549 | 1.490 | 0.694 |
| H- Waist | 0.755 | 0.522 | 1.091 | 0.134 | 0.650 | 0.359 | 1.179 | 0.155 | 0.781 | 0.483 | 1.262 | 0.314 |
| H-WHI | 1.210 | 0.864 | 1.697 | 0.268 | 1.523 | 0.917 | 2.528 | 0.104 | 1.174 | 0.707 | 1.950 | 0.536 |
| H-WHR | 0.933 | 0.665 | 1.310 | 0.691 | 0.964 | 0.586 | 1.583 | 0.884 | 0.908 | 0.570 | 1.446 | 0.686 |
| H-%BF | 1.015 | 0.722 | 1.427 | 0.934 | 0.885 | 0.536 | 1.460 | 0.633 | 1.178 | 0.738 | 1.882 | 0.493 |
| H-Insulin | 0.652 | 0.377 | 1.127 | 0.124 | 0.299 | 0.125 | 0.713 | 0.004 | 1.480 | 0.688 | 3.184 | 0.316 |
| H-Glucose | 0.365 | 0.104 | 1.286 | 0.103 | 0.239 | 0.030 | 1.943 | 0.148 | 0.513 | 0.102 | 2.582 | 0.411 |
| H-HOMA | 0.560 | 0.322 | 0.972 | 0.038 | 0.204 | 0.077 | 0.539 | 0.001 | 1.415 | 0.668 | 2.999 | 0.364 |
| H-Cholesterol | 0.594 | 0.301 | 1.173 | 0.130 | 0.732 | 0.311 | 1.719 | 0.474 | 0.463 | 0.147 | 1.454 | 0.179 |
| H-Triglycerides | 0.678 | 0.440 | 1.043 | 0.076 | 0.698 | 0.388 | 1.256 | 0.231 | 0.695 | 0.365 | 1.322 | 0.267 |
| L-HDL | 1.380 | 0.978 | 1.947 | 0.067 | 1.261 | 0.723 | 2.200 | 0.416 | 1.371 | 0.867 | 2.168 | 0.177 |
| H-LDL | 0.868 | 0.383 | 1.967 | 0.734 | 0.815 | 0.279 | 2.384 | 0.710 | 1.043 | 0.288 | 3.774 | 0.949 |
| **rs4532436** | | | | | | | | | | | | |
| | **Total** | | | | **Men** | | | | **Women** | | | |
| **Clinical marker** | **OR** | **95% CI** | | ***p*-value** | **OR** | **95% CI** | | ***p*-value** | **OR** | **95% CI** | | ***p*-value** |
| H-BMI | 1.473 | 1.040 | 2.087 | 0.029 | 1.395 | 0.850 | 2.291 | 0.138 | 1.581 | 0.967 | 2.586 | 0.067 |
| H- Waist | 1.448 | 1.019 | 2.058 | 0.039 | 1.684 | 0.978 | 2.899 | 0.059 | 1.247 | 0.781 | 1.990 | 0.356 |
| H-WHI | 0.905 | 0.651 | 1.258 | 0.552 | 1.068 | 0.662 | 1.721 | 0.789 | 0.845 | 0.510 | 1.399 | 0.514 |
| H-WHR | 1.208 | 0.869 | 1.679 | 0.262 | 1.216 | 0.755 | 1.958 | 0.423 | 1.199 | 0.759 | 1.896 | 0.438 |

| | | | | | | | | | | | | |
|---|---|---|---|---|---|---|---|---|---|---|---|---|
| **H-%BF** | 1.180 | 0.846 | 1.647 | 0.329 | 1.356 | 0.836 | 2.199 | 0.218 | 1.066 | 0.672 | 1.694 | 0.286 |
| **H-Insulin** | 2.248 | 1.357 | 3.722 | 0.001 | 2.924 | 1.476 | 5.792 | 0.002 | 1.670 | 0.778 | 3.584 | 0.187 |
| **H-Glucose** | 1.442 | 0.549 | 3.791 | 0.457 | 1.761 | 0.463 | 6.700 | 0.403 | 1.175 | 0.288 | 4.787 | 0.822 |
| **H-HOMA** | 2.025 | 1.237 | 3.315 | 0.004 | 2.710 | 1.395 | 5.267 | 0.003 | 1.486 | 0.703 | 3.143 | 0.299 |
| **H-Cholesterol** | 2.654 | 1.418 | 4.966 | 0.002 | 2.935 | 1.311 | 6.568 | 0.007 | 2.465 | 0.900 | 6.749 | 0.071 |
| **H-Triglycerides** | 1.143 | 0.767 | 1.705 | 0.512 | 1.332 | 0.778 | 2.279 | 0.297 | 0.996 | 0.542 | 1.830 | 0.990 |
| **L-HDL** | 0.864 | 0.615 | 1.213 | 0.398 | 0.805 | 0.464 | 1.395 | 0.441 | 0.834 | 0.531 | 1.310 | 0.432 |
| **H-LDL** | 1.386 | 0.640 | 3.003 | 0.406 | 2.065 | 0.763 | 5.591 | 0.147 | 0.774 | 0.214 | 2.798 | 0.696 |

| | | | | | | | | | | | | |
|---|---|---|---|---|---|---|---|---|---|---|---|---|
| | | | | | | **rs12195587** | | | | | | |
| | **Total** | | | | **Men** | | | | **Women** | | | |
| **Clinical marker** | **OR** | **95% CI** | | ***p-value*** | **OR** | **95% CI** | | ***p-value*** | **OR** | **95% CI** | | ***p-value*** |
| **H-BMI** | 1.282 | 0.789 | 2.084 | 0.316 | 1.285 | 0.647 | 2.552 | 0.476 | 1.276 | 0.641 | 2.540 | 0.488 |
| **H- Waist** | 1.420 | 0.874 | 2.306 | 0.156 | 1.808 | 0.884 | 3.695 | 0.102 | 1.183 | 0.608 | 2.301 | 0.622 |
| **H-WHI** | 1.135 | 0.710 | 1.815 | 0.598 | 1.259 | 0.632 | 2.506 | 0.513 | 1.022 | 0.497 | 2.102 | 0.954 |
| **H-WHR** | 1.225 | 0.767 | 1.956 | 0.396 | 1.382 | 0.707 | 2.702 | 0.344 | 1.092 | 0.567 | 2.102 | 0.794 |
| **H-%BF** | 1.184 | 0.739 | 1.898 | 0.482 | 1.624 | 0.815 | 3.237 | 0.167 | 0.875 | 0.450 | 1.702 | 0.696 |
| **H-Insulin** | 1.268 | 0.649 | 2.479 | 0.488 | 1.477 | 0.626 | 3.487 | 0.374 | 0.940 | 0.304 | 2.906 | 0.915 |
| **H-Glucose** | 1.452 | 0.405 | 5.209 | 0.566 | 2.096 | 0.408 | 10.770 | 0.367 | 0.899 | 0.108 | 7.497 | 0.922 |
| **H-HOMA** | 1.618 | 0.851 | 3.077 | 0.139 | 1.627 | 0.680 | 3.893 | 0.273 | 1.644 | 0.627 | 4.305 | 0.309 |
| **H-Cholesterol** | 1.776 | 0.846 | 3.726 | 0.125 | 2.150 | 0.852 | 5.426 | 0.099 | 1.282 | 0.355 | 4.633 | 0.705 |
| **H-Triglycerides** | 1.009 | 0.567 | 1.794 | 0.976 | 1.144 | 0.539 | 2.427 | 0.727 | 0.837 | 0.333 | 2.107 | 0.707 |
| **L-HDL** | 0.953 | 0.585 | 1.552 | 0.847 | 0.984 | 0.455 | 2.128 | 0.967 | 0.939 | 0.489 | 1.806 | 0.852 |
| **H-LDL** | 0.769 | 0.226 | 2.614 | 0.674 | 1.338 | 0.367 | 4.881 | 0.660 | 0.962 | 0.940 | 0.985 | 0.202 |

**ELOVL3**

| | | | | | | | | | | | | |
|---|---|---|---|---|---|---|---|---|---|---|---|---|
| | | | | | | **rs10748816** | | | | | | |
| | **Total** | | | | **Men** | | | | **Women** | | | |
| **Clinical marker** | **OR** | **95% CI** | | ***p-value*** | **OR** | **95% CI** | | ***p-value*** | **OR** | **95% CI** | | ***p-value*** |
| **H-BMI** | 0.910 | 0.619 | 1.338 | 0.632 | 0.925 | 0.544 | 1.573 | 0.776 | 0.917 | 0.523 | 1.611 | 0.765 |
| **H- Waist** | 1.126 | 0.758 | 1.673 | 0.556 | 1.162 | 0.643 | 2.102 | 0.620 | 1.012 | 0.588 | 1.741 | 0.967 |

| | | | | | | | | | | | | |
|---|---|---|---|---|---|---|---|---|---|---|---|---|
| **H-WHI** | 0.578 | 0.401 | 0.832 | 0.003 | 0.508 | 0.298 | 0.866 | 0.012 | 0.751 | 0.426 | 1.324 | 0.323 |
| **H-WHR** | 0.804 | 0.558 | 1.158 | 0.241 | 0.896 | 0.539 | 1.490 | 0.674 | 0.713 | 0.420 | 1.209 | 0.209 |
| **H-%BF** | 1.018 | 0.704 | 1.471 | 0.926 | 1.059 | 0.634 | 1.771 | 0.827 | 1.014 | 0.595 | 1.729 | 0.958 |
| **H-Insulin** | 1.333 | 0.740 | 2.401 | 0.339 | 1.750 | 0.803 | 3.814 | 0.157 | 1.042 | 0.421 | 2.579 | 0.929 |
| **H-Glucose** | 1.227 | 0.394 | 3.820 | 0.724 | 1.549 | 0.315 | 7.614 | 0.589 | 0.969 | 0.192 | 4.907 | 0.970 |
| **H-HOMA** | 1.230 | 0.691 | 2.191 | 0.482 | 1.313 | 0.625 | 2.756 | 0.474 | 1.265 | 0.496 | 3.224 | 0.624 |
| **H-Cholesterol** | 1.642 | 0.776 | 3.476 | 0.192 | 1.421 | 0.583 | 3.461 | 0.440 | 2.703 | 0.607 | 12.035 | 0.176 |
| **H-Triglycerides** | 0.817 | 0.528 | 1.265 | 0.366 | 0.812 | 0.458 | 1.439 | 0.477 | 0.906 | 0.453 | 1.810 | 0.780 |
| **L-HDL** | 0.825 | 0.569 | 1.196 | 0.311 | 0.746 | 0.422 | 1.318 | 0.313 | 0.766 | 0.456 | 1.286 | 0.315 |
| **H-LDL** | 1.687 | 0.628 | 4.533 | 0.295 | 1.052 | 0.359 | 3.084 | 0.926 | 1.044 | 1.017 | 1.073 | 0.068 |

| | | | | | | | | | | | | |
|---|---|---|---|---|---|---|---|---|---|---|---|---|
| | | | | | | **rs36103207** | | | | | | |
| | **Total** | | | | **Men** | | | | **Women** | | | |
| **Clinical marker** | **OR** | **95% CI** | | ***p-value*** | **OR** | **95% CI** | | ***p-value*** | **OR** | **95% CI** | | ***p-value*** |
| **H-BMI** | 0.397 | 0.086 | 1.829 | 0.221 | 0.459 | 0.051 | 4.165 | 0.480 | 0.356 | 0.042 | 3.002 | 0.324 |
| **H- Waist** | | | | | | | | | | | | |
| **H-WHI** | 0.675 | 0.201 | 2.268 | 0.524 | 0.480 | 0.079 | 2.916 | 0.417 | 1.033 | 0.197 | 5.429 | 0.969 |
| **H-WHR** | 0.947 | 0.297 | 3.018 | 0.926 | 0.326 | 0.036 | 2.952 | 0.296 | 1.795 | 0.395 | 8.163 | 0.444 |
| **H-%BF** | 0.643 | 0.208 | 1.990 | 0.441 | 0.224 | 0.025 | 2.028 | 0.147 | 1.174 | 0.288 | 4.787 | 0.826 |
| **H-Insulin** | 0.814 | 0.778 | 0.853 | 0.179 | 0.765 | 0.708 | 0.827 | 0.341 | 0.858 | 0.813 | 0.906 | 0.367 |
| **H-Glucose** | 0.971 | 0.957 | 0.985 | 0.534 | 0.968 | 0.948 | 0.989 | 0.687 | 0.974 | 0.956 | 0.992 | 0.643 |
| **H-HOMA** | 0.858 | 0.829 | 0.888 | 0.160 | 0.821 | 0.775 | 0.870 | 0.353 | 0.891 | 0.856 | 0.928 | 0.326 |
| **H-Cholesterol** | 0.978 | 0.124 | 7.691 | 0.983 | 2.277 | 0.246 | 21.085 | 0.458 | 0.941 | 0.914 | 0.968 | 0.479 |
| **H-Triglycerides** | 0.320 | 0.041 | 2.487 | 0.252 | 0.746 | 0.082 | 6.789 | 0.795 | 0.835 | 0.794 | 0.878 | 0.211 |
| **L-HDL** | 1.111 | 0.359 | 3.439 | 0.856 | 0.746 | 0.082 | 6.789 | 0.795 | 1.164 | 0.286 | 4.741 | 0.832 |
| **H-LDL** | 1.795 | 0.225 | 14.332 | 0.577 | 4.172 | 0.440 | 39.527 | 0.178 | 0.967 | 0.947 | 0.987 | 0.603 |

**ELOVL4**

| | | | | | | | | | | | | |
|---|---|---|---|---|---|---|---|---|---|---|---|---|
| | | | | | | **rs3812153** | | | | | | |
| | **Total** | | | | **Men** | | | | **Women** | | | |
| **Clinical marker** | **OR** | **95% CI** | | ***p-value*** | **OR** | **95% CI** | | ***p-value*** | **OR** | **95% CI** | | ***p-value*** |

| | | | | | | | | | | | | |
|---|---|---|---|---|---|---|---|---|---|---|---|---|
| **H-BMI** | 0.741 | 0.506 | 1.085 | 0.123 | 0.695 | 0.404 | 1.196 | 0.189 | 0.787 | 0.460 | 1.347 | 0.384 |
| **H- Waist** | 0.862 | 0.589 | 1.261 | 0.444 | 0.830 | 0.459 | 1.499 | 0.537 | 0.885 | 0.534 | 1.467 | 0.636 |
| **H-WHI** | 0.945 | 0.664 | 1.345 | 0.754 | 0.914 | 0.551 | 1.516 | 0.729 | 0.961 | 0.559 | 1.650 | 0.885 |
| **H-WHR** | 0.901 | 0.633 | 1.283 | 0.565 | 0.795 | 0.478 | 1.325 | 0.381 | 1.012 | 0.621 | 1.652 | 0.961 |
| **H-%BF** | 0.908 | 0.636 | 1.295 | 0.594 | 0.932 | 0.560 | 1.553 | 0.789 | 0.882 | 0.537 | 1.450 | 0.623 |
| **H-Insulin** | 0.635 | 0.358 | 1.127 | 0.119 | 0.519 | 0.239 | 1.127 | 0.095 | 0.790 | 0.334 | 1.868 | 0.593 |
| **H-Glucose** | 1.215 | 0.443 | 3.337 | 0.706 | 2.838 | 0.744 | 10.832 | 0.113 | 0.313 | 0.038 | 2.578 | 0.256 |
| **H-HOMA** | 0.633 | 0.360 | 1.111 | 0.109 | 0.567 | 0.266 | 1.212 | 0.141 | 0.724 | 0.311 | 1.686 | 0.455 |
| **H-Cholesterol** | 1.157 | 0.616 | 2.172 | 0.651 | 1.385 | 0.625 | 3.068 | 0.423 | 0.854 | 0.296 | 2.466 | 0.771 |
| **H-Triglycerides** | 0.910 | 0.589 | 1.405 | 0.670 | 0.662 | 0.362 | 1.211 | 0.180 | 1.320 | 0.699 | 2.491 | 0.393 |
| **L-HDL** | 1.099 | 0.768 | 1.574 | 0.605 | 1.129 | 0.639 | 1.995 | 0.678 | 1.098 | 0.678 | 1.779 | 0.704 |
| **H-LDL** | 1.825 | 0.837 | 3.981 | 0.126 | 2.038 | 0.759 | 5.468 | 0.151 | 1.514 | 0.417 | 5.495 | 0.527 |
| | | | | | | | **rs117891930** | | | | | |
| | **Total** | | | | **Men** | | | | **Women** | | | |
| **Clinical marker** | **OR** | **95% CI** | | ***p-value*** | **OR** | **95% CI** | | ***p-value*** | **OR** | **95% CI** | | ***p-value*** |
| **H-BMI** | 1.158 | 0.477 | 2.81 | 0.746 | 0.688 | 0.178 | 2.652 | 0.586 | 1.861 | 0.554 | 6.257 | 0.31 |
| **H- Waist** | 1.575 | 0.661 | 3.752 | 0.303 | 1.147 | 0.296 | 4.446 | 0.843 | 2.131 | 0.635 | 7.147 | 0.212 |
| **H-WHI** | 0.94 | 0.396 | 2.235 | 0.889 | 0.87 | 0.259 | 2.919 | 0.822 | 0.967 | 0.25 | 3.733 | 0.961 |
| **H-WHR** | 1.11 | 0.472 | 2.611 | 0.811 | 0.749 | 0.214 | 2.618 | 0.651 | 1.622 | 0.484 | 5.436 | 0.43 |
| **H-%BF** | 0.684 | 0.275 | 1.699 | 0.411 | 0.252 | 0.051 | 1.233 | 0.068 | 1.422 | 0.424 | 4.765 | 0.568 |
| **H-Insulin** | 1.933 | 0.66 | 5.657 | 0.222 | 0.948 | 0.19 | 4.73 | 0.948 | 4.029 | 0.912 | 17.793 | 0.049 |
| **H-Glucose** | 3.747 | 0.802 | 17.5 | 0.072 | 3.363 | 0.383 | 29.526 | 0.248 | 4.186 | 0.469 | 37.327 | 0.165 |
| **H-HOMA** | 3.582 | 1.381 | 9.29 | 0.005 | 3.317 | 0.896 | 12.28 | 0.059 | 3.903 | 0.955 | 15.95 | 0.042 |
| **H-Cholesterol** | 1.182 | 0.268 | 5.22 | 0.825 | 0.889 | 0.11 | 7.207 | 0.913 | 1.665 | 0.201 | 13.773 | 0.634 |
| **H-Triglycerides** | 1.49 | 0.571 | 3.893 | 0.413 | 1.756 | 0.499 | 6.183 | 0.377 | 1.167 | 0.244 | 5.568 | 0.847 |
| **L-HDL** | 0.655 | 0.253 | 1.7 | 0.383 | 0.657 | 0.139 | 3.115 | 0.596 | 0.653 | 0.187 | 2.278 | 0.502 |
| **H-LDL** | 1.009 | 0.131 | 7.794 | 0.993 | 0.939 | 0.911 | 0.967 | 0.399 | 3.233 | 0.373 | 28.037 | 0.262 |
| | | | | | | | **rs80246554** | | | | | |
| | **Total** | | | | **Men** | | | | **Women** | | | |

| Clinical marker | OR | 95% CI | | *p-value* | OR | 95% CI | | *p-value* | OR | 95% CI | | *p-value* |
|---|---|---|---|---|---|---|---|---|---|---|---|---|
| **H-BMI** | 0.965 | 0.474 | 1.965 | 0.923 | 0.599 | 0.211 | 1.701 | 0.333 | 1.567 | 0.578 | 4.251 | 0.376 |
| **H- Waist** | 1.077 | 0.529 | 2.194 | 0.838 | 0.746 | 0.241 | 2.31 | 0.612 | 1.575 | 0.59 | 4.204 | 0.363 |
| **H-WHI** | 1.312 | 0.674 | 2.554 | 0.424 | 0.883 | 0.354 | 2.203 | 0.791 | 1.878 | 0.691 | 5.105 | 0.211 |
| **H-WHR** | 1.012 | 0.517 | 1.98 | 0.973 | 0.875 | 0.346 | 2.212 | 0.779 | 1.192 | 0.447 | 3.179 | 0.726 |
| **H-%BF** | 0.713 | 0.353 | 1.441 | 0.345 | 0.478 | 0.172 | 1.332 | 0.151 | 1.042 | 0.391 | 2.779 | 0.935 |
| **H-Insulin** | 2.244 | 0.931 | 5.407 | 0.066 | 1.362 | 0.406 | 4.565 | 0.618 | 3.937 | 1.08 | 14.345 | 0.027 |
| **H-Glucose** | 2.084 | 0.458 | 9.475 | 0.332 | 1.711 | 0.203 | 14.4 | 0.619 | 2.563 | 0.297 | 22.107 | 0.377 |
| **H-HOMA** | 2.622 | 1.164 | 5.908 | 0.016 | 2.261 | 0.745 | 6.864 | 0.142 | 3.086 | 0.93 | 10.239 | 0.054 |
| **H-Cholesterol** | 1.039 | 0.307 | 3.518 | 0.951 | 0.451 | 0.058 | 3.499 | 0.437 | 2.317 | 0.487 | 11.014 | 0.279 |
| **H-Triglycerides** | 1.488 | 0.7 | 3.164 | 0.3 | 1.682 | 0.644 | 4.394 | 0.286 | 1.126 | 0.311 | 4.072 | 0.857 |
| **L-HDL** | 0.842 | 0.414 | 1.712 | 0.636 | 0.735 | 0.238 | 2.275 | 0.594 | 1.033 | 0.388 | 2.75 | 0.949 |
| **H-LDL** | 1.227 | 0.279 | 5.394 | 0.786 | 0.937 | 0.908 | 0.966 | 0.247 | 4.767 | 0.93 | 24.43 | 0.04 |

| | | | | | | | **rs12196014** | | | | | |
|---|---|---|---|---|---|---|---|---|---|---|---|---|
| | | **Total** | | | | **Men** | | | | **Women** | | |
| **Clinical marker** | **OR** | **95% CI** | | ***p-value*** | **OR** | **95% CI** | | ***p-value*** | **OR** | **95% CI** | | ***p-value*** |
| **H-BMI** | 0.93 | 0.571 | 1.515 | 0.772 | 0.793 | 0.4 | 1.573 | 0.508 | 1.091 | 0.544 | 2.188 | 0.807 |
| **H- Waist** | 0.882 | 0.538 | 1.448 | 0.621 | 0.917 | 0.438 | 1.919 | 0.818 | 0.895 | 0.455 | 1.761 | 0.749 |
| **H-WHI** | 1.088 | 0.691 | 1.712 | 0.717 | 0.982 | 0.52 | 1.853 | 0.955 | 1.099 | 0.542 | 2.226 | 0.795 |
| **H-WHR** | 0.991 | 0.626 | 1.569 | 0.97 | 1.134 | 0.6 | 2.145 | 0.699 | 0.859 | 0.442 | 1.671 | 0.656 |
| **H-%BF** | 0.769 | 0.48 | 1.232 | 0.275 | 0.628 | 0.325 | 1.217 | 0.167 | 0.935 | 0.477 | 1.832 | 0.846 |
| **H-Insulin** | 1.519 | 0.797 | 2.896 | 0.202 | 1.495 | 0.652 | 3.428 | 0.343 | 1.389 | 0.484 | 3.988 | 0.542 |
| **H-Glucose** | 1.153 | 0.324 | 4.095 | 0.826 | 0.601 | 0.073 | 4.919 | 0.633 | 2.008 | 0.392 | 10.291 | 0.396 |
| **H-HOMA** | 1.591 | 0.85 | 2.98 | 0.144 | 1.787 | 0.795 | 4.015 | 0.157 | 1.286 | 0.46 | 3.593 | 0.632 |
| **H-Cholesterol** | 0.811 | 0.333 | 1.977 | 0.646 | 0.582 | 0.168 | 2.02 | 0.391 | 1.19 | 0.33 | 4.297 | 0.791 |
| **H-Triglycerides** | 1.562 | 0.929 | 2.628 | 0.091 | 1.978 | 1.011 | 3.871 | 0.044 | 1.035 | 0.431 | 2.489 | 0.938 |
| **L-HDL** | 1.063 | 0.666 | 1.696 | 0.8 | 0.944 | 0.451 | 1.977 | 0.879 | 1.277 | 0.669 | 2.44 | 0.459 |
| **H-LDL** | 0.415 | 0.097 | 1.786 | 0.224 | 0.926 | 0.892 | 0.96 | 0.054 | 1.494 | 0.306 | 7.285 | 0.619 |

| | | | | | | | **rs9448863** | | | | | |
|---|---|---|---|---|---|---|---|---|---|---|---|---|

| | Total | | | | Men | | | | Women | | | |
|---|---|---|---|---|---|---|---|---|---|---|---|---|
| **Clinical marker** | **OR** | **95% CI** | | ***p-value*** | **OR** | **95% CI** | | ***p-value*** | **OR** | **95% CI** | | ***p-value*** |
| **H-BMI** | 0.761 | 0.52 | 1.112 | 0.158 | 0.712 | 0.414 | 1.226 | 0.221 | 0.812 | 0.477 | 1.382 | 0.443 |
| **H- Waist** | 0.885 | 0.605 | 1.293 | 0.527 | 0.848 | 0.469 | 1.532 | 0.586 | 0.902 | 0.546 | 1.489 | 0.687 |
| **H-WHI** | 0.903 | 0.634 | 1.284 | 0.57 | 0.888 | 0.535 | 1.474 | 0.648 | 0.922 | 0.537 | 1.581 | 0.768 |
| **H-WHR** | 0.918 | 0.646 | 1.306 | 0.636 | 0.818 | 0.491 | 1.364 | 0.442 | 1.021 | 0.628 | 1.661 | 0.933 |
| **H-%BF** | 0.923 | 0.648 | 1.316 | 0.66 | 0.966 | 0.579 | 1.612 | 0.895 | 0.888 | 0.542 | 1.455 | 0.639 |
| **H-Insulin** | 0.574 | 0.32 | 1.029 | 0.06 | 0.454 | 0.204 | 1.012 | 0.05 | 0.753 | 0.319 | 1.778 | 0.518 |
| **H-Glucose** | 1.205 | 0.439 | 3.31 | 0.717 | 2.887 | 0.756 | 11.019 | 0.106 | 0.303 | 0.037 | 2.5 | 0.242 |
| **H-HOMA** | 0.576 | 0.325 | 1.022 | 0.057 | 0.496 | 0.226 | 1.088 | 0.077 | 0.7 | 0.301 | 1.628 | 0.407 |
| **H-Cholesterol** | 1.147 | 0.611 | 2.153 | 0.67 | 1.41 | 0.636 | 3.125 | 0.397 | 0.827 | 0.286 | 2.388 | 0.726 |
| **H-Triglycerides** | 0.901 | 0.584 | 1.391 | 0.639 | 0.676 | 0.369 | 1.237 | 0.204 | 1.273 | 0.675 | 2.401 | 0.457 |
| **L-HDL** | 1.05 | 0.733 | 1.504 | 0.79 | 1.06 | 0.597 | 1.881 | 0.844 | 1.038 | 0.643 | 1.677 | 0.879 |
| **H-LDL** | 1.81 | 0.83 | 3.948 | 0.131 | 2.074 | 0.773 | 5.567 | 0.141 | 1.468 | 0.405 | 5.325 | 0.558 |

**rs16891339**

| | Total | | | | Men | | | | Women | | | |
|---|---|---|---|---|---|---|---|---|---|---|---|---|
| **Clinical marker** | **OR** | **95% CI** | | ***p-value*** | **OR** | **95% CI** | | ***p-value*** | **OR** | **95% CI** | | ***p-value*** |
| **H-BMI** | 0.367 | 0.106 | 1.276 | 0.101 | 0.197 | 0.025 | 1.58 | 0.09 | 0.614 | 0.125 | 3.015 | 0.546 |
| **H- Waist** | 0.585 | 0.192 | 1.788 | 0.343 | 0.743 | 0.693 | 0.796 | 0.065 | 1.394 | 0.367 | 5.303 | 0.625 |
| **H-WHI** | 1.23 | 0.492 | 3.072 | 0.658 | 0.714 | 0.202 | 2.524 | 0.601 | 2.117 | 0.555 | 8.078 | 0.264 |
| **H-WHR** | 0.961 | 0.381 | 2.425 | 0.933 | 0.554 | 0.14 | 2.186 | 0.394 | 1.687 | 0.444 | 6.408 | 0.44 |
| **H-%BF** | 0.467 | 0.175 | 1.245 | 0.12 | 0.377 | 0.095 | 1.489 | 0.15 | 0.575 | 0.141 | 2.345 | 0.437 |
| **H-Insulin** | 0.893 | 0.192 | 4.162 | 0.886 | 0.541 | 0.063 | 4.611 | 0.571 | 1.575 | 0.17 | 14.573 | 0.688 |
| **H-Glucose** | 0.971 | 0.957 | 0.984 | 0.449 | 0.968 | 0.947 | 0.989 | 0.564 | 0.974 | 0.956 | 0.992 | 0.622 |
| **H-HOMA** | 0.882 | 0.196 | 3.961 | 0.87 | 0.571 | 0.07 | 4.683 | 0.599 | 1.422 | 0.166 | 12.216 | 0.748 |
| **H-Cholesterol** | 0.919 | 0.897 | 0.941 | 0.196 | 0.895 | 0.86 | 0.932 | 0.282 | 0.94 | 0.914 | 0.967 | 0.452 |
| **H-Triglycerides** | 0.725 | 0.208 | 2.528 | 0.613 | 0.322 | 0.04 | 2.59 | 0.264 | 1.512 | 0.305 | 7.499 | 0.612 |
| **L-HDL** | 0.811 | 0.304 | 2.165 | 0.676 | 0.322 | 0.04 | 2.59 | 0.264 | 1.466 | 0.386 | 5.565 | 0.574 |
| **H-LDL** | 0.953 | 0.936 | 0.971 | 0.336 | 0.939 | 0.911 | 0.967 | 0.421 | 0.967 | 0.947 | 0.987 | 0.58 |

**ELOVL5**

| | | | | | | | | | | | | |
|---|---|---|---|---|---|---|---|---|---|---|---|---|
| | **rs41273878** | | | | | | | | | | | |
| | **Total** | | | | **Men** | | | | **Women** | | | |
| **Clinical marker** | **OR** | **95% CI** | | ***p*-value** | **OR** | **95% CI** | | ***p*-value** | **OR** | **95% CI** | | ***p*-value** |
| **H-BMI** | 0.894 | 0.272 | 2.939 | 0.853 | 0.738 | 0.141 | 3.876 | 0.72 | 1.091 | 0.196 | 6.065 | 0.921 |
| **H- Waist** | 0.665 | 0.181 | 2.444 | 0.537 | 0.5 | 0.059 | 4.225 | 0.518 | 0.86 | 0.155 | 4.773 | 0.864 |
| **H-WHI** | 0.848 | 0.274 | 2.622 | 0.774 | 0.971 | 0.213 | 4.421 | 0.97 | 0.511 | 0.059 | 4.435 | 0.536 |
| **H-WHR** | 0.826 | 0.267 | 2.555 | 0.74 | 0.522 | 0.1 | 2.738 | 0.437 | 1.336 | 0.265 | 6.728 | 0.726 |
| **H-%BF** | 0.888 | 0.295 | 2.677 | 0.834 | 0.358 | 0.068 | 1.876 | 0.207 | 2.379 | 0.429 | 13.193 | 0.309 |
| **H-Insulin** | 0.815 | 0.778 | 0.853 | 0.209 | 0.764 | 0.707 | 0.826 | 0.27 | 0.86 | 0.815 | 0.907 | 0.487 |
| **H-Glucose** | 0.971 | 0.957 | 0.985 | 0.534 | 0.968 | 0.948 | 0.989 | 0.632 | 0.974 | 0.956 | 0.992 | 0.689 |
| **H-HOMA** | 0.858 | 0.829 | 0.888 | 0.16 | 0.819 | 0.772 | 0.868 | 0.216 | 0.892 | 0.857 | 0.929 | 0.44 |
| **H-Cholesterol** | 0.978 | 0.124 | 7.691 | 0.983 | 1.506 | 0.175 | 12.963 | 0.709 | 0.941 | 0.915 | 0.968 | 0.541 |
| **H-Triglycerides** | 2.505 | 0.805 | 7.799 | 0.102 | 4.176 | 0.912 | 19.127 | 0.047 | 1.045 | 0.119 | 9.139 | 0.968 |
| **L-HDL** | 1.111 | 0.359 | 3.439 | 0.856 | 0.493 | 0.058 | 4.165 | 0.509 | 2.357 | 0.425 | 13.062 | 0.314 |
| **H-LDL** | 1.795 | 0.225 | 14.332 | 0.577 | 2.76 | 0.313 | 24.329 | 0.342 | 0.967 | 0.947 | 0.987 | 0.653 |
| | **rs72938776** | | | | | | | | | | | |
| | **Total** | | | | **Men** | | | | **Women** | | | |
| **Clinical marker** | **OR** | **95% CI** | | ***p*-value** | **OR** | **95% CI** | | ***p*-value** | **OR** | **95% CI** | | ***p*-value** |
| **H-BMI** | 12.447 | 1.488 | 104.127 | 0.003 | 0.343 | 0.292 | 0.403 | 0.018 | 6.717 | 0.69 | 65.443 | 0.059 |
| **H- Waist** | 3.022 | 0.67 | 13.642 | 0.132 | 6.232 | 0.557 | 69.784 | 0.091 | 1.739 | 0.242 | 12.516 | 0.58 |
| **H-WHI** | 1.021 | 0.227 | 4.603 | 0.978 | 1.463 | 0.131 | 16.326 | 0.757 | 0.859 | 0.088 | 8.371 | 0.896 |
| **H-WHR** | 8.129 | 0.972 | 67.951 | 0.022 | 2.678 | 0.24 | 29.872 | 0.407 | 0.422 | 0.37 | 0.482 | 0.02 |
| **H-%BF** | 6.35 | 0.76 | 53.088 | 0.051 | 1.845 | 0.165 | 20.588 | 0.615 | 0.454 | 0.4 | 0.515 | 0.029 |
| **H-Insulin** | 6.973 | 1.145 | 42.469 | 0.015 | 0.223 | 0.172 | 0.29 | 0.009 | 3.183 | 0.28 | 36.2 | 0.327 |
| **H-Glucose** | 0.971 | 0.958 | 0.985 | 0.65 | 0.968 | 0.948 | 0.989 | 0.756 | 0.974 | 0.956 | 0.992 | 0.745 |
| **H-HOMA** | 1.245 | 0.143 | 10.807 | 0.842 | 2.364 | 0.21 | 26.645 | 0.475 | 0.893 | 0.858 | 0.929 | 0.551 |
| **H-Cholesterol** | 1.978 | 0.233 | 16.785 | 0.525 | 0.898 | 0.864 | 0.934 | 0.562 | 5.686 | 0.561 | 57.597 | 0.098 |
| **H-Triglycerides** | 1.573 | 0.302 | 8.209 | 0.588 | 1.507 | 0.135 | 16.871 | 0.739 | 1.755 | 0.179 | 17.222 | 0.626 |

| | | | | | | | | | | | | |
|---|---|---|---|---|---|---|---|---|---|---|---|---|
| **L-HDL** | 2.39 | 0.53 | 10.779 | 0.243 | 6.143 | 0.549 | 68.776 | 0.094 | 1.162 | 0.162 | 8.355 | 0.882 |
| **H-LDL** | 3.628 | 0.421 | 31.247 | 0.21 | 0.94 | 0.913 | 0.968 | 0.664 | 11.037 | 1.044 | 116.696 | 0.013 |
| | | | | | | **rs36054518** | | | | | | |
| | **Total** | | | | **Men** | | | | **Women** | | | |
| **Clinical marker** | **OR** | **95% CI** | | ***p*-*value*** | **OR** | **95% CI** | | ***p*-*value*** | **OR** | **95% CI** | | ***p*-*value*** |
| **H-BMI** | 0.767 | 0.27 | 2.184 | 0.619 | 0.521 | 0.106 | 2.559 | 0.416 | 1.087 | 0.266 | 4.442 | 0.908 |
| **H- Waist** | 0.629 | 0.204 | 1.936 | 0.416 | 0.745 | 0.695 | 0.798 | 0.081 | 1.387 | 0.365 | 5.275 | 0.631 |
| **H-WHI** | 0.86 | 0.329 | 2.25 | 0.758 | 0.573 | 0.151 | 2.179 | 0.41 | 1.295 | 0.317 | 5.299 | 0.719 |
| **H-WHR** | 0.837 | 0.32 | 2.191 | 0.718 | 0.368 | 0.075 | 1.805 | 0.202 | 1.677 | 0.441 | 6.37 | 0.445 |
| **H-%BF** | 1.044 | 0.408 | 2.671 | 0.928 | 0.447 | 0.109 | 1.824 | 0.252 | 2.419 | 0.593 | 9.861 | 0.206 |
| **H-Insulin** | 2.308 | 0.677 | 7.87 | 0.171 | 3.432 | 0.47 | 25.068 | 0.199 | 2.138 | 0.412 | 11.104 | 0.358 |
| **H-Glucose** | 0.971 | 0.957 | 0.985 | 0.462 | 0.968 | 0.947 | 0.989 | 0.586 | 0.973 | 0.955 | 0.992 | 0.622 |
| **H-HOMA** | 1.249 | 0.353 | 4.422 | 0.73 | 0.574 | 0.07 | 4.706 | 0.602 | 2.502 | 0.496 | 12.617 | 0.252 |
| **H-Cholesterol** | 0.699 | 0.091 | 5.376 | 0.73 | 0.896 | 0.861 | 0.933 | 0.309 | 2.227 | 0.262 | 18.905 | 0.453 |
| **H-Triglycerides** | 0.223 | 0.029 | 1.693 | 0.113 | 0.742 | 0.692 | 0.795 | 0.079 | 0.643 | 0.079 | 5.256 | 0.679 |
| **L-HDL** | 1.138 | 0.435 | 2.98 | 0.793 | 0.853 | 0.173 | 4.203 | 0.846 | 1.476 | 0.389 | 5.606 | 0.566 |
| **H-LDL** | 2.896 | 0.63 | 13.316 | 0.0154 | 0.939 | 0.911 | 0.968 | 0.447 | 12 | 2.104 | 68.442 | 0 |
| | | | | | | **rs72940713** | | | | | | |
| | **Total** | | | | **Men** | | | | **Women** | | | |
| **Clinical marker** | **OR** | **95% CI** | | ***p*-*value*** | **OR** | **95% CI** | | ***p*-*value*** | **OR** | **95% CI** | | ***p*-*value*** |
| **H-BMI** | 7.32 | 1.506 | 35.579 | 0.004 | 0.341 | 0.289 | 0.401 | 0.006 | 3.379 | 0.555 | 20.569 | 0.163 |
| **H- Waist** | 2.835 | 0.752 | 10.682 | 0.108 | 3.101 | 0.429 | 22.433 | 0.24 | 2.618 | 0.431 | 15.91 | 0.28 |
| **H-WHI** | 1.087 | 0.289 | 4.09 | 0.902 | 2.209 | 0.227 | 21.494 | 0.486 | 0.638 | 0.07 | 5.792 | 0.689 |
| **H-WHR** | 4.766 | 0.982 | 23.141 | 0.033 | 4.05 | 0.416 | 39.416 | 0.194 | 5.492 | 0.607 | 49.73 | 0.09 |
| **H-%BF** | 8.561 | 1.064 | 68.899 | 0.016 | 2.787 | 0.286 | 27.131 | 0.359 | 0.45 | 0.396 | 0.511 | 0.014 |
| **H-Insulin** | 6.953 | 1.142 | 42.346 | 0.015 | 0.223 | 0.172 | 0.29 | 0.009 | 3.167 | 0.278 | 36.011 | 0.33 |
| **H-Glucose** | 0.971 | 0.958 | 0.985 | 0.606 | 0.968 | 0.948 | 0.989 | 0.719 | 0.974 | 0.956 | 0.992 | 0.715 |
| **H-HOMA** | 1.243 | 0.143 | 10.784 | 0.844 | 2.364 | 0.21 | 26.645 | 0.475 | 0.893 | 0.858 | 0.929 | 0.55 |
| **H-Cholesterol** | 1.476 | 0.181 | 12.056 | 0.715 | 0.898 | 0.863 | 0.934 | 0.502 | 4.235 | 0.449 | 39.993 | 0.172 |

| | | | | | | | | | | | | |
|---|---|---|---|---|---|---|---|---|---|---|---|---|
| **H-Triglycerides** | 1.975 | 0.487 | 8.012 | 0.333 | 1 | 0.102 | 9.767 | 1 | 3.569 | 0.581 | 21.932 | 0.144 |
| **L-HDL** | 1.433 | 0.381 | 5.395 | 0.593 | 3.057 | 0.423 | 22.108 | 0.246 | 0.775 | 0.128 | 4.706 | 0.782 |
| **H-LDL** | 0.956 | 0.939 | 0.973 | 0.52 | 0.94 | 0.913 | 0.968 | 0.615 | 0.97 | 0.952 | 0.99 | 0.698 |
| | | | | | | | **rs114271869** | | | | | |
| | | **Total** | | | | **Men** | | | | **Women** | | |
| **Clinical marker** | **OR** | **95% CI** | | ***p-value*** | **OR** | **95% CI** | | ***p-value*** | **OR** | **95% CI** | | ***p-value*** |
| **H-BMI** | 0.896 | 0.272 | 2.947 | 0.857 | 1.113 | 0.26 | 4.756 | 0.886 | 0.546 | 0.06 | 4.95 | 0.586 |
| **H- Waist** | 0.996 | 0.303 | 3.276 | 0.994 | 1.01 | 0.199 | 5.119 | 0.991 | 1.164 | 0.191 | 7.071 | 0.87 |
| **H-WHI** | 1.172 | 0.389 | 3.531 | 0.778 | 0.727 | 0.178 | 2.965 | 0.656 | 1.73 | 0.284 | 10.538 | 0.549 |
| **H-WHR** | 0.832 | 0.269 | 2.576 | 0.751 | 0.797 | 0.187 | 3.4 | 0.759 | 0.891 | 0.147 | 5.408 | 0.9 |
| **H-%BF** | 0.648 | 0.209 | 2.005 | 0.449 | 0.544 | 0.127 | 2.324 | 0.407 | 0.782 | 0.129 | 4.75 | 0.79 |
| **H-Insulin** | 0.644 | 0.078 | 5.311 | 0.681 | 0.653 | 0.074 | 5.738 | 0.701 | 0.864 | 0.82 | 0.911 | 0.577 |
| **H-Glucose** | 0.971 | 0.957 | 0.985 | 0.533 | 0.968 | 0.947 | 0.989 | 0.607 | 0.974 | 0.956 | 0.992 | 0.715 |
| **H-HOMA** | 0.86 | 0.831 | 0.89 | 0.163 | 0.818 | 0.771 | 0.867 | 0.215 | 0.895 | 0.861 | 0.932 | 0.447 |
| **H-Cholesterol** | 0.92 | 0.898 | 0.942 | 0.287 | 0.896 | 0.861 | 0.933 | 0.338 | 0.941 | 0.915 | 0.968 | 0.577 |
| **H-Triglycerides** | 1.172 | 0.318 | 4.327 | 0.812 | 0.995 | 0.196 | 5.044 | 0.995 | 1.306 | 0.143 | 11.936 | 0.813 |
| **L-HDL** | 2.914 | 0.941 | 9.022 | 0.053 | 5.274 | 1.228 | 22.651 | 0.013 | 1.768 | 0.291 | 10.73 | 0.532 |
| **H-LDL** | 0.954 | 0.937 | 0.971 | 0.428 | 0.939 | 0.911 | 0.968 | 0.473 | 0.967 | 0.947 | 0.987 | 0.682 |
| | | | | | | | **rs 2073040** | | | | | |
| | | **Total** | | | | **Men** | | | | **Women** | | |
| **Clinical marker** | **OR** | **95% CI** | | ***p-value*** | **OR** | **95% CI** | | ***p-value*** | **OR** | **95% CI** | | ***p-value*** |
| **H-BMI** | 0.96 | 0.654 | 1.409 | 0.837 | 1.269 | 0.729 | 2.208 | 0.402 | 0.734 | 0.43 | 1.252 | 0.257 |
| **H- Waist** | 0.846 | 0.577 | 1.24 | 0.393 | 0.918 | 0.508 | 1.659 | 0.778 | 0.798 | 0.478 | 1.332 | 0.389 |
| **H-WHI** | 0.88 | 0.612 | 1.264 | 0.489 | 0.955 | 0.565 | 1.615 | 0.865 | 0.767 | 0.445 | 1.321 | 0.339 |
| **H-WHR** | 0.886 | 0.616 | 1.274 | 0.513 | 1.066 | 0.632 | 1.798 | 0.813 | 0.745 | 0.449 | 1.235 | 0.254 |
| **H-%BF** | 0.886 | 0.612 | 1.281 | 0.52 | 1.021 | 0.603 | 1.729 | 0.937 | 0.773 | 0.46 | 1.298 | 0.331 |
| **H-Insulin** | 1.275 | 0.722 | 2.252 | 0.403 | 0.869 | 0.415 | 1.821 | 0.712 | 2.017 | 0.786 | 5.174 | 0.139 |
| **H-Glucose** | 1.306 | 0.42 | 4.066 | 0.644 | 0.782 | 0.191 | 3.204 | 0.733 | 2.888 | 0.35 | 23.835 | 0.305 |
| **H-HOMA** | 1.101 | 0.636 | 1.908 | 0.732 | 1.162 | 0.551 | 2.45 | 0.695 | 0.997 | 0.438 | 2.268 | 0.994 |

| | | | | | | | | | | | | |
|---|---|---|---|---|---|---|---|---|---|---|---|---|
| **H-Cholesterol** | 1.754 | 0.828 | 3.713 | 0.138 | 1.576 | 0.617 | 4.028 | 0.34 | 2.096 | 0.591 | 7.432 | 0.244 |
| **H-Triglycerides** | 0.799 | 0.519 | 1.232 | 0.311 | 0.789 | 0.441 | 1.41 | 0.424 | 0.802 | 0.415 | 1.548 | 0.512 |
| **L-HDL** | 0.686 | 0.475 | 0.992 | 0.045 | 0.609 | 0.344 | 1.078 | 0.088 | 0.734 | 0.444 | 1.212 | 0.227 |
| **H-LDL** | 1.087 | 0.448 | 2.636 | 0.854 | 1.3 | 0.411 | 4.113 | 0.656 | 0.802 | 0.196 | 3.282 | 0.759 |

| | | | | | | | | | | | | |
|---|---|---|---|---|---|---|---|---|---|---|---|---|
| | **rs 9370194** | | | | | | | | | | | |
| | **Total** | | | | **Men** | | | | **Women** | | | |
| **Clinical marker** | **OR** | **95% CI** | | ***p-value*** | **OR** | **95% CI** | | ***p-value*** | **OR** | **95% CI** | | ***p-value*** |
| **H-BMI** | 0.934 | 0.631 | 1.383 | 0.735 | 0.72 | 0.417 | 1.244 | 0.24 | 1.217 | 0.691 | 2.142 | 0.498 |
| **H- Waist** | 0.751 | 0.501 | 1.127 | 0.166 | 0.741 | 0.402 | 1.364 | 0.337 | 0.824 | 0.473 | 1.433 | 0.494 |
| **H-WHI** | 0.913 | 0.631 | 1.321 | 0.629 | 0.986 | 0.591 | 1.646 | 0.958 | 0.622 | 0.334 | 1.161 | 0.135 |
| **H-WHR** | 0.794 | 0.547 | 1.152 | 0.225 | 0.731 | 0.435 | 1.227 | 0.236 | 0.873 | 0.509 | 1.496 | 0.622 |
| **H-%BF** | 0.79 | 0.543 | 1.15 | 0.219 | 0.537 | 0.319 | 0.907 | 0.019 | 1.163 | 0.673 | 2.01 | 0.589 |
| **H-Insulin** | 1.463 | 0.848 | 2.524 | 0.17 | 1.586 | 0.778 | 3.234 | 0.205 | 1.193 | 0.497 | 2.86 | 0.695 |
| **H-Glucose** | 0.788 | 0.253 | 2.453 | 0.681 | 1.094 | 0.267 | 4.479 | 0.901 | 0.429 | 0.052 | 3.543 | 0.42 |
| **H-HOMA** | 1.126 | 0.656 | 1.931 | 0.667 | 0.834 | 0.402 | 1.727 | 0.626 | 1.533 | 0.684 | 3.439 | 0.298 |
| **H-Cholesterol** | 1.017 | 0.521 | 1.986 | 0.961 | 0.422 | 0.155 | 1.145 | 0.083 | 2.926 | 1.085 | 7.892 | 0.027 |
| **H-Triglycerides** | 1.078 | 0.692 | 1.681 | 0.739 | 0.89 | 0.495 | 1.6 | 0.699 | 1.275 | 0.642 | 2.529 | 0.489 |
| **L-HDL** | 1.16 | 0.797 | 1.687 | 0.439 | 1.259 | 0.713 | 2.226 | 0.429 | 1.288 | 0.759 | 2.187 | 0.35 |
| **H-LDL** | 0.898 | 0.372 | 2.166 | 0.811 | 0.274 | 0.061 | 1.227 | 0.072 | 3.206 | 0.901 | 11.406 | 0.059 |

**ELOVL6**

| | | | | | | | | | | | | |
|---|---|---|---|---|---|---|---|---|---|---|---|---|
| | **rs11098065** | | | | | | | | | | | |
| | **Total** | | | | **Men** | | | | **Women** | | | |
| **Clinical marker** | **OR** | **95% CI** | | ***p-value*** | **OR** | **95% CI** | | ***p-value*** | **OR** | **95% CI** | | ***p-value*** |
| **H-BMI** | 0.903 | 0.636 | 1.282 | 0.57 | 1.051 | 0.64 | 1.726 | 0.844 | 0.778 | 0.473 | 1.279 | 0.323 |
| **H- Waist** | 1.047 | 0.737 | 1.489 | 0.798 | 1.088 | 0.633 | 1.872 | 0.76 | 1.018 | 0.637 | 1.627 | 0.942 |
| **H-WHI** | 1.158 | 0.833 | 1.61 | 0.384 | 1.094 | 0.679 | 1.763 | 0.712 | 1.275 | 0.773 | 2.106 | 0.343 |
| **H-WHR** | 1.06 | 0.762 | 1.475 | 0.73 | 1.126 | 0.701 | 1.811 | 0.624 | 1.001 | 0.632 | 1.585 | 0.996 |
| **H-%BF** | 0.901 | 0.646 | 1.257 | 0.541 | 1.244 | 0.768 | 2.017 | 0.376 | 0.672 | 0.422 | 1.071 | 0.095 |
| **H-Insulin** | 0.499 | 0.289 | 0.861 | 0.011 | 0.436 | 0.21 | 0.906 | 0.024 | 0.578 | 0.253 | 1.321 | 0.191 |

| | | | | | | | | | | | | |
|---|---|---|---|---|---|---|---|---|---|---|---|---|
| **H-Glucose** | 0.558 | 0.194 | 1.604 | 0.273 | 0.674 | 0.165 | 2.749 | 0.581 | 0.444 | 0.088 | 2.233 | 0.313 |
| **H-HOMA** | 0.725 | 0.438 | 1.2 | 0.21 | 0.656 | 0.333 | 1.291 | 0.222 | 0.833 | 0.388 | 1.786 | 0.639 |
| **H-Cholesterol** | 1.215 | 0.669 | 2.209 | 0.523 | 1.782 | 0.823 | 3.859 | 0.14 | 0.663 | 0.242 | 1.814 | 0.422 |
| **H-Triglycerides** | 0.93 | 0.621 | 1.394 | 0.727 | 1.124 | 0.656 | 1.925 | 0.673 | 0.725 | 0.387 | 1.358 | 0.316 |
| **L-HDL** | 0.686 | 0.487 | 0.966 | 0.031 | 0.828 | 0.48 | 1.429 | 0.499 | 0.582 | 0.369 | 0.919 | 0.02 |
| **H-LDL** | 1.092 | 0.502 | 2.375 | 0.824 | 1.227 | 0.459 | 3.278 | 0.684 | 0.901 | 0.249 | 3.259 | 0.874 |
| | | | | | | **rs17041284** | | | | | | |
| | | **Total** | | | | **Men** | | | | **Women** | | |
| **Clinical marker** | **OR** | **95% CI** | | ***p-value*** | **OR** | **95% CI** | | ***p-value*** | **OR** | **95% CI** | | ***p-value*** |
| **H-BMI** | 1.494 | 0.591 | 3.777 | 0.394 | 1.578 | 0.469 | 5.307 | 0.459 | 1.332 | 0.312 | 5.693 | 0.699 |
| **H- Waist** | 1.52 | 0.611 | 3.784 | 0.366 | 1.783 | 0.506 | 6.277 | 0.364 | 1.407 | 0.37 | 5.353 | 0.616 |
| **H-WHI** | 1.382 | 0.566 | 3.372 | 0.477 | 0.87 | 0.259 | 2.919 | 0.822 | 2.143 | 0.562 | 8.179 | 0.256 |
| **H-WHR** | 1.206 | 0.483 | 3.014 | 0.688 | 1.109 | 0.33 | 3.72 | 0.868 | 1.349 | 0.331 | 5.498 | 0.676 |
| **H-%BF** | 0.937 | 0.375 | 2.341 | 0.889 | 0.755 | 0.225 | 2.536 | 0.65 | 1.183 | 0.29 | 4.824 | 0.815 |
| **H-Insulin** | 1.144 | 0.315 | 4.156 | 0.839 | 0.824 | 0.169 | 4.023 | 0.812 | 1.629 | 0.176 | 15.09 | 0.666 |
| **H-Glucose** | 1.849 | 0.233 | 14.671 | 0.056 | 3.363 | 0.383 | 29.526 | 0.248 | 0.973 | 0.955 | 0.992 | 0.622 |
| **H-HOMA** | 0.781 | 0.176 | 3.467 | 0.745 | 0.455 | 0.057 | 3.643 | 0.449 | 1.471 | 0.171 | 12.65 | 0.725 |
| **H-Cholesterol** | 1.316 | 0.296 | 5.852 | 0.718 | 2.058 | 0.423 | 10.017 | 0.364 | 0.94 | 0.914 | 0.967 | 0.451 |
| **H-Triglycerides** | 2.192 | 0.855 | 5.618 | 0.095 | 2.612 | 0.772 | 8.832 | 0.111 | 1.544 | 0.311 | 7.663 | 0.594 |
| **L-HDL** | 1.195 | 0.481 | 2.97 | 0.702 | 0.657 | 0.139 | 3.115 | 0.596 | 2.394 | 0.588 | 9.751 | 0.211 |
| **H-LDL** | 1.117 | 0.144 | 8.671 | 0.916 | 1.631 | 0.196 | 13.545 | 0.649 | 0.967 | 0.947 | 0.987 | 0.58 |
| | | | | | | **rs7662161** | | | | | | |
| | | **Total** | | | | **Men** | | | | **Women** | | |
| **Clinical marker** | **OR** | **95% CI** | | ***p-value*** | **OR** | **95% CI** | | ***p-value*** | **OR** | **95% CI** | | ***p-value*** |
| **H-BMI** | 0.903 | 0.636 | 1.283 | 0.569 | 0.875 | 0.532 | 1.44 | 0.601 | 0.93 | 0.567 | 1.526 | 0.776 |
| **H- Waist** | 0.946 | 0.664 | 1.348 | 0.758 | 0.605 | 0.344 | 1.065 | 0.081 | 1.305 | 0.816 | 2.088 | 0.268 |
| **H-WHI** | 1.081 | 0.776 | 1.504 | 0.646 | 0.938 | 0.583 | 1.511 | 0.794 | 1.278 | 0.773 | 2.114 | 0.34 |
| **H-WHR** | 0.978 | 0.702 | 1.362 | 0.894 | 0.841 | 0.522 | 1.356 | 0.479 | 1.125 | 0.71 | 1.784 | 0.617 |
| **H-%BF** | 0.778 | 0.557 | 1.087 | 0.142 | 0.8 | 0.495 | 1.292 | 0.362 | 0.75 | 0.469 | 1.199 | 0.23 |

| | | | | | | | | | | | | |
|---|---|---|---|---|---|---|---|---|---|---|---|---|
| **H-Insulin** | 0.767 | 0.462 | 1.274 | 0.306 | 0.764 | 0.387 | 1.507 | 0.439 | 0.786 | 0.361 | 1.708 | 0.544 |
| **H-Glucose** | 0.784 | 0.286 | 2.149 | 0.636 | 0.709 | 0.174 | 2.892 | 0.631 | 0.873 | 0.205 | 3.721 | 0.855 |
| **H-HOMA** | 0.757 | 0.457 | 1.253 | 0.279 | 0.73 | 0.374 | 1.424 | 0.356 | 0.779 | 0.359 | 1.693 | 0.529 |
| **H-Cholesterol** | 0.59 | 0.309 | 1.128 | 0.108 | 0.34 | 0.134 | 0.863 | 0.018 | 1.18 | 0.452 | 3.077 | 0.736 |
| **H-Triglycerides** | 0.811 | 0.538 | 1.222 | 0.316 | 0.818 | 0.472 | 1.415 | 0.473 | 0.792 | 0.423 | 1.483 | 0.467 |
| **L-HDL** | 0.917 | 0.652 | 1.289 | 0.619 | 0.818 | 0.472 | 1.415 | 0.473 | 0.993 | 0.63 | 1.565 | 0.977 |
| **H-LDL** | 0.331 | 0.123 | 0.89 | 0.022 | 0.177 | 0.04 | 0.789 | 0.011 | 0.724 | 0.178 | 2.948 | 0.652 |
| | | | | | | | **77958351** | | | | | |
| | | **Total** | | | | **Men** | | | | **Women** | | |
| **Clinical marker** | **OR** | **95% CI** | | ***p-value*** | **OR** | **95% CI** | | ***p-value*** | **OR** | **95% CI** | | ***p-value*** |
| **H-BMI** | 1.589 | 0.583 | 4.332 | 0.363 | 1.507 | 0.395 | 5.746 | 0.547 | 1.655 | 0.363 | 7.544 | 0.513 |
| **H- Waist** | 1.586 | 0.594 | 4.234 | 0.354 | 1.544 | 0.376 | 6.341 | 0.545 | 1.752 | 0.43 | 7.147 | 0.43 |
| **H-WHI** | 1.551 | 0.59 | 4.077 | 0.371 | 0.908 | 0.239 | 3.456 | 0.888 | 2.659 | 0.65 | 10.878 | 0.159 |
| **H-WHR** | 1.337 | 0.495 | 3.613 | 0.566 | 1.062 | 0.279 | 4.041 | 0.93 | 1.795 | 0.395 | 8.163 | 0.444 |
| **H-%BF** | 1.04 | 0.385 | 2.811 | 0.938 | 0.726 | 0.191 | 2.762 | 0.638 | 1.576 | 0.346 | 7.167 | 0.555 |
| **H-Insulin** | 0.742 | 0.163 | 3.386 | 0.7 | 0.403 | 0.049 | 3.308 | 0.385 | 1.575 | 0.17 | 14.573 | 0.688 |
| **H-Glucose** | 2.211 | 0.276 | 17.706 | 0.444 | 4.234 | 0.472 | 38.007 | 0.163 | 0.974 | 0.956 | 0.992 | 0.643 |
| **H-HOMA** | 0.436 | 0.057 | 3.366 | 0.414 | 0.817 | 0.77 | 0.867 | 0.159 | 1.713 | 0.194 | 15.158 | 0.626 |
| **H-Cholesterol** | 1.591 | 0.353 | 7.177 | 0.543 | 2.667 | 0.527 | 13.486 | 0.22 | 0.941 | 0.914 | 0.968 | 0.479 |
| **H-Triglycerides** | 2.191 | 0.794 | 6.048 | 0.121 | 2.482 | 0.648 | 9.508 | 0.172 | 1.771 | 0.347 | 9.036 | 0.488 |
| **L-HDL** | 1.249 | 0.469 | 3.331 | 0.656 | 0.853 | 0.173 | 4.203 | 0.846 | 1.966 | 0.462 | 8.375 | 0.354 |
| **H-LDL** | 1.337 | 0.171 | 10.468 | 0.782 | 2.055 | 0.242 | 17.45 | 0.502 | 0.967 | 0.947 | 0.987 | 0.603 |
| | | | | | | | **rs59634436** | | | | | |
| | | **Total** | | | | **Men** | | | | **Women** | | |
| **Clinical marker** | **OR** | **95% CI** | | ***p-value*** | **OR** | **95% CI** | | ***p-value*** | **OR** | **95% CI** | | ***p-value*** |
| **H-BMI** | 2.129 | 1.256 | 3.608 | 0.004 | 1.627 | 0.765 | 3.461 | 0.204 | 2.761 | 1.314 | 5.802 | 0.006 |
| **H- Waist** | 1.685 | 0.994 | 2.856 | 0.051 | 1.818 | 0.824 | 4.01 | 0.135 | 1.604 | 0.782 | 3.287 | 0.195 |
| **H-WHI** | 1.278 | 0.762 | 2.143 | 0.352 | 1.389 | 0.639 | 3.019 | 0.407 | 1.256 | 0.584 | 2.703 | 0.56 |
| **H-WHR** | 1.529 | 0.912 | 2.565 | 0.106 | 1.279 | 0.606 | 2.701 | 0.519 | 1.803 | 0.878 | 3.701 | 0.105 |

| | | | | | | | | | | | | |
|---|---|---|---|---|---|---|---|---|---|---|---|---|
| **H-%BF** | 1.879 | 1.091 | 3.238 | 0.021 | 2.333 | 1.027 | 5.299 | 0.039 | 1.569 | 0.749 | 3.287 | 0.231 |
| **H-Insulin** | 2.237 | 1.168 | 4.284 | 0.013 | 2.87 | 1.212 | 6.797 | 0.013 | 1.557 | 0.54 | 4.489 | 0.412 |
| **H-Glucose** | 3.597 | 1.225 | 10.561 | 0.013 | 7.407 | 1.876 | 29.251 | 0.001 | 1.16 | 0.138 | 9.726 | 0.891 |
| **H-HOMA** | 2.023 | 1.052 | 3.888 | 0.032 | 3.635 | 1.567 | 8.433 | 0.002 | 0.821 | 0.235 | 2.867 | 0.758 |
| **H-Cholesterol** | 1.519 | 0.65 | 3.553 | 0.332 | 1.426 | 0.46 | 4.419 | 0.538 | 1.677 | 0.46 | 6.12 | 0.43 |
| **H-Triglycerides** | 1.468 | 0.811 | 2.658 | 0.2 | 1.273 | 0.557 | 2.911 | 0.567 | 1.756 | 0.744 | 4.146 | 0.195 |
| **L-HDL** | 1.213 | 0.716 | 2.055 | 0.474 | 1.247 | 0.546 | 2.848 | 0.602 | 1.2 | 0.588 | 2.448 | 0.617 |
| **H-LDL** | 1.514 | 0.505 | 4.54 | 0.457 | 1.103 | 0.24 | 5.07 | 0.9 | 2.393 | 0.477 | 12.015 | 0.277 |
| **rs78160528** | | | | | | | | | | | | |
| | **Total** | | | | **Men** | | | | **Women** | | | |
| **Clinical marker** | **OR** | **95% CI** | | ***p-value*** | **OR** | **95% CI** | | ***p-value*** | **OR** | **95% CI** | | ***p-value*** |
| **H-BMI** | 1.008 | 0.373 | 2.728 | 0.988 | 1.119 | 0.262 | 4.782 | 0.88 | 0.932 | 0.236 | 3.684 | 0.92 |
| **H- Waist** | 1.438 | 0.548 | 3.771 | 0.459 | 1.014 | 0.2 | 5.143 | 0.986 | 1.759 | 0.498 | 6.214 | 0.376 |
| **H-WHI** | 1.092 | 0.425 | 2.807 | 0.856 | 0.722 | 0.177 | 2.947 | 0.65 | 1.756 | 0.483 | 6.382 | 0.388 |
| **H-WHR** | 1.339 | 0.524 | 3.423 | 0.542 | 1.336 | 0.327 | 5.452 | 0.687 | 1.341 | 0.38 | 4.733 | 0.648 |
| **H-%BF** | 1.175 | 0.447 | 3.089 | 0.744 | 1.229 | 0.27 | 5.595 | 0.791 | 1.176 | 0.333 | 4.15 | 0.802 |
| **H-Insulin** | 1.362 | 0.366 | 5.071 | 0.645 | 2.273 | 0.368 | 14.034 | 0.367 | 0.886 | 0.105 | 7.457 | 0.912 |
| **H-Glucose** | 0.971 | 0.957 | 0.985 | 0.462 | 0.968 | 0.947 | 0.989 | 0.608 | 0.973 | 0.955 | 0.992 | 0.603 |
| **H-HOMA** | 0.77 | 0.174 | 3.419 | 0.731 | 0.659 | 0.079 | 5.494 | 0.699 | 0.937 | 0.115 | 7.655 | 0.952 |
| **H-Cholesterol** | 1.489 | 0.332 | 6.68 | 0.602 | 3.123 | 0.601 | 16.244 | 0.156 | 0.94 | 0.914 | 0.967 | 0.427 |
| **H-Triglycerides** | 1.525 | 0.533 | 4.364 | 0.429 | 1.835 | 0.427 | 7.876 | 0.409 | 1.318 | 0.271 | 6.397 | 0.732 |
| **L-HDL** | 2.275 | 0.884 | 5.855 | 0.081 | 5.299 | 1.234 | 22.757 | 0.013 | 1.165 | 0.331 | 4.109 | 0.812 |
| **H-LDL** | 4.642 | 1.258 | 17.12 | 0.012 | 5.889 | 1.095 | 31.683 | 0.02 | 3.605 | 0.412 | 31.569 | 0.218 |
| **rs16997129** | | | | | | | | | | | | |
| | **Total** | | | | **Men** | | | | **Women** | | | |
| **Clinical marker** | **OR** | **95% CI** | | ***p-value*** | **OR** | **95% CI** | | ***p-value*** | **OR** | **95% CI** | | ***p-value*** |
| **H-BMI** | 0.894 | 0.616 | 1.296 | 0.554 | 0.882 | 0.523 | 1.488 | 0.639 | 0.913 | 0.537 | 1.553 | 0.739 |
| **H- Waist** | 0.901 | 0.619 | 1.311 | 0.585 | 1.053 | 0.59 | 1.878 | 0.863 | 0.772 | 0.466 | 1.278 | 0.316 |
| **H-WHI** | 0.896 | 0.63 | 1.275 | 0.542 | 1.259 | 0.762 | 2.078 | 0.37 | 0.646 | 0.38 | 1.1 | 0.107 |

| | | | | | | | | | | | | |
|---|---|---|---|---|---|---|---|---|---|---|---|---|
| **H-WHR** | 0.84 | 0.59 | 1.195 | 0.332 | 0.953 | 0.577 | 1.575 | 0.853 | 0.741 | 0.451 | 1.218 | 0.238 |
| **H-%BF** | 1.022 | 0.714 | 1.461 | 0.907 | 0.691 | 0.413 | 1.155 | 0.159 | 1.531 | 0.916 | 2.559 | 0.104 |
| **H-Insulin** | 1.064 | 0.617 | 1.836 | 0.824 | 0.902 | 0.439 | 1.853 | 0.78 | 1.297 | 0.549 | 3.066 | 0.555 |
| **H-Glucose** | 1.439 | 0.463 | 4.475 | 0.528 | 1.676 | 0.341 | 8.228 | 0.522 | 1.234 | 0.244 | 6.23 | 0.8 |
| **H-HOMA** | 0.929 | 0.551 | 1.566 | 0.781 | 0.883 | 0.445 | 1.753 | 0.723 | 1.023 | 0.451 | 2.322 | 0.957 |
| **H-Cholesterol** | 0.93 | 0.491 | 1.764 | 0.825 | 1.917 | 0.752 | 4.882 | 0.168 | 0.384 | 0.147 | 1.001 | 0.044 |
| **H-Triglycerides** | 0.76 | 0.499 | 1.158 | 0.201 | 0.851 | 0.484 | 1.497 | 0.577 | 0.681 | 0.36 | 1.289 | 0.237 |
| **L-HDL** | 1.055 | 0.733 | 1.518 | 0.775 | 1.007 | 0.568 | 1.784 | 0.981 | 1.033 | 0.631 | 1.69 | 0.897 |
| **H-LDL** | 1.887 | 0.7 | 5.084 | 0.203 | 2.295 | 0.643 | 8.191 | 0.191 | 1.446 | 0.295 | 7.098 | 0.649 |
| **rs3813827** | | | | | | | | | | | | |
| | **Total** | | | | **Men** | | | | **Women** | | | |
| **Clinical marker** | **OR** | **95% CI** | | ***p-value*** | **OR** | **95% CI** | | ***p-value*** | **OR** | **95% CI** | | ***p-value*** |
| **H-BMI** | 2.669 | 0.979 | 7.277 | 0.047 | 2.394 | 0.628 | 9.125 | 0.19 | 2.989 | 0.656 | 13.628 | 0.14 |
| **H- Waist** | 0.739 | 0.235 | 2.322 | 0.604 | 0.371 | 0.046 | 3.022 | 0.337 | 1.302 | 0.286 | 5.926 | 0.733 |
| **H-WHI** | 1.373 | 0.508 | 3.709 | 0.531 | 0.908 | 0.239 | 3.456 | 0.888 | 1.97 | 0.432 | 8.99 | 0.375 |
| **H-WHR** | 0.791 | 0.284 | 2.206 | 0.654 | 0.654 | 0.16 | 2.669 | 0.553 | 0.996 | 0.219 | 4.529 | 0.996 |
| **H-%BF** | 1.577 | 0.554 | 4.491 | 0.39 | 1.151 | 0.302 | 4.381 | 0.837 | 2.379 | 0.429 | 13.193 | 0.309 |
| **H-Insulin** | 0.812 | 0.176 | 3.74 | 0.79 | 0.463 | 0.056 | 3.868 | 0.47 | 1.575 | 0.17 | 14.573 | 0.688 |
| **H-Glucose** | 0.971 | 0.957 | 0.985 | 0.489 | 0.968 | 0.947 | 0.989 | 0.586 | 0.974 | 0.956 | 0.992 | 0.665 |
| **H-HOMA** | 0.954 | 0.211 | 4.315 | 0.952 | 0.574 | 0.07 | 4.706 | 0.602 | 1.713 | 0.194 | 15.158 | 0.626 |
| **H-Cholesterol** | 0.778 | 0.101 | 6.024 | 0.81 | 0.896 | 0.861 | 0.933 | 0.309 | 2.814 | 0.32 | 24.712 | 0.332 |
| **H-Triglycerides** | 0.9 | 0.252 | 3.209 | 0.871 | 0.853 | 0.173 | 4.203 | 0.846 | 0.867 | 0.102 | 7.364 | 0.897 |
| **L-HDL** | 0.801 | 0.275 | 2.338 | 0.685 | 1.522 | 0.371 | 6.248 | 0.559 | 0.456 | 0.087 | 2.389 | 0.343 |
| **H-LDL** | 1.428 | 0.182 | 11.229 | 0.734 | 0.939 | 0.911 | 0.968 | 0.447 | 5.463 | 0.594 | 50.222 | 0.094 |
| **rs11737840** | | | | | | | | | | | | |
| | **Total** | | | | **Men** | | | | **Women** | | | |
| **Clinical marker** | **OR** | **95% CI** | | ***p-value*** | **OR** | **95% CI** | | ***p-value*** | **OR** | **95% CI** | | ***p-value*** |
| **H-BMI** | 1.212 | 0.807 | 1.82 | 0.354 | 1.324 | 0.759 | 2.31 | 0.324 | 1.073 | 0.589 | 1.956 | 0.816 |
| **H- Waist** | 1.308 | 0.869 | 1.967 | 0.198 | 1.175 | 0.637 | 2.167 | 0.607 | 1.55 | 0.881 | 2.728 | 0.128 |

| | | | | | | | | | | | | |
|---|---|---|---|---|---|---|---|---|---|---|---|---|
| **H-WHI** | 1.379 | 0.935 | 2.034 | 0.105 | 1.329 | 0.763 | 2.313 | 0.316 | 1.291 | 0.707 | 2.36 | 0.406 |
| **H-WHR** | 1.044 | 0.706 | 1.542 | 0.83 | 1.063 | 0.617 | 1.83 | 0.827 | 1.024 | 0.583 | 1.797 | 0.935 |
| **H-%BF** | 1.139 | 0.769 | 1.689 | 0.516 | 1.009 | 0.585 | 1.742 | 0.973 | 1.259 | 0.711 | 2.229 | 0.431 |
| **H-Insulin** | 1.085 | 0.61 | 1.929 | 0.781 | 1.631 | 0.792 | 3.361 | 0.184 | 0.484 | 0.161 | 1.457 | 0.19 |
| **H-Glucose** | 1.471 | 0.509 | 4.254 | 0.474 | 1.575 | 0.383 | 6.468 | 0.527 | 1.317 | 0.259 | 6.686 | 0.74 |
| **H-HOMA** | 0.916 | 0.506 | 1.656 | 0.771 | 1.127 | 0.542 | 2.343 | 0.751 | 0.555 | 0.186 | 1.653 | 0.286 |
| **H-Cholesterol** | 1.072 | 0.53 | 2.168 | 0.847 | 1.204 | 0.508 | 2.854 | 0.674 | 0.773 | 0.217 | 2.758 | 0.692 |
| **H-Triglycerides** | 0.876 | 0.537 | 1.428 | 0.596 | 1.045 | 0.563 | 1.939 | 0.89 | 0.593 | 0.253 | 1.39 | 0.226 |
| **L-HDL** | 0.914 | 0.61 | 1.37 | 0.665 | 1.153 | 0.626 | 2.124 | 0.65 | 0.833 | 0.477 | 1.456 | 0.523 |
| **H-LDL** | 1.499 | 0.641 | 3.505 | 0.348 | 2.3 | 0.841 | 6.291 | 0.097 | 0.427 | 0.053 | 3.431 | 0.41 |

| **rs10033691** | | | | | | | | | | | | |
|---|---|---|---|---|---|---|---|---|---|---|---|---|
| | **Total** | | | | **Men** | | | | **Women** | | | |
| **Clinical marker** | **OR** | **95% CI** | | ***p-value*** | **OR** | **95% CI** | | ***p-value*** | **OR** | **95% CI** | | ***p-value*** |
| **H-BMI** | 1.844 | 1.14 | 2.983 | 0.012 | 1.609 | 0.805 | 3.216 | 0.177 | 2.102 | 1.076 | 4.106 | 0.027 |
| **H- Waist** | 1.463 | 0.9 | 2.379 | 0.124 | 1.965 | 0.954 | 4.046 | 0.064 | 1.151 | 0.595 | 2.228 | 0.677 |
| **H-WHI** | 1.312 | 0.82 | 2.099 | 0.257 | 1.473 | 0.72 | 3.013 | 0.288 | 1.294 | 0.647 | 2.587 | 0.467 |
| **H-WHR** | 1.349 | 0.844 | 2.159 | 0.211 | 1.385 | 0.699 | 2.744 | 0.352 | 1.319 | 0.691 | 2.517 | 0.402 |
| **H-%BF** | 2.27 | 1.371 | 3.759 | 0.001 | 3.299 | 1.493 | 7.288 | 0.002 | 1.7 | 0.867 | 3.335 | 0.121 |
| **H-Insulin** | 2.094 | 1.132 | 3.874 | 0.017 | 2.824 | 1.232 | 6.469 | 0.011 | 1.476 | 0.554 | 3.929 | 0.436 |
| **H-Glucose** | 3.687 | 1.325 | 10.265 | 0.008 | 5.765 | 1.475 | 22.524 | 0.005 | 2.13 | 0.416 | 10.913 | 0.355 |
| **H-HOMA** | 1.831 | 0.991 | 3.385 | 0.051 | 2.957 | 1.342 | 6.514 | 0.005 | 0.901 | 0.298 | 2.723 | 0.854 |
| **H-Cholesterol** | 1.13 | 0.488 | 2.617 | 0.775 | 0.738 | 0.212 | 2.57 | 0.634 | 1.861 | 0.583 | 5.943 | 0.289 |
| **H-Triglycerides** | 1.337 | 0.772 | 2.317 | 0.3 | 1.083 | 0.498 | 2.355 | 0.841 | 1.727 | 0.789 | 3.78 | 0.168 |
| **L-HDL** | 1.116 | 0.689 | 1.809 | 0.656 | 0.921 | 0.414 | 2.051 | 0.841 | 1.254 | 0.658 | 2.388 | 0.493 |
| **H-LDL** | 1.483 | 0.545 | 4.034 | 0.438 | 0.395 | 0.051 | 3.07 | 0.36 | 4.479 | 1.21 | 16.584 | 0.015 |

| **rs2005701** | | | | | | | | | | | | |
|---|---|---|---|---|---|---|---|---|---|---|---|---|
| | **Total** | | | | **Men** | | | | **Women** | | | |
| **Clinical marker** | **OR** | **95% CI** | | ***p-value*** | **OR** | **95% CI** | | ***p-value*** | **OR** | **95% CI** | | ***p-value*** |
| **H-BMI** | 1.285 | 0.886 | 1.863 | 0.186 | 0.959 | 0.57 | 1.615 | 0.876 | 1.717 | 1.004 | 2.937 | 0.047 |

| | | | | | | | | | | | | |
|---|---|---|---|---|---|---|---|---|---|---|---|---|
| **H- Waist** | 1.396 | 0.957 | 2.037 | 0.083 | 0.824 | 0.47 | 1.444 | 0.5 | 2.163 | 1.287 | 3.634 | 0.003 |
| **H-WHI** | 1.339 | 0.945 | 1.896 | 0.1 | 1.138 | 0.692 | 1.871 | 0.612 | 1.659 | 0.958 | 2.876 | 0.07 |
| **H-WHR** | 1.219 | 0.861 | 1.726 | 0.265 | 0.92 | 0.559 | 1.514 | 0.744 | 1.589 | 0.975 | 2.59 | 0.063 |
| **H-%BF** | 1.152 | 0.813 | 1.631 | 0.428 | 1.202 | 0.722 | 2 | 0.481 | 1.086 | 0.672 | 1.754 | 0.737 |
| **H-Insulin** | 1.133 | 0.662 | 1.94 | 0.649 | 1.607 | 0.769 | 3.356 | 0.207 | 0.768 | 0.346 | 1.705 | 0.518 |
| **H-Glucose** | 1.583 | 0.504 | 4.972 | 0.428 | 1.011 | 0.247 | 4.133 | 0.988 | 3.292 | 0.391 | 27.707 | 0.247 |
| **H-HOMA** | 1.07 | 0.639 | 1.791 | 0.798 | 1.456 | 0.709 | 2.99 | 0.306 | 0.721 | 0.338 | 1.538 | 0.397 |
| **H-Cholesterol** | 0.502 | 0.231 | 1.089 | 0.214 | 0.682 | 0.373 | 1.249 | 0.077 | 1.079 | 0.393 | 2.961 | 0.883 |
| **H-Triglycerides** | 1.088 | 0.713 | 1.661 | 0.696 | 1.014 | 0.576 | 1.786 | 0.962 | 1.171 | 0.613 | 2.234 | 0.634 |
| **L-HDL** | 0.929 | 0.654 | 1.32 | 0.682 | 0.676 | 0.389 | 1.174 | 0.164 | 1.187 | 0.741 | 1.899 | 0.477 |
| **H-LDL** | 0.828 | 0.369 | 1.859 | 0.647 | 0.706 | 0.26 | 1.918 | 0.495 | 1.077 | 0.264 | 4.394 | 0.918 |
| | | | | | | | **rs76145164** | | | | | |
| | | **Total** | | | | **Men** | | | | **Women** | | |
| **Clinical marker** | **OR** | **95% CI** | | ***p-value*** | **OR** | **95% CI** | | ***p-value*** | **OR** | **95% CI** | | ***p-value*** |
| **H-BMI** | 1.528 | 0.633 | 3.692 | 0.343 | 0.684 | 0.177 | 2.637 | 0.58 | 3.404 | 0.938 | 12.362 | 0.049 |
| **H- Waist** | 0.885 | 0.338 | 2.32 | 0.805 | 0.663 | 0.14 | 3.145 | 0.604 | 1.15 | 0.317 | 4.164 | 0.832 |
| **H-WHI** | 1.511 | 0.631 | 3.614 | 0.352 | 1.277 | 0.365 | 4.463 | 0.703 | 1.748 | 0.481 | 6.353 | 0.392 |
| **H-WHR** | 0.65 | 0.258 | 1.634 | 0.357 | 0.481 | 0.125 | 1.853 | 0.28 | 0.877 | 0.242 | 3.172 | 0.842 |
| **H-%BF** | 0.772 | 0.32 | 1.862 | 0.565 | 0.514 | 0.147 | 1.797 | 0.291 | 1.168 | 0.331 | 4.123 | 0.81 |
| **H-Insulin** | 1.399 | 0.444 | 4.415 | 0.566 | 1.698 | 0.407 | 7.073 | 0.465 | 0.886 | 0.105 | 7.457 | 0.912 |
| **H-Glucose** | 0.97 | 0.957 | 0.984 | 0.425 | 0.967 | 0.947 | 0.989 | 0.544 | 0.973 | 0.955 | 0.992 | 0.602 |
| **H-HOMA** | 1.48 | 0.484 | 4.525 | 0.49 | 1.795 | 0.457 | 7.048 | 0.398 | 0.933 | 0.114 | 7.625 | 0.949 |
| **H-Cholesterol** | 0.576 | 0.076 | 4.39 | 0.591 | 0.895 | 0.859 | 0.932 | 0.258 | 1.85 | 0.221 | 15.459 | 0.566 |
| **H-Triglycerides** | 3.073 | 1.264 | 7.471 | 0.009 | 1.748 | 0.496 | 6.154 | 0.381 | 5.667 | 1.576 | 20.37 | 0.003 |
| **L-HDL** | 1.347 | 0.558 | 3.251 | 0.507 | 0.654 | 0.138 | 3.1 | 0.592 | 2.814 | 0.714 | 11.089 | 0.125 |
| **H-LDL** | 0.953 | 0.936 | 0.971 | 0.311 | 0.938 | 0.91 | 0.967 | 0.398 | 0.967 | 0.947 | 0.987 | 0.559 |
| | | | | | | | **rs76338299** | | | | | |
| | | **Total** | | | | **Men** | | | | **Women** | | |
| **Clinical marker** | **OR** | **95% CI** | | ***p-value*** | **OR** | **95% CI** | | ***p-value*** | **OR** | **95% CI** | | ***p-value*** |

| | | | | | | | | | | | | |
|---|---|---|---|---|---|---|---|---|---|---|---|---|
| **H-BMI** | 0.916 | 0.502 | 1.673 | 0.776 | 1.097 | 0.482 | 2.497 | 0.826 | 0.744 | 0.303 | 1.824 | 0.518 |
| **H- Waist** | 1.234 | 0.685 | 2.221 | 0.484 | 0.853 | 0.33 | 2.204 | 0.743 | 1.681 | 0.76 | 3.716 | 0.197 |
| **H-WHI** | 1.095 | 0.623 | 1.923 | 0.753 | 0.758 | 0.343 | 1.678 | 0.495 | 1.587 | 0.696 | 3.618 | 0.271 |
| **H-WHR** | 1.064 | 0.606 | 1.87 | 0.829 | 0.755 | 0.333 | 1.712 | 0.501 | 1.482 | 0.672 | 3.27 | 0.329 |
| **H-%BF** | 1.187 | 0.674 | 2.091 | 0.553 | 1.172 | 0.527 | 2.605 | 0.698 | 1.187 | 0.531 | 2.656 | 0.677 |
| **H-Insulin** | 0.802 | 0.324 | 1.986 | 0.633 | 0.762 | 0.243 | 2.389 | 0.642 | 0.763 | 0.167 | 3.494 | 0.728 |
| **H-Glucose** | 0.969 | 0.954 | 0.983 | 0.184 | 0.965 | 0.943 | 0.988 | 0.328 | 0.972 | 0.953 | 0.991 | 0.369 |
| **H-HOMA** | 0.757 | 0.312 | 1.836 | 0.538 | 0.793 | 0.26 | 2.417 | 0.685 | 0.654 | 0.147 | 2.904 | 0.575 |
| **H-Cholesterol** | 0.654 | 0.196 | 2.181 | 0.488 | 0.319 | 0.042 | 2.44 | 0.248 | 1.284 | 0.28 | 5.893 | 0.748 |
| **H-Triglycerides** | 0.543 | 0.239 | 1.231 | 0.139 | 0.655 | 0.238 | 1.798 | 0.41 | 0.377 | 0.086 | 1.64 | 0.178 |
| **L-HDL** | 0.848 | 0.47 | 1.529 | 0.583 | 1.563 | 0.669 | 3.654 | 0.301 | 0.519 | 0.227 | 1.187 | 0.116 |
| **H-LDL** | 1.773 | 0.59 | 5.327 | 0.302 | 1.307 | 0.282 | 6.045 | 0.733 | 2.644 | 0.533 | 13.108 | 0.218 |
| | | | | | | | **rs6533491** | | | | | |
| | | **Total** | | | | **Men** | | | | **Women** | | |
| **Clinical marker** | **OR** | **95% CI** | | ***p-value*** | **OR** | **95% CI** | | ***p-value*** | **OR** | **95% CI** | | ***p-value*** |
| **H-BMI** | 0.893 | 0.633 | 1.261 | 0.521 | 0.796 | 0.488 | 1.298 | 0.361 | 1.012 | 0.621 | 1.647 | 0.963 |
| **H- Waist** | 0.676 | 0.476 | 0.958 | 0.028 | 0.886 | 0.518 | 1.514 | 0.658 | 0.526 | 0.329 | 0.841 | 0.007 |
| **H-WHI** | 0.704 | 0.508 | 0.977 | 0.035 | 0.877 | 0.549 | 1.402 | 0.586 | 0.559 | 0.338 | 0.925 | 0.023 |
| **H-WHR** | 0.817 | 0.589 | 1.132 | 0.225 | 0.918 | 0.575 | 1.466 | 0.721 | 0.731 | 0.463 | 1.153 | 0.179 |
| **H-%BF** | 0.948 | 0.682 | 1.317 | 0.751 | 0.663 | 0.412 | 1.066 | 0.09 | 1.345 | 0.848 | 2.133 | 0.209 |
| **H-Insulin** | 1.325 | 0.806 | 2.177 | 0.268 | 1.086 | 0.561 | 2.104 | 0.807 | 1.618 | 0.751 | 3.486 | 0.218 |
| **H-Glucose** | 1.047 | 0.398 | 2.75 | 0.927 | 0.794 | 0.209 | 3.02 | 0.736 | 1.46 | 0.343 | 6.217 | 0.608 |
| **H-HOMA** | 0.898 | 0.553 | 1.458 | 0.663 | 0.77 | 0.403 | 1.471 | 0.43 | 1.133 | 0.536 | 2.394 | 0.744 |
| **H-Cholesterol** | 1.061 | 0.584 | 1.926 | 0.847 | 1.26 | 0.582 | 2.724 | 0.559 | 0.86 | 0.332 | 2.228 | 0.757 |
| **H-Triglycerides** | 0.99 | 0.665 | 1.474 | 0.961 | 1 | 0.587 | 1.705 | 1 | 1.022 | 0.557 | 1.875 | 0.944 |
| **L-HDL** | 1.283 | 0.917 | 1.795 | 0.145 | 1.346 | 0.788 | 2.301 | 0.278 | 1.196 | 0.764 | 1.873 | 0.435 |
| **H-LDL** | 1.28 | 0.578 | 2.836 | 0.542 | 1.902 | 0.684 | 5.29 | 0.213 | 0.686 | 0.181 | 2.606 | 0.58 |
| | | | | | | | **rs11937052** | | | | | |
| | | **Total** | | | | **Men** | | | | **Women** | | |

| Clinical marker | OR | 95% CI | | *p-value* | OR | 95% CI | | *p-value* | OR | 95% CI | | *p-value* |
|---|---|---|---|---|---|---|---|---|---|---|---|---|
| **H-BMI** | 1.797 | 1.113 | 2.901 | 0.015 | 1.609 | 0.805 | 3.216 | 0.177 | 2 | 1.03 | 3.884 | 0.038 |
| **H- Waist** | 1.776 | 1.104 | 2.858 | 0.017 | 2.441 | 1.206 | 4.94 | 0.011 | 1.374 | 0.719 | 2.626 | 0.336 |
| **H-WHI** | 1.05 | 0.657 | 1.676 | 0.84 | 1.543 | 0.757 | 3.143 | 0.231 | 0.728 | 0.342 | 1.546 | 0.408 |
| **H-WHR** | 1.208 | 0.758 | 1.924 | 0.427 | 1.658 | 0.841 | 3.269 | 0.142 | 0.907 | 0.474 | 1.735 | 0.768 |
| **H-%BF** | 1.497 | 0.924 | 2.426 | 0.1 | 2.101 | 1.009 | 4.375 | 0.044 | 1.133 | 0.585 | 2.193 | 0.712 |
| **H-Insulin** | 1.359 | 0.693 | 2.665 | 0.372 | 1.529 | 0.646 | 3.62 | 0.334 | 1.043 | 0.336 | 3.242 | 0.942 |
| **H-Glucose** | 1.959 | 0.623 | 6.159 | 0.242 | 5.577 | 1.429 | 21.766 | 0.006 | 0.97 | 0.95 | 0.991 | 0.246 |
| **H-HOMA** | 1.34 | 0.697 | 2.575 | 0.38 | 1.769 | 0.765 | 4.09 | 0.179 | 0.901 | 0.298 | 2.723 | 0.854 |
| **H-Cholesterol** | 1.53 | 0.711 | 3.293 | 0.275 | 1.379 | 0.493 | 3.857 | 0.541 | 1.807 | 0.566 | 5.766 | 0.313 |
| **H-Triglycerides** | 1.19 | 0.683 | 2.075 | 0.539 | 1.4 | 0.668 | 2.933 | 0.373 | 0.986 | 0.412 | 2.355 | 0.974 |
| **L-HDL** | 1.35 | 0.842 | 2.163 | 0.212 | 2.105 | 1.035 | 4.28 | 0.037 | 0.961 | 0.507 | 1.823 | 0.904 |
| **H-LDL** | 0.769 | 0.226 | 2.612 | 0.673 | 0.843 | 0.185 | 3.839 | 0.826 | 0.667 | 0.082 | 5.396 | 0.703 |
| | | | | | | **rs11098070** | | | | | | |
| | | **Total** | | | | **Men** | | | | **Women** | | |
| **Clinical marker** | **OR** | **95% CI** | | ***p-value*** | **OR** | **95% CI** | | ***p-value*** | **OR** | **95% CI** | | ***p-value*** |
| **H-BMI** | 1.209 | 0.811 | 1.803 | 0.351 | 1.151 | 0.655 | 2.024 | 0.626 | 1.269 | 0.721 | 2.236 | 0.41 |
| **H- Waist** | 1.126 | 0.754 | 1.682 | 0.563 | 1.278 | 0.679 | 2.407 | 0.449 | 1.033 | 0.608 | 1.754 | 0.905 |
| **H-WHI** | 0.966 | 0.666 | 1.4 | 0.855 | 1.211 | 0.711 | 2.065 | 0.482 | 0.746 | 0.429 | 1.297 | 0.3 |
| **H-WHR** | 1.125 | 0.773 | 1.637 | 0.539 | 1.219 | 0.709 | 2.095 | 0.475 | 1.044 | 0.62 | 1.757 | 0.872 |
| **H-%BF** | 1.104 | 0.758 | 1.606 | 0.607 | 1.413 | 0.823 | 2.425 | 0.21 | 0.877 | 0.52 | 1.479 | 0.624 |
| **H-Insulin** | 1.094 | 0.618 | 1.935 | 0.759 | 1.755 | 0.756 | 4.074 | 0.189 | 0.636 | 0.285 | 1.419 | 0.268 |
| **H-Glucose** | 1.661 | 0.471 | 5.86 | 0.426 | 1.044 | 1.015 | 1.074 | 0.072 | 0.58 | 0.136 | 2.485 | 0.46 |
| **H-HOMA** | 1.234 | 0.693 | 2.198 | 0.476 | 2.099 | 0.887 | 4.966 | 0.087 | 0.696 | 0.312 | 1.556 | 0.377 |
| **H-Cholesterol** | 2.051 | 0.898 | 4.687 | 0.083 | 1.762 | 0.647 | 4.799 | 0.264 | 2.769 | 0.619 | 12.381 | 0.167 |
| **H-Triglycerides** | 0.986 | 0.627 | 1.55 | 0.95 | 0.965 | 0.526 | 1.771 | 0.909 | 1.008 | 0.506 | 2.009 | 0.982 |
| **L-HDL** | 0.748 | 0.514 | 1.088 | 0.129 | 0.965 | 0.526 | 1.771 | 0.909 | 0.609 | 0.366 | 1.015 | 0.056 |
| **H-LDL** | 1.575 | 0.586 | 4.234 | 0.365 | 1.143 | 0.361 | 3.622 | 0.821 | 3.273 | 0.408 | 26.243 | 0.239 |
| | | | | | | **rs80343897** | | | | | | |

| | Total | | | | Men | | | | Women | | | |
|---|---|---|---|---|---|---|---|---|---|---|---|---|
| Clinical marker | OR | 95% CI | | *p-value* | OR | 95% CI | | *p-value* | OR | 95% CI | | *p-value* |
| H-BMI | 0.886 | 0.623 | 1.262 | 0.505 | 0.885 | 0.537 | 1.46 | 0.634 | 0.889 | 0.539 | 1.466 | 0.645 |
| H- Waist | 0.916 | 0.641 | 1.309 | 0.63 | 0.747 | 0.428 | 1.303 | 0.304 | 1.062 | 0.661 | 1.705 | 0.806 |
| H-WHI | 1.188 | 0.853 | 1.654 | 0.31 | 1.12 | 0.694 | 1.807 | 0.644 | 1.318 | 0.796 | 2.181 | 0.284 |
| H-WHR | 1.082 | 0.776 | 1.508 | 0.642 | 1.007 | 0.625 | 1.621 | 0.978 | 1.159 | 0.729 | 1.841 | 0.535 |
| H-%BF | 1.022 | 0.731 | 1.428 | 0.901 | 1.171 | 0.723 | 1.899 | 0.523 | 0.905 | 0.568 | 1.444 | 0.678 |
| H-Insulin | 0.708 | 0.422 | 1.189 | 0.192 | 0.562 | 0.278 | 1.136 | 0.107 | 0.935 | 0.429 | 2.037 | 0.866 |
| H-Glucose | 0.202 | 0.045 | 0.895 | 0.02 | 0.407 | 0.083 | 1.997 | 0.255 | 0.962 | 0.934 | 0.99 | 0.026 |
| H-HOMA | 0.801 | 0.486 | 1.322 | 0.386 | 0.667 | 0.339 | 1.312 | 0.24 | 1.016 | 0.478 | 2.162 | 0.966 |
| H-Cholesterol | 1.577 | 0.868 | 2.865 | 0.132 | 1.649 | 0.764 | 3.561 | 0.201 | 1.487 | 0.573 | 3.858 | 0.414 |
| H-Triglycerides | 0.931 | 0.62 | 1.399 | 0.732 | 0.981 | 0.57 | 1.689 | 0.945 | 0.871 | 0.467 | 1.622 | 0.664 |
| L-HDL | 0.654 | 0.462 | 0.924 | 0.016 | 0.609 | 0.347 | 1.07 | 0.084 | 0.658 | 0.416 | 1.041 | 0.074 |
| H-LDL | 1.373 | 0.634 | 2.974 | 0.421 | 1.321 | 0.494 | 3.53 | 0.579 | 1.471 | 0.417 | 5.191 | 0.548 |

| | | | | | rs114422025 | | | | | | | |
|---|---|---|---|---|---|---|---|---|---|---|---|---|
| | Total | | | | Men | | | | Women | | | |
| Clinical marker | OR | 95% CI | | *p-value* | OR | 95% CI | | *p-value* | OR | 95% CI | | *p-value* |
| H-BMI | 1.267 | 0.409 | 3.924 | 0.682 | 0.928 | 0.167 | 5.157 | 0.932 | 1.655 | 0.363 | 7.544 | 0.513 |
| H- Waist | 1.407 | 0.454 | 4.361 | 0.553 | 1.536 | 0.275 | 8.57 | 0.623 | 1.302 | 0.286 | 5.926 | 0.733 |
| H-WHI | 0.848 | 0.274 | 2.622 | 0.774 | 1.469 | 0.265 | 8.154 | 0.659 | 0.424 | 0.05 | 3.57 | 0.418 |
| H-WHR | 0.826 | 0.267 | 2.555 | 0.74 | 1.333 | 0.264 | 6.722 | 0.728 | 0.524 | 0.1 | 2.744 | 0.439 |
| H-%BF | 1.04 | 0.331 | 3.264 | 0.946 | 1.383 | 0.227 | 8.408 | 0.725 | 0.874 | 0.192 | 3.976 | 0.862 |
| H-Insulin | 3.105 | 0.855 | 11.282 | 0.071 | 1.693 | 0.3 | 9.554 | 0.549 | 6.586 | 0.893 | 48.593 | 0.035 |
| H-Glucose | 0.971 | 0.957 | 0.985 | 0.534 | 0.968 | 0.948 | 0.989 | 0.658 | 0.974 | 0.956 | 0.992 | 0.665 |
| H-HOMA | 4.678 | 1.445 | 15.141 | 0.005 | 4.929 | 0.961 | 25.264 | 0.036 | 4.448 | 0.781 | 25.35 | 0.068 |
| H-Cholesterol | 0.978 | 0.124 | 7.691 | 0.983 | 1.814 | 0.205 | 16.085 | 0.589 | 0.941 | 0.915 | 0.968 | 0.509 |
| H-Triglycerides | 2.505 | 0.805 | 7.799 | 0.102 | 3.087 | 0.609 | 15.648 | 0.154 | 2.133 | 0.402 | 11.316 | 0.364 |
| L-HDL | 2.104 | 0.698 | 6.344 | 0.178 | 1.514 | 0.271 | 8.446 | 0.635 | 2.968 | 0.567 | 15.534 | 0.179 |
| H-LDL | 1.795 | 0.225 | 14.332 | 0.577 | 3.325 | 0.366 | 30.174 | 0.26 | 0.967 | 0.947 | 0.987 | 0.627 |

| rs6533495 | | | | | | | | | | | | |
|---|---|---|---|---|---|---|---|---|---|---|---|---|
| | Total | | | | Men | | | | Women | | | |
| **Clinical marker** | **OR** | **95% CI** | | ***p-value*** | **OR** | **95% CI** | | ***p-value*** | **OR** | **95% CI** | | ***p-value*** |
| **H-BMI** | 0.926 | 0.637 | 1.347 | 0.687 | 0.887 | 0.522 | 1.51 | 0.661 | 0.964 | 0.568 | 1.637 | 0.893 |
| **H- Waist** | 0.881 | 0.604 | 1.285 | 0.512 | 0.771 | 0.434 | 1.368 | 0.375 | 0.978 | 0.589 | 1.624 | 0.933 |
| **H-WHI** | 0.767 | 0.538 | 1.093 | 0.143 | 0.714 | 0.424 | 1.204 | 0.207 | 0.77 | 0.451 | 1.315 | 0.339 |
| **H-WHR** | 0.852 | 0.597 | 1.217 | 0.38 | 0.717 | 0.43 | 1.196 | 0.203 | 1.003 | 0.61 | 1.647 | 0.991 |
| **H-%BF** | 1.03 | 0.719 | 1.475 | 0.874 | 0.919 | 0.548 | 1.541 | 0.749 | 1.148 | 0.693 | 1.9 | 0.594 |
| **H-Insulin** | 0.81 | 0.476 | 1.379 | 0.439 | 0.894 | 0.435 | 1.836 | 0.761 | 0.706 | 0.317 | 1.572 | 0.395 |
| **H-Glucose** | 0.773 | 0.281 | 2.123 | 0.617 | 0.511 | 0.134 | 1.953 | 0.32 | 1.3 | 0.258 | 6.563 | 0.751 |
| **H-HOMA** | 0.989 | 0.58 | 1.688 | 0.968 | 1.179 | 0.572 | 2.43 | 0.658 | 0.804 | 0.361 | 1.791 | 0.595 |
| **H-Cholesterol** | 1.819 | 0.859 | 3.853 | 0.114 | 1.354 | 0.556 | 3.301 | 0.505 | 3.396 | 0.761 | 15.158 | 0.091 |
| **H-Triglycerides** | 1.019 | 0.659 | 1.575 | 0.934 | 0.787 | 0.444 | 1.395 | 0.414 | 1.441 | 0.715 | 2.902 | 0.306 |
| **L-HDL** | 0.798 | 0.556 | 1.145 | 0.22 | 0.665 | 0.377 | 1.171 | 0.158 | 0.903 | 0.555 | 1.469 | 0.681 |
| **H-LDL** | 1.917 | 0.714 | 5.146 | 0.19 | 2.025 | 0.567 | 7.236 | 0.27 | 1.75 | 0.364 | 8.403 | 0.481 |

| rs28722886 | | | | | | | | | | | | |
|---|---|---|---|---|---|---|---|---|---|---|---|---|
| | Total | | | | Men | | | | Women | | | |
| **Clinical marker** | **OR** | **95% CI** | | ***p-value*** | **OR** | **95% CI** | | ***p-value*** | **OR** | **95% CI** | | ***p-value*** |
| **H-BMI** | 0.845 | 0.594 | 1.201 | 0.347 | 1.041 | 0.633 | 1.713 | 0.874 | 0.69 | 0.419 | 1.138 | 0.146 |
| **H- Waist** | 0.856 | 0.601 | 1.22 | 0.392 | 0.866 | 0.5 | 1.498 | 0.608 | 0.821 | 0.512 | 1.314 | 0.412 |
| **H-WHI** | 1.304 | 0.937 | 1.816 | 0.116 | 1.42 | 0.878 | 2.298 | 0.153 | 1.365 | 0.825 | 2.258 | 0.226 |
| **H-WHR** | 1.144 | 0.821 | 1.592 | 0.428 | 1.375 | 0.853 | 2.217 | 0.192 | 0.962 | 0.608 | 1.524 | 0.871 |
| **H-%BF** | 0.928 | 0.665 | 1.295 | 0.66 | 1.054 | 0.651 | 1.704 | 0.832 | 0.832 | 0.522 | 1.325 | 0.439 |
| **H-Insulin** | 1.1 | 0.666 | 1.818 | 0.711 | 0.896 | 0.452 | 1.777 | 0.754 | 1.597 | 0.735 | 3.47 | 0.237 |
| **H-Glucose** | 0.703 | 0.256 | 1.926 | 0.491 | 0.386 | 0.079 | 1.891 | 0.225 | 1.233 | 0.303 | 5.024 | 0.771 |
| **H-HOMA** | 1.033 | 0.629 | 1.695 | 0.899 | 0.788 | 0.402 | 1.546 | 0.49 | 1.527 | 0.715 | 3.261 | 0.273 |
| **H-Cholesterol** | 1.158 | 0.637 | 2.106 | 0.63 | 1.556 | 0.72 | 3.36 | 0.259 | 0.769 | 0.29 | 2.039 | 0.597 |
| **H-Triglycerides** | 1.177 | 0.787 | 1.76 | 0.428 | 1.101 | 0.64 | 1.894 | 0.73 | 1.339 | 0.726 | 2.47 | 0.35 |
| **L-HDL** | 1.303 | 0.929 | 1.829 | 0.125 | 1.019 | 0.592 | 1.756 | 0.945 | 1.506 | 0.956 | 2.372 | 0.077 |

| | | | | | | | | | | | | |
|---|---|---|---|---|---|---|---|---|---|---|---|---|
| **H-LDL** | 0.677 | 0.297 | 1.545 | 0.353 | 0.743 | 0.267 | 2.07 | 0.571 | 0.604 | 0.148 | 2.463 | 0.48 |
| **rs6533497** | | | | | | | | | | | | |
| | **Total** | | | | **Men** | | | | **Women** | | | |
| **Clinical marker** | **OR** | **95% CI** | | ***p-value*** | **OR** | **95% CI** | | ***p-value*** | **OR** | **95% CI** | | ***p-value*** |
| **H-BMI** | 1.086 | 0.695 | 1.697 | 0.719 | 0.847 | 0.448 | 1.601 | 0.609 | 0.847 | 0.448 | 1.601 | 0.31 |
| **H- Waist** | 0.647 | 0.397 | 1.053 | 0.078 | 0.656 | 0.311 | 1.385 | 0.268 | 0.649 | 0.339 | 1.245 | 0.193 |
| **H-WHI** | 0.841 | 0.547 | 1.294 | 0.432 | 1.023 | 0.559 | 1.873 | 0.942 | 0.561 | 0.268 | 1.175 | 0.123 |
| **H-WHR** | 0.855 | 0.557 | 1.315 | 0.477 | 0.759 | 0.411 | 1.401 | 0.379 | 0.962 | 0.526 | 1.76 | 0.901 |
| **H-%BF** | 0.87 | 0.564 | 1.342 | 0.53 | 0.681 | 0.371 | 1.249 | 0.214 | 1.101 | 0.592 | 2.045 | 0.762 |
| **H-Insulin** | 1.13 | 0.612 | 2.086 | 0.697 | 1.497 | 0.691 | 3.241 | 0.306 | 0.646 | 0.213 | 1.962 | 0.44 |
| **H-Glucose** | 1.976 | 0.681 | 5.733 | 0.203 | 1.272 | 0.257 | 6.302 | 0.769 | 3.024 | 0.7 | 13.062 | 0.121 |
| **H-HOMA** | 1.164 | 0.63 | 2.148 | 0.629 | 1.245 | 0.569 | 2.728 | 0.585 | 0.975 | 0.355 | 2.681 | 0.961 |
| **H-Cholesterol** | 0.797 | 0.347 | 1.832 | 0.593 | 0.911 | 0.331 | 2.511 | 0.858 | 0.591 | 0.132 | 2.649 | 0.489 |
| **H-Triglycerides** | 1.111 | 0.666 | 1.855 | 0.687 | 1.226 | 0.628 | 2.391 | 0.552 | 0.937 | 0.411 | 2.133 | 0.876 |
| **L-HDL** | 1.149 | 0.746 | 1.771 | 0.529 | 1.714 | 0.9 | 3.266 | 0.099 | 0.875 | 0.482 | 1.588 | 0.662 |
| **H-LDL** | 0.798 | 0.27 | 2.357 | 0.683 | 0.262 | 0.034 | 2.021 | 0.169 | 2.143 | 0.536 | 8.57 | 0.272 |
| **rs77504516** | | | | | | | | | | | | |
| | **Total** | | | | **Men** | | | | **Women** | | | |
| **Clinical marker** | **OR** | **95% CI** | | ***p-value*** | **OR** | **95% CI** | | ***p-value*** | **OR** | **95% CI** | | ***p-value*** |
| **H-BMI** | 1.297 | 0.649 | 2.596 | 0.461 | 1.24 | 0.428 | 3.591 | 0.692 | 1.372 | 0.549 | 3.431 | 0.499 |
| **H- Waist** | 1.119 | 0.547 | 2.29 | 0.758 | 2.103 | 0.721 | 6.13 | 0.166 | 0.673 | 0.253 | 1.787 | 0.426 |
| **H-WHI** | 0.67 | 0.328 | 1.367 | 0.269 | 0.835 | 0.294 | 2.369 | 0.736 | 0.588 | 0.192 | 1.801 | 0.349 |
| **H-WHR** | 0.836 | 0.419 | 1.668 | 0.612 | 1.172 | 0.413 | 3.324 | 0.767 | 0.645 | 0.253 | 1.647 | 0.358 |
| **H-%BF** | 0.821 | 0.417 | 1.619 | 0.57 | 1.05 | 0.37 | 2.98 | 0.928 | 0.702 | 0.282 | 1.748 | 0.447 |
| **H-Insulin** | 0.682 | 0.231 | 2.014 | 0.487 | 1.014 | 0.267 | 3.856 | 0.983 | 0.369 | 0.047 | 2.885 | 0.325 |
| **H-Glucose** | 0.973 | 0.125 | 7.552 | 0.979 | 2.348 | 0.274 | 20.104 | 0.424 | 0.972 | 0.954 | 0.991 | 0.442 |
| **H-HOMA** | 0.815 | 0.279 | 2.382 | 0.708 | 1.279 | 0.342 | 4.786 | 0.715 | 0.426 | 0.055 | 3.299 | 0.402 |
| **H-Cholesterol** | 0.675 | 0.157 | 2.899 | 0.595 | 0.62 | 0.079 | 4.894 | 0.648 | 0.803 | 0.102 | 6.346 | 0.835 |
| **H-Triglycerides** | 1.559 | 0.73 | 3.328 | 0.248 | 1.553 | 0.512 | 4.707 | 0.435 | 1.701 | 0.593 | 4.878 | 0.319 |

| | | | | | | | | | | | | |
|---|---|---|---|---|---|---|---|---|---|---|---|---|
| **L-HDL** | 1.14 | 0.571 | 2.277 | 0.711 | 1.553 | 0.512 | 4.707 | 0.435 | 0.861 | 0.352 | 2.106 | 0.744 |
| **H-LDL** | 0.588 | 0.077 | 4.46 | 0.604 | 1.138 | 0.141 | 9.21 | 0.904 | 0.966 | 0.945 | 0.987 | 0.389 |
| | | | | | | **rs6815102** | | | | | | |
| | **Total** | | | | **Men** | | | | **Women** | | | |
| **Clinical marker** | **OR** | **95% CI** | | ***p*-value** | **OR** | **95% CI** | | ***p*-value** | **OR** | **95% CI** | | ***p*-value** |
| **H-BMI** | 1.095 | 0.771 | 1.555 | 0.615 | 0.918 | 0.558 | 1.51 | 0.736 | 1.284 | 0.781 | 2.111 | 0.326 |
| **H- Waist** | 0.797 | 0.561 | 1.133 | 0.206 | 0.71 | 0.413 | 1.22 | 0.215 | 0.894 | 0.56 | 1.428 | 0.641 |
| **H-WHI** | 1.046 | 0.751 | 1.455 | 0.791 | 1.083 | 0.672 | 1.747 | 0.744 | 0.916 | 0.555 | 1.512 | 0.732 |
| **H-WHR** | 1.085 | 0.779 | 1.511 | 0.63 | 1.114 | 0.689 | 1.8 | 0.661 | 1.06 | 0.67 | 1.677 | 0.804 |
| **H-%BF** | 1.141 | 0.817 | 1.593 | 0.44 | 1.076 | 0.664 | 1.743 | 0.769 | 1.182 | 0.744 | 1.88 | 0.481 |
| **H-Insulin** | 0.575 | 0.349 | 0.949 | 0.029 | 0.478 | 0.244 | 0.939 | 0.03 | 0.684 | 0.319 | 1.465 | 0.328 |
| **H-Glucose** | 0.716 | 0.265 | 1.934 | 0.509 | 0.524 | 0.138 | 1.994 | 0.337 | 1.035 | 0.228 | 4.706 | 0.964 |
| **H-HOMA** | 0.834 | 0.512 | 1.359 | 0.466 | 0.653 | 0.341 | 1.251 | 0.198 | 1.064 | 0.501 | 2.263 | 0.872 |
| **H-Cholesterol** | 1.179 | 0.639 | 2.174 | 0.598 | 1.105 | 0.501 | 2.436 | 0.805 | 1.234 | 0.465 | 3.273 | 0.673 |
| **H-Triglycerides** | 0.931 | 0.622 | 1.396 | 0.731 | 0.887 | 0.515 | 1.528 | 0.666 | 0.943 | 0.511 | 1.743 | 0.853 |
| **L-HDL** | 0.855 | 0.61 | 1.2 | 0.366 | 0.958 | 0.555 | 1.654 | 0.878 | 0.83 | 0.529 | 1.303 | 0.419 |
| **H-LDL** | 1.47 | 0.649 | 3.327 | 0.354 | 0.952 | 0.352 | 2.579 | 0.924 | 3.205 | 0.669 | 15.346 | 0.126 |
| | | | | | | **rs4326075** | | | | | | |
| | **Total** | | | | **Men** | | | | **Women** | | | |
| **Clinical marker** | **OR** | **95% CI** | | ***p*-value** | **OR** | **95% CI** | | ***p*-value** | **OR** | **95% CI** | | ***p*-value** |
| **H-BMI** | 0.962 | 0.676 | 1.368 | 0.83 | 0.867 | 0.526 | 1.427 | 0.575 | 1.055 | 0.641 | 1.738 | 0.834 |
| **H- Waist** | 0.92 | 0.644 | 1.314 | 0.646 | 0.64 | 0.365 | 1.121 | 0.118 | 1.244 | 0.774 | 1.999 | 0.369 |
| **H-WHI** | 1.283 | 0.921 | 1.789 | 0.141 | 1.133 | 0.703 | 1.827 | 0.61 | 1.405 | 0.847 | 2.331 | 0.188 |
| **H-WHR** | 1.157 | 0.83 | 1.614 | 0.391 | 1.117 | 0.694 | 1.798 | 0.649 | 1.197 | 0.751 | 1.908 | 0.451 |
| **H-%BF** | 1.199 | 0.857 | 1.678 | 0.289 | 1.235 | 0.763 | 2 | 0.392 | 1.151 | 0.719 | 1.841 | 0.559 |
| **H-Insulin** | 0.726 | 0.432 | 1.221 | 0.227 | 0.502 | 0.244 | 1.031 | 0.058 | 1.112 | 0.515 | 2.4 | 0.788 |
| **H-Glucose** | 0.312 | 0.089 | 1.099 | 0.056 | 0.385 | 0.079 | 1.887 | 0.224 | 0.224 | 0.027 | 1.844 | 0.13 |
| **H-HOMA** | 0.928 | 0.565 | 1.523 | 0.767 | 0.669 | 0.343 | 1.306 | 0.239 | 1.334 | 0.63 | 2.824 | 0.452 |
| **H-Cholesterol** | 1.35 | 0.743 | 2.456 | 0.324 | 1.139 | 0.526 | 2.467 | 0.742 | 1.664 | 0.641 | 4.318 | 0.293 |

| | | | | | | | | | | | | |
|---|---|---|---|---|---|---|---|---|---|---|---|---|
| H-Triglycerides | 0.687 | 0.452 | 1.046 | 0.08 | 0.633 | 0.361 | 1.109 | 0.109 | 0.722 | 0.379 | 1.374 | 0.321 |
| L-HDL | 0.552 | 0.312 | 0.977 | 0.042 | 0.552 | 0.312 | 0.977 | 0.04 | 0.844 | 0.533 | 1.336 | 0.47 |
| H-LDL | 1.651 | 0.762 | 3.578 | 0.2 | 1.601 | 0.599 | 4.279 | 0.346 | 1.64 | 0.465 | 5.79 | 0.439 |
| **rs116418972** | | | | | | | | | | | | |
| | **Total** | | | | **Men** | | | | **Women** | | | |
| **Clinical marker** | **OR** | **95% CI** | | ***p-value*** | **OR** | **95% CI** | | ***p-value*** | **OR** | **95% CI** | | ***p-value*** |
| H-BMI | 1.155 | 0.334 | 3.994 | 0.82 | 1.867 | 0.116 | 30.179 | 0.656 | 1.092 | 0.267 | 4.464 | 0.902 |
| H- Waist | 1.283 | 0.371 | 4.436 | 0.694 | 3.071 | 0.19 | 49.752 | 0.408 | 0.859 | 0.211 | 3.504 | 0.833 |
| H-WHI | 0.774 | 0.224 | 2.674 | 0.686 | 0.575 | 0.521 | 0.636 | 0.227 | 0.731 | 0.149 | 3.592 | 0.7 |
| H-WHR | 0.754 | 0.218 | 2.605 | 0.655 | 1.328 | 0.082 | 21.441 | 0.842 | 0.656 | 0.161 | 2.674 | 0.556 |
| H-%BF | 0.864 | 0.261 | 2.863 | 0.811 | 0.518 | 0.462 | 0.581 | 0.175 | 0.575 | 0.141 | 2.345 | 0.437 |
| H-Insulin | 0.815 | 0.779 | 0.853 | 0.246 | | | | | 0.858 | 0.813 | 0.905 | 0.322 |
| H-Glucose | 0.971 | 0.958 | 0.985 | 0.568 | 0.969 | 0.949 | 0.989 | 0.8 | 0.974 | 0.956 | 0.992 | 0.622 |
| H-HOMA | 0.859 | 0.83 | 0.889 | 0.201 | 0.822 | 0.776 | 0.871 | 0.513 | 0.891 | 0.856 | 0.928 | 0.326 |
| H-Cholesterol | 0.92 | 0.898 | 0.942 | 0.329 | 0.899 | 0.864 | 0.934 | 0.636 | 0.94 | 0.914 | 0.967 | 0.452 |
| H-Triglycerides | 0.793 | 0.76 | 0.826 | 0.091 | 0.748 | 0.7 | 0.8 | 0.414 | 0.834 | 0.794 | 0.877 | 0.184 |
| L-HDL | 2.16 | 0.651 | 7.162 | 0.198 | 3.028 | 0.187 | 49.044 | 0.414 | 1.466 | 0.386 | 5.565 | 0.574 |
| H-LDL | 0.954 | 0.937 | 0.971 | 0.468 | 0.941 | 0.914 | 0.968 | 0.723 | 0.967 | 0.947 | 0.987 | 0.58 |
| **rs11729740** | | | | | | | | | | | | |
| | **Total** | | | | **Men** | | | | **Women** | | | |
| **Clinical marker** | **OR** | **95% CI** | | ***p-value*** | **OR** | **95% CI** | | ***p-value*** | **OR** | **95% CI** | | ***p-value*** |
| H-BMI | 1.143 | 0.671 | 1.945 | 0.624 | 1.618 | 0.761 | 3.439 | 0.209 | 0.819 | 0.378 | 1.775 | 0.614 |
| H- Waist | 1.022 | 0.591 | 1.769 | 0.937 | 1.28 | 0.56 | 2.926 | 0.559 | 0.847 | 0.406 | 1.766 | 0.658 |
| H-WHI | 1.196 | 0.718 | 1.993 | 0.492 | 1.174 | 0.547 | 2.52 | 0.682 | 1.338 | 0.637 | 2.811 | 0.443 |
| H-WHR | 0.823 | 0.489 | 1.386 | 0.465 | 0.952 | 0.447 | 2.026 | 0.899 | 0.723 | 0.352 | 1.488 | 0.379 |
| H-%BF | 0.763 | 0.453 | 1.284 | 0.308 | 0.781 | 0.365 | 1.669 | 0.524 | 0.754 | 0.368 | 1.547 | 0.442 |
| H-Insulin | 1.175 | 0.559 | 2.471 | 0.671 | 1.782 | 0.673 | 4.721 | 0.242 | 0.72 | 0.204 | 2.545 | 0.611 |
| H-Glucose | 1.061 | 0.237 | 4.742 | 0.939 | 1.038 | 0.125 | 8.584 | 0.973 | 1.094 | 0.131 | 9.156 | 0.934 |
| H-HOMA | 0.971 | 0.442 | 2.136 | 0.942 | 1.257 | 0.443 | 3.564 | 0.669 | 0.77 | 0.221 | 2.679 | 0.682 |

| | | | | | | | | | | | | |
|---|---|---|---|---|---|---|---|---|---|---|---|---|
| **H-Cholesterol** | 0.722 | 0.251 | 2.079 | 0.545 | 0.952 | 0.271 | 3.349 | 0.939 | 0.434 | 0.056 | 3.36 | 0.412 |
| **H-Triglycerides** | 1.146 | 0.621 | 2.114 | 0.664 | 1.26 | 0.552 | 2.877 | 0.585 | 1.05 | 0.413 | 2.672 | 0.919 |
| **L-HDL** | 1.063 | 0.628 | 1.798 | 0.821 | 1.049 | 0.447 | 2.461 | 0.913 | 1.043 | 0.52 | 2.091 | 0.907 |
| **H-LDL** | 0.624 | 0.144 | 2.696 | 0.525 | 1.113 | 0.242 | 5.112 | 0.891 | 0.964 | 0.942 | 0.986 | 0.246 |
| | | | | | | **rs2035415** | | | | | | |
| | **Total** | | | | **Men** | | | | **Women** | | | |
| **Clinical marker** | **OR** | **95% CI** | | ***p-value*** | **OR** | **95% CI** | | ***p-value*** | **OR** | **95% CI** | | ***p-value*** |
| **H-BMI** | 1.032 | 0.701 | 1.522 | 0.872 | 0.93 | 0.528 | 1.638 | 0.803 | 1.11 | 0.65 | 1.895 | 0.705 |
| **H- Waist** | 0.797 | 0.543 | 1.17 | 0.247 | 0.868 | 0.471 | 1.599 | 0.65 | 0.792 | 0.479 | 1.31 | 0.365 |
| **H-WHI** | 0.924 | 0.642 | 1.331 | 0.672 | 0.931 | 0.54 | 1.604 | 0.797 | 0.766 | 0.449 | 1.308 | 0.331 |
| **H-WHR** | 1.068 | 0.74 | 1.542 | 0.725 | 1.178 | 0.68 | 2.038 | 0.56 | 0.986 | 0.6 | 1.621 | 0.956 |
| **H-%BF** | 0.97 | 0.671 | 1.403 | 0.872 | 1.078 | 0.622 | 1.87 | 0.789 | 0.861 | 0.522 | 1.42 | 0.56 |
| **H-Insulin** | 0.753 | 0.436 | 1.3 | 0.308 | 0.678 | 0.326 | 1.411 | 0.299 | 0.832 | 0.359 | 1.928 | 0.669 |
| **H-Glucose** | 1.214 | 0.39 | 3.779 | 0.738 | 2.679 | 0.329 | 21.802 | 0.34 | 0.685 | 0.16 | 2.93 | 0.61 |
| **H-HOMA** | 0.946 | 0.55 | 1.628 | 0.842 | 0.674 | 0.332 | 1.367 | 0.274 | 1.408 | 0.582 | 3.407 | 0.447 |
| **H-Cholesterol** | 1.576 | 0.743 | 3.344 | 0.233 | 1.032 | 0.421 | 2.527 | 0.946 | 3.272 | 0.733 | 14.61 | 0.102 |
| **H-Triglycerides** | 1.003 | 0.641 | 1.569 | 0.991 | 0.804 | 0.439 | 1.47 | 0.479 | 1.22 | 0.615 | 2.421 | 0.571 |
| **L-HDL** | 1.019 | 0.7 | 1.484 | 0.922 | 1.196 | 0.634 | 2.255 | 0.582 | 1.017 | 0.623 | 1.661 | 0.946 |
| **H-LDL** | 1.316 | 0.521 | 3.32 | 0.561 | 0.773 | 0.263 | 2.274 | 0.64 | 3.857 | 0.482 | 30.896 | 0.173 |
| | | | | | | **rs17041402** | | | | | | |
| | **Total** | | | | **Men** | | | | **Women** | | | |
| **Clinical marker** | **OR** | **95% CI** | | ***p-value*** | **OR** | **95% CI** | | ***p-value*** | **OR** | **95% CI** | | ***p-value*** |
| **H-BMI** | 1.426 | 0.534 | 3.806 | 0.477 | 0.616 | 0.063 | 5.998 | 0.675 | 1.926 | 0.629 | 5.895 | 0.245 |
| **H- Waist** | 1.226 | 0.446 | 3.366 | 0.693 | 1.014 | 0.104 | 9.909 | 0.99 | 1.082 | 0.345 | 3.391 | 0.893 |
| **H-WHI** | 0.558 | 0.194 | 1.606 | 0.274 | 0.726 | 0.101 | 5.225 | 0.75 | 0.766 | 0.206 | 2.854 | 0.692 |
| **H-WHR** | 0.927 | 0.348 | 2.469 | 0.879 | 0.437 | 0.045 | 4.254 | 0.466 | 1.145 | 0.376 | 3.491 | 0.812 |
| **H-%BF** | 0.922 | 0.35 | 2.423 | 0.869 | 0.915 | 0.127 | 6.593 | 0.93 | 1.002 | 0.329 | 3.057 | 0.997 |
| **H-Insulin** | 0.814 | 0.777 | 0.852 | 0.132 | 0.766 | 0.71 | 0.828 | 0.438 | 0.856 | 0.811 | 0.905 | 0.25 |
| **H-Glucose** | 0.971 | 0.957 | 0.985 | 0.476 | 0.968 | 0.948 | 0.989 | 0.719 | 0.973 | 0.955 | 0.992 | 0.551 |

| | | | | | | | | | | | | |
|---|---|---|---|---|---|---|---|---|---|---|---|---|
| H-HOMA | 0.857 | 0.828 | 0.887 | 0.104 | 0.821 | 0.775 | 0.87 | 0.353 | 0.89 | 0.854 | 0.927 | 0.225 |
| H-Cholesterol | 0.919 | 0.897 | 0.942 | 0.223 | 0.898 | 0.863 | 0.934 | 0.502 | 0.94 | 0.913 | 0.967 | 0.363 |
| H-Triglycerides | 0.79 | 0.758 | 0.824 | 0.034 | 0.746 | 0.698 | 0.799 | 0.246 | 0.832 | 0.791 | 0.876 | 0.108 |
| L-HDL | 2.607 | 0.978 | 6.952 | 0.047 | 9.348 | 0.957 | 91.332 | 0.02 | 1.371 | 0.45 | 4.177 | 0.579 |
| H-LDL | 0.954 | 0.937 | 0.971 | 0.364 | 0.94 | 0.913 | 0.968 | 0.615 | 0.966 | 0.946 | 0.987 | 0.504 |
| **rs59111930** | | | | | | | | | | | | |
| | **Total** | | | | **Men** | | | | **Women** | | | |
| **Clinical marker** | **OR** | **95% CI** | | ***p*-value** | **OR** | **95% CI** | | ***p*-value** | **OR** | **95% CI** | | ***p*-value** |
| H-BMI | 0.753 | 0.533 | 1.064 | 0.108 | 0.718 | 0.439 | 1.174 | 0.187 | 0.77 | 0.472 | 1.256 | 0.295 |
| H- Waist | 0.806 | 0.569 | 1.142 | 0.226 | 0.656 | 0.383 | 1.125 | 0.125 | 0.995 | 0.625 | 1.584 | 0.983 |
| H-WHI | 1.03 | 0.743 | 1.426 | 0.86 | 0.863 | 0.538 | 1.384 | 0.542 | 1.046 | 0.636 | 1.722 | 0.859 |
| H-WHR | 0.933 | 0.673 | 1.293 | 0.677 | 0.954 | 0.596 | 1.529 | 0.846 | 0.912 | 0.579 | 1.437 | 0.692 |
| H-%BF | 0.91 | 0.655 | 1.265 | 0.577 | 0.95 | 0.59 | 1.529 | 0.833 | 0.846 | 0.535 | 1.34 | 0.478 |
| H-Insulin | 0.898 | 0.548 | 1.472 | 0.671 | 0.672 | 0.347 | 1.303 | 0.24 | 1.301 | 0.604 | 2.802 | 0.503 |
| H-Glucose | 0.902 | 0.334 | 2.436 | 0.839 | 0.986 | 0.259 | 3.748 | 0.983 | 0.765 | 0.168 | 3.476 | 0.729 |
| H-HOMA | 0.999 | 0.615 | 1.623 | 0.997 | 0.656 | 0.344 | 1.252 | 0.201 | 1.645 | 0.768 | 3.526 | 0.199 |
| H-Cholesterol | 1.504 | 0.816 | 2.771 | 0.189 | 0.968 | 0.447 | 2.094 | 0.934 | 2.823 | 0.981 | 8.121 | 0.046 |
| H-Triglycerides | 0.717 | 0.481 | 1.069 | 0.102 | 0.5 | 0.291 | 0.859 | 0.011 | 1.031 | 0.563 | 1.889 | 0.921 |
| L-HDL | 0.717 | 0.513 | 1.003 | 0.052 | 0.582 | 0.34 | 0.995 | 0.047 | 0.898 | 0.575 | 1.404 | 0.638 |
| H-LDL | 1.333 | 0.608 | 2.922 | 0.473 | 1.135 | 0.42 | 3.071 | 0.803 | 1.561 | 0.432 | 5.645 | 0.495 |
| **rs74874270** | | | | | | | | | | | | |
| | **Total** | | | | **Men** | | | | **Women** | | | |
| **Clinical marker** | **OR** | **95% CI** | | ***p*-value** | **OR** | **95% CI** | | ***p*-value** | **OR** | **95% CI** | | ***p*-value** |
| H-BMI | 0.387 | 0.146 | 1.028 | 0.049 | 0.271 | 0.06 | 1.227 | 0.071 | 0.53 | 0.146 | 1.923 | 0.329 |
| H- Waist | 0.844 | 0.367 | 1.943 | 0.691 | 0.453 | 0.1 | 2.056 | 0.295 | 1.311 | 0.443 | 3.878 | 0.625 |
| H-WHI | 0.705 | 0.322 | 1.543 | 0.38 | 0.622 | 0.219 | 1.764 | 0.37 | 0.693 | 0.189 | 2.548 | 0.581 |
| H-WHR | 0.686 | 0.313 | 1.502 | 0.344 | 0.464 | 0.144 | 1.495 | 0.19 | 0.996 | 0.337 | 2.944 | 0.994 |
| H-%BF | 0.837 | 0.395 | 1.773 | 0.642 | 0.792 | 0.279 | 2.248 | 0.662 | 0.871 | 0.295 | 2.576 | 0.804 |
| H-Insulin | 1.525 | 0.478 | 4.863 | 0.474 | 0.828 | 0.09 | 7.595 | 0.868 | 2.478 | 0.62 | 9.899 | 0.187 |

| | | | | | | | | | | | | |
|---|---|---|---|---|---|---|---|---|---|---|---|---|
| **H-Glucose** | 1.192 | 0.153 | 9.3 | 0.867 | 2.366 | 0.276 | 20.256 | 0.42 | 0.973 | 0.955 | 0.992 | 0.53 |
| **H-HOMA** | 1.256 | 0.417 | 3.779 | 0.686 | 0.455 | 0.057 | 3.643 | 0.449 | 2.7 | 0.701 | 10.394 | 0.135 |
| **H-Cholesterol** | 0.917 | 0.895 | 0.94 | 0.101 | 0.894 | 0.858 | 0.931 | 0.184 | 0.939 | 0.912 | 0.967 | 0.327 |
| **H-Triglycerides** | 0.267 | 0.063 | 1.138 | 0.056 | 0.203 | 0.026 | 1.573 | 0.093 | 0.36 | 0.046 | 2.802 | 0.311 |
| **L-HDL** | 1.379 | 0.656 | 2.896 | 0.395 | 1.096 | 0.338 | 3.556 | 0.879 | 1.789 | 0.621 | 5.153 | 0.277 |
| **H-LDL** | 0.953 | 0.935 | 0.97 | 0.223 | 0.938 | 0.909 | 0.967 | 0.321 | 0.966 | 0.946 | 0.987 | 0.471 |

| **rs1384331** | | | | | | | | | | | | |
|---|---|---|---|---|---|---|---|---|---|---|---|---|
| | **Total** | | | | **Men** | | | | **Women** | | | |
| **Clinical marker** | **OR** | **95% CI** | | ***p-value*** | **OR** | **95% CI** | | ***p-value*** | **OR** | **95% CI** | | ***p-value*** |
| **H-BMI** | 0.951 | 0.663 | 1.365 | 0.787 | 0.885 | 0.529 | 1.48 | 0.642 | 1.01 | 0.608 | 1.678 | 0.968 |
| **H- Waist** | 0.878 | 0.611 | 1.262 | 0.483 | 0.92 | 0.524 | 1.616 | 0.773 | 0.866 | 0.535 | 1.401 | 0.559 |
| **H-WHI** | 0.912 | 0.649 | 1.283 | 0.598 | 0.886 | 0.539 | 1.456 | 0.633 | 0.865 | 0.517 | 1.448 | 0.582 |
| **H-WHR** | 1.076 | 0.764 | 1.516 | 0.675 | 1.158 | 0.704 | 1.905 | 0.565 | 1.007 | 0.627 | 1.618 | 0.976 |
| **H-%BF** | 1.003 | 0.71 | 1.416 | 0.988 | 0.937 | 0.567 | 1.546 | 0.799 | 1.051 | 0.652 | 1.696 | 0.838 |
| **H-Insulin** | 0.803 | 0.481 | 1.341 | 0.402 | 0.711 | 0.359 | 1.409 | 0.33 | 0.923 | 0.417 | 2.043 | 0.845 |
| **H-Glucose** | 1.283 | 0.446 | 3.691 | 0.644 | 4.197 | 0.517 | 34.048 | 0.147 | 0.544 | 0.133 | 2.217 | 0.39 |
| **H-HOMA** | 0.937 | 0.564 | 1.555 | 0.801 | 0.688 | 0.354 | 1.335 | 0.269 | 1.384 | 0.612 | 3.13 | 0.435 |
| **H-Cholesterol** | 1.379 | 0.709 | 2.683 | 0.342 | 0.961 | 0.428 | 2.156 | 0.924 | 2.699 | 0.758 | 9.606 | 0.113 |
| **H-Triglycerides** | 0.871 | 0.576 | 1.319 | 0.516 | 0.63 | 0.363 | 1.091 | 0.098 | 1.305 | 0.676 | 2.52 | 0.428 |
| **L-HDL** | 1.197 | 0.84 | 1.706 | 0.321 | 1.44 | 0.802 | 2.588 | 0.222 | 1.114 | 0.698 | 1.779 | 0.651 |
| **H-LDL** | 0.898 | 0.403 | 1.998 | 0.792 | 1.234 | 0.422 | 3.607 | 0.702 | 0.541 | 0.153 | 1.912 | 0.335 |

| **rs72679246** | | | | | | | | | | | | |
|---|---|---|---|---|---|---|---|---|---|---|---|---|
| | **Total** | | | | **Men** | | | | **Women** | | | |
| **Clinical marker** | **OR** | **95% CI** | | ***p-value*** | **OR** | **95% CI** | | ***p-value*** | **OR** | **95% CI** | | ***p-value*** |
| **H-BMI** | 0.333 | 0.074 | 1.505 | 0.134 | 0.745 | 0.142 | 3.912 | 0.728 | 0.682 | 0.63 | 0.738 | 0.073 |
| **H- Waist** | 0.364 | 0.081 | 1.645 | 0.172 | 0.512 | 0.061 | 4.333 | 0.534 | 0.274 | 0.033 | 2.308 | 0.205 |
| **H-WHI** | 0.761 | 0.252 | 2.3 | 0.628 | 0.563 | 0.124 | 2.562 | 0.453 | 1.02 | 0.194 | 5.36 | 0.982 |
| **H-WHR** | 0.541 | 0.168 | 1.746 | 0.298 | 0.546 | 0.104 | 2.865 | 0.47 | 0.537 | 0.102 | 2.812 | 0.456 |
| **H-%BF** | 0.415 | 0.129 | 1.34 | 0.13 | 0.706 | 0.155 | 3.214 | 0.652 | 0.19 | 0.023 | 1.595 | 0.089 |

| | | | | | | | | | | | | |
|---|---|---|---|---|---|---|---|---|---|---|---|---|
| **H-Insulin** | 0.82 | 0.783 | 0.858 | 0.106 | 0.772 | 0.715 | 0.835 | 0.188 | 0.862 | 0.816 | 0.91 | 0.33 |
| **H-Glucose** | 6.144 | 1.263 | 29.902 | 0.011 | 0.967 | 0.946 | 0.988 | 0.627 | 19.2 | 3.085 | 119.485 | 0.001 |
| **H-HOMA** | 0.86 | 0.83 | 0.89 | 0.132 | 0.823 | 0.776 | 0.872 | 0.223 | 0.892 | 0.856 | 0.929 | 0.359 |
| **H-Cholesterol** | 0.873 | 0.112 | 6.823 | 0.897 | 1.464 | 0.17 | 12.606 | 0.728 | 0.939 | 0.912 | 0.967 | 0.501 |
| **H-Triglycerides** | 0.287 | 0.037 | 2.217 | 0.203 | 0.486 | 0.057 | 4.105 | 0.5 | 0.83 | 0.788 | 0.874 | 0.234 |
| **L-HDL** | 1.369 | 0.469 | 4.002 | 0.565 | 2.364 | 0.516 | 10.832 | 0.256 | 0.883 | 0.194 | 4.016 | 0.873 |
| **H-LDL** | 0.954 | 0.937 | 0.972 | 0.414 | 0.938 | 0.91 | 0.967 | 0.498 | 0.969 | 0.95 | 0.989 | 0.64 |
| | | | | | | | **rs78563565** | | | | | |
| | **Total** | | | | **Men** | | | | **Women** | | | |
| **Clinical marker** | **OR** | **95% CI** | | ***p*-value** | **OR** | **95% CI** | | ***p*-value** | **OR** | **95% CI** | | ***p*-value** |
| **H-BMI** | 1.274 | 0.567 | 2.862 | 0.558 | 0.926 | 0.272 | 3.156 | 0.903 | 1.677 | 0.565 | 4.976 | 0.349 |
| **H- Waist** | 0.816 | 0.337 | 1.976 | 0.652 | 0.597 | 0.128 | 2.792 | 0.51 | 0.957 | 0.313 | 2.928 | 0.939 |
| **H-WHI** | 0.845 | 0.377 | 1.893 | 0.682 | 0.719 | 0.226 | 2.285 | 0.576 | 1.034 | 0.315 | 3.39 | 0.956 |
| **H-WHR** | 0.822 | 0.367 | 1.844 | 0.635 | 0.944 | 0.292 | 3.05 | 0.924 | 0.728 | 0.238 | 2.224 | 0.577 |
| **H-%BF** | 0.681 | 0.301 | 1.544 | 0.356 | 0.443 | 0.13 | 1.507 | 0.183 | 1.002 | 0.329 | 3.057 | 0.997 |
| **H-Insulin** | 0.998 | 0.328 | 3.038 | 0.998 | 1.455 | 0.361 | 5.869 | 0.598 | 0.552 | 0.069 | 4.429 | 0.572 |
| **H-Glucose** | 0.97 | 0.957 | 0.984 | 0.374 | 0.967 | 0.947 | 0.989 | 0.527 | 0.973 | 0.955 | 0.992 | 0.535 |
| **H-HOMA** | 0.802 | 0.235 | 2.74 | 0.725 | 0.93 | 0.197 | 4.398 | 0.928 | 0.638 | 0.081 | 5.054 | 0.67 |
| **H-Cholesterol** | 0.458 | 0.061 | 3.459 | 0.439 | 0.805 | 0.1 | 6.471 | 0.839 | 0.939 | 0.913 | 0.967 | 0.344 |
| **H-Triglycerides** | 1.789 | 0.759 | 4.219 | 0.179 | 2.228 | 0.685 | 7.252 | 0.174 | 1.451 | 0.39 | 5.398 | 0.578 |
| **L-HDL** | 1.55 | 0.704 | 3.415 | 0.274 | 1.529 | 0.447 | 5.238 | 0.498 | 1.578 | 0.535 | 4.661 | 0.407 |
| **H-LDL** | 0.842 | 0.11 | 6.453 | 0.868 | 1.477 | 0.179 | 12.166 | 0.716 | 0.966 | 0.946 | 0.987 | 0.487 |
| | | | | | | | **rs6533498** | | | | | |
| | **Total** | | | | **Men** | | | | **Women** | | | |
| **Clinical marker** | **OR** | **95% CI** | | ***p*-value** | **OR** | **95% CI** | | ***p*-value** | **OR** | **95% CI** | | ***p*-value** |
| **H-BMI** | 1.039 | 0.735 | 1.468 | 0.83 | 0.788 | 0.483 | 1.286 | 0.341 | 1.37 | 0.837 | 2.242 | 0.211 |
| **H- Waist** | 1.074 | 0.758 | 1.524 | 0.688 | 0.749 | 0.438 | 1.282 | 0.293 | 1.426 | 0.893 | 2.279 | 0.138 |
| **H-WHI** | 1.08 | 0.78 | 1.497 | 0.644 | 1.029 | 0.644 | 1.646 | 0.904 | 1.131 | 0.686 | 1.865 | 0.63 |
| **H-WHR** | 1.111 | 0.801 | 1.54 | 0.529 | 0.924 | 0.578 | 1.478 | 0.744 | 1.321 | 0.837 | 2.087 | 0.233 |

| | | | | | | | | | | | | |
|---|---|---|---|---|---|---|---|---|---|---|---|---|
| **H-%BF** | 1.157 | 0.833 | 1.609 | 0.385 | 0.906 | 0.564 | 1.454 | 0.683 | 1.457 | 0.918 | 2.313 | 0.11 |
| **H-Insulin** | 0.768 | 0.468 | 1.261 | 0.297 | 0.794 | 0.41 | 1.536 | 0.495 | 0.727 | 0.339 | 1.557 | 0.412 |
| **H-Glucose** | 0.902 | 0.334 | 2.436 | 0.839 | 0.698 | 0.184 | 2.654 | 0.597 | 1.24 | 0.273 | 5.634 | 0.781 |
| **H-HOMA** | 0.907 | 0.559 | 1.472 | 0.693 | 0.95 | 0.499 | 1.81 | 0.878 | 0.849 | 0.403 | 1.788 | 0.667 |
| **H-Cholesterol** | 0.711 | 0.391 | 1.294 | 0.264 | 0.591 | 0.271 | 1.287 | 0.183 | 0.921 | 0.355 | 2.386 | 0.866 |
| **H-Triglycerides** | 0.998 | 0.67 | 1.486 | 0.99 | 1.228 | 0.718 | 2.102 | 0.455 | 0.753 | 0.41 | 1.382 | 0.361 |
| **L-HDL** | 1.11 | 0.794 | 1.551 | 0.542 | 1.324 | 0.772 | 2.271 | 0.308 | 1.011 | 0.647 | 1.581 | 0.961 |
| **H-LDL** | 0.713 | 0.328 | 1.549 | 0.391 | 0.46 | 0.165 | 1.28 | 0.13 | 1.403 | 0.388 | 5.073 | 0.605 |
| | | | | | | | **rs 9997926** | | | | | |
| | | **Total** | | | | **Men** | | | | **Women** | | |
| **Clinical marker** | **OR** | **95% CI** | | ***p-value*** | **OR** | **95% CI** | | ***p-value*** | **OR** | **95% CI** | | ***p-value*** |
| **H-BMI** | 1.168 | 0.636 | 2.146 | 0.617 | 1.213 | 0.544 | 2.704 | 0.637 | 1.075 | 0.419 | 2.757 | 0.881 |
| **H- Waist** | 1.279 | 0.698 | 2.345 | 0.426 | 1.49 | 0.641 | 3.461 | 0.353 | 1.208 | 0.499 | 2.925 | 0.676 |
| **H-WHI** | 1.975 | 1.098 | 3.553 | 0.021 | 2.354 | 0.967 | 5.734 | 0.054 | 1.506 | 0.608 | 3.733 | 0.375 |
| **H-WHR** | 0.947 | 0.526 | 1.703 | 0.855 | 1.15 | 0.526 | 2.516 | 0.727 | 0.737 | 0.299 | 1.813 | 0.506 |
| **H-%BF** | 1.242 | 0.678 | 2.275 | 0.483 | 1.162 | 0.523 | 2.584 | 0.714 | 1.289 | 0.508 | 3.272 | 0.594 |
| **H-Insulin** | 2.1 | 0.985 | 4.478 | 0.051 | 5.714 | 2.035 | 16.048 | 0.001 | 0.317 | 0.041 | 2.462 | 0.249 |
| **H-Glucose** | 2.344 | 0.651 | 8.442 | 0.181 | 2.758 | 0.544 | 13.973 | 0.204 | 1.779 | 0.209 | 15.12 | 0.594 |
| **H-HOMA** | 1.361 | 0.608 | 3.047 | 0.453 | 2.211 | 0.852 | 5.738 | 0.097 | 0.375 | 0.049 | 2.886 | 0.329 |
| **H-Cholesterol** | 1.32 | 0.497 | 3.505 | 0.577 | 1.553 | 0.499 | 4.838 | 0.446 | 0.753 | 0.095 | 5.946 | 0.788 |
| **H-Triglycerides** | 1.06 | 0.527 | 2.134 | 0.87 | 1.466 | 0.631 | 3.404 | 0.373 | 0.462 | 0.105 | 2.038 | 0.298 |
| **L-HDL** | 0.962 | 0.528 | 1.754 | 0.9 | 1.213 | 0.509 | 2.886 | 0.664 | 0.881 | 0.374 | 2.075 | 0.772 |
| **H-LDL** | 2.554 | 0.924 | 7.061 | 0.062 | 3.141 | 0.949 | 10.392 | 0.05 | 1.374 | 0.166 | 11.344 | 0.768 |
| | | | | | | | **rs 6824447** | | | | | |
| | | **Total** | | | | **Men** | | | | **Women** | | |
| **Clinical marker** | **OR** | **95% CI** | | ***p-value*** | **OR** | **95% CI** | | ***p-value*** | **OR** | **95% CI** | | ***p-value*** |
| **H-BMI** | 0.875 | 0.616 | 1.242 | 0.455 | 0.516 | 0.313 | 0.85 | 0.009 | 1.509 | 0.908 | 2.508 | 0.112 |
| **H- Waist** | 0.943 | 0.663 | 1.343 | 0.747 | 0.551 | 0.319 | 0.952 | 0.031 | 1.389 | 0.861 | 2.242 | 0.179 |
| **H-WHI** | 0.879 | 0.631 | 1.225 | 0.447 | 0.905 | 0.561 | 1.46 | 0.684 | 0.881 | 0.531 | 1.462 | 0.624 |

| | | | | | | | | | | | | |
|---|---|---|---|---|---|---|---|---|---|---|---|---|
| **H-WHR** | 0.936 | 0.671 | 1.305 | 0.696 | 0.711 | 0.441 | 1.145 | 0.161 | 1.216 | 0.762 | 1.939 | 0.413 |
| **H-%BF** | 1.097 | 0.783 | 1.535 | 0.592 | 0.881 | 0.544 | 1.426 | 0.608 | 1.369 | 0.853 | 2.199 | 0.194 |
| **H-Insulin** | 0.755 | 0.459 | 1.243 | 0.27 | 0.815 | 0.418 | 1.587 | 0.549 | 0.73 | 0.341 | 1.565 | 0.42 |
| **H-Glucose** | 0.988 | 0.363 | 2.691 | 0.982 | 0.632 | 0.166 | 2.403 | 0.499 | 1.875 | 0.358 | 9.822 | 0.452 |
| **H-HOMA** | 0.829 | 0.508 | 1.352 | 0.453 | 0.814 | 0.424 | 1.563 | 0.538 | 0.884 | 0.418 | 1.871 | 0.748 |
| **H-Cholesterol** | 0.447 | 0.242 | 0.825 | 0.008 | 0.268 | 0.114 | 0.628 | 0.001 | 0.92 | 0.353 | 2.401 | 0.866 |
| **H-Triglycerides** | 0.83 | 0.556 | 1.241 | 0.365 | 1.046 | 0.608 | 1.798 | 0.872 | 0.632 | 0.344 | 1.162 | 0.139 |
| **L-HDL** | 1.049 | 0.746 | 1.475 | 0.783 | 1.046 | 0.608 | 1.798 | 0.872 | 1.021 | 0.646 | 1.614 | 0.929 |
| **H-LDL** | 0.435 | 0.196 | 0.968 | 0.036 | 0.227 | 0.072 | 0.714 | 0.006 | 1.114 | 0.308 | 4.031 | 0.87 |

| **rs 17041272** | | | | | | | | | | | | |
|---|---|---|---|---|---|---|---|---|---|---|---|---|
| | **Total** | | | | **Men** | | | | **Women** | | | |
| **Clinical marker** | **OR** | **95% CI** | | ***p-value*** | **OR** | **95% CI** | | ***p-value*** | **OR** | **95% CI** | | ***p-value*** |
| **H-BMI** | 1.569 | 1 | 2.46 | 0.049 | 2.068 | 1.118 | 3.826 | 0.019 | 1.119 | 0.568 | 2.202 | 0.747 |
| **H- Waist** | 1.112 | 0.699 | 1.769 | 0.655 | 2.131 | 1.116 | 4.069 | 0.02 | 0.622 | 0.313 | 1.24 | 0.176 |
| **H-WHI** | 1.087 | 0.701 | 1.686 | 0.709 | 1.417 | 0.755 | 2.659 | 0.279 | 0.665 | 0.314 | 1.408 | 0.286 |
| **H-WHR** | 1.165 | 0.752 | 1.804 | 0.495 | 1.634 | 0.889 | 3.004 | 0.113 | 0.808 | 0.425 | 1.535 | 0.516 |
| **H-%BF** | 1.794 | 1.133 | 2.839 | 0.012 | 1.933 | 1.016 | 3.68 | 0.043 | 1.619 | 0.837 | 3.134 | 0.151 |
| **H-Insulin** | 2.147 | 1.198 | 3.847 | 0.009 | 2.101 | 0.967 | 4.564 | 0.058 | 2.113 | 0.858 | 5.205 | 0.099 |
| **H-Glucose** | 1.097 | 0.309 | 3.89 | 0.887 | 2.385 | 0.576 | 9.873 | 0.218 | 0.969 | 0.948 | 0.99 | 0.229 |
| **H-HOMA** | 1.956 | 1.096 | 3.491 | 0.021 | 2 | 0.935 | 4.279 | 0.071 | 1.807 | 0.725 | 4.5 | 0.2 |
| **H-Cholesterol** | 1.631 | 0.799 | 3.328 | 0.76 | 0.956 | 0.346 | 2.637 | 0.93 | 3.1 | 1.101 | 8.73 | 0.025 |
| **H-Triglycerides** | 1.522 | 0.922 | 2.513 | 0.099 | 1.155 | 0.583 | 2.286 | 0.681 | 2.062 | 0.981 | 4.333 | 0.053 |
| **L-HDL** | 1.219 | 0.781 | 1.903 | 0.385 | 1.155 | 0.583 | 2.286 | 0.681 | 1.433 | 0.763 | 2.688 | 0.263 |
| **H-LDL** | 1.488 | 0.584 | 3.789 | 0.402 | 0.987 | 0.273 | 3.568 | 0.984 | 2.522 | 0.628 | 10.13 | 0.179 |

**ELOVL7**

| **rs75621404** | | | | | | | | | | | | |
|---|---|---|---|---|---|---|---|---|---|---|---|---|
| | **Total** | | | | **Men** | | | | **Women** | | | |
| **Clinical marker** | **OR** | **95% CI** | | ***p-value*** | **OR** | **95% CI** | | ***p-value*** | **OR** | **95% CI** | | ***p-value*** |
| **H-BMI** | 1.129 | 0.373 | 3.415 | 0.83 | 1.925 | 0.543 | 6.817 | 0.304 | 0.681 | 0.63 | 0.736 | 0.174 |

| | | | | | | | | | | | | |
|---|---|---|---|---|---|---|---|---|---|---|---|---|
| **H- Waist** | 0.367 | 0.081 | 1.659 | 0.176 | 0.333 | 0.041 | 2.679 | 0.38 | 0.571 | 0.059 | 5.56 | 0.627 |
| **H-WHI** | 1.849 | 0.633 | 5.398 | 0.255 | 1.743 | 0.441 | 6.881 | 0.424 | 0.859 | 0.088 | 8.371 | 0.896 |
| **H-WHR** | 1.801 | 0.617 | 5.259 | 0.126 | 1.35 | 0.382 | 4.772 | 0.641 | 4.055 | 0.417 | 39.431 | 0.194 |
| **H-%BF** | 1.222 | 0.406 | 3.683 | 0.721 | 0.92 | 0.26 | 3.254 | 0.898 | 2.358 | 0.211 | 26.295 | 0.474 |
| **H-Insulin** | 0.823 | 0.179 | 3.792 | 0.803 | 0.97 | 0.194 | 4.843 | 0.971 | 0.859 | 0.814 | 0.906 | 0.421 |
| **H-Glucose** | 0.971 | 0.957 | 0.985 | 0.518 | 0.968 | 0.947 | 0.989 | 0.564 | 0.974 | 0.956 | 0.992 | 0.745 |
| **H-HOMA** | 0.477 | 0.062 | 3.702 | 0.47 | 0.519 | 0.064 | 4.208 | 0.534 | 0.893 | 0.858 | 0.929 | 0.49 |
| **H-Cholesterol** | 0.9 | 0.115 | 7.031 | 0.92 | 0.988 | 0.121 | 8.09 | 0.991 | 0.941 | 0.915 | 0.968 | 0.619 |
| **H-Triglycerides** | 1.597 | 0.492 | 5.181 | 0.433 | 1.317 | 0.331 | 5.235 | 0.696 | 1.755 | 0.179 | 17.222 | 0.626 |
| **L-HDL** | 0.982 | 0.325 | 2.969 | 0.975 | 0.739 | 0.153 | 3.564 | 0.707 | 3.532 | 0.363 | 34.333 | 0.248 |
| **H-LDL** | 1.651 | 0.208 | 13.104 | 0.632 | 1.813 | 0.216 | 15.203 | 0.58 | 0.967 | 0.948 | 0.987 | 0.715 |
| | | | | | | **rs1563517** | | | | | | |
| | | **Total** | | | | **Men** | | | | **Women** | | |
| **Clinical marker** | **OR** | **95% CI** | | ***p-value*** | **OR** | **95% CI** | | ***p-value*** | **OR** | **95% CI** | | ***p-value*** |
| **H-BMI** | 1.092 | 0.752 | 1.585 | 0.646 | 1.095 | 0.641 | 1.871 | 0.74 | 1.099 | 0.653 | 1.852 | 0.723 |
| **H- Waist** | 1.517 | 1.049 | 2.194 | 0.027 | 1.238 | 0.693 | 2.21 | 0.472 | 1.716 | 1.051 | 2.801 | 0.03 |
| **H-WHI** | 1.213 | 0.853 | 1.724 | 0.283 | 0.94 | 0.561 | 1.575 | 0.816 | 1.806 | 1.075 | 3.033 | 0.025 |
| **H-WHR** | 1.132 | 0.795 | 1.613 | 0.492 | 1.119 | 0.667 | 1.875 | 0.672 | 1.145 | 0.704 | 1.861 | 0.586 |
| **H-%BF** | 0.99 | 0.694 | 1.413 | 0.957 | 0.939 | 0.558 | 1.581 | 0.814 | 1.059 | 0.649 | 1.726 | 0.819 |
| **H-Insulin** | 1.844 | 1.107 | 3.074 | 0.018 | 1.267 | 0.622 | 2.582 | 0.516 | 3.126 | 1.441 | 6.778 | 0.003 |
| **H-Glucose** | 2.057 | 0.781 | 5.42 | 0.137 | 2.015 | 0.527 | 7.699 | 0.298 | 2.146 | 0.526 | 8.762 | 0.278 |
| **H-HOMA** | 1.361 | 0.821 | 2.258 | 0.232 | 1.009 | 0.496 | 2.054 | 0.981 | 2.083 | 0.982 | 4.418 | 0.052 |
| **H-Cholesterol** | 0.855 | 0.44 | 1.661 | 0.643 | 0.481 | 0.177 | 1.306 | 0.144 | 1.739 | 0.665 | 4.551 | 0.256 |
| **H-Triglycerides** | 1.097 | 0.714 | 1.683 | 0.674 | 0.846 | 0.464 | 1.541 | 0.586 | 1.56 | 0.833 | 2.922 | 0.164 |
| **L-HDL** | 1.229 | 0.858 | 1.76 | 0.26 | 1.111 | 0.62 | 1.989 | 0.725 | 1.258 | 0.781 | 2.028 | 0.347 |
| **H-LDL** | 0.95 | 0.408 | 2.212 | 0.906 | 1.026 | 0.35 | 3.008 | 0.963 | 0.897 | 0.227 | 3.544 | 0.877 |
| | | | | | | **rs12188996** | | | | | | |
| | | **Total** | | | | **Men** | | | | **Women** | | |
| | **OR** | **95% CI** | | ***p-value*** | **OR** | **95% CI** | | ***p-value*** | **OR** | **95% CI** | | ***p-value*** |

| | | | | | | | | | | | | |
|---|---|---|---|---|---|---|---|---|---|---|---|---|
| **Clinical marker** | 0.574 | 0.118 | 2.792 | 0.487 | 0.461 | 0.051 | 4.185 | 0.483 | 0.728 | 0.075 | 7.088 | 0.784 |
| **H- Waist** | 0.275 | 0.034 | 2.213 | 0.195 | 0.75 | 0.082 | 6.823 | 0.799 | 0.629 | 0.577 | 0.686 | 0.127 |
| **H-WHI** | 0.388 | 0.08 | 1.881 | 0.224 | 0.486 | 0.08 | 2.952 | 0.425 | 0.719 | 0.67 | 0.771 | 0.213 |
| **H-WHR** | 0.67 | 0.166 | 2.705 | 0.572 | 0.331 | 0.037 | 3.002 | 0.304 | 1.354 | 0.188 | 9.744 | 0.763 |
| **H-%BF** | 0.783 | 0.174 | 3.53 | 0.75 | 0.305 | 0.031 | 2.969 | 0.281 | 2.361 | 0.212 | 26.327 | 0.474 |
| **H-Insulin** | 0.739 | 0.088 | 6.229 | 0.781 | 0.764 | 0.707 | 0.826 | 0.339 | 2.089 | 0.21 | 20.746 | 0.523 |
| **H-Glucose** | 0.971 | 0.957 | 0.985 | 0.605 | 0.968 | 0.948 | 0.989 | 0.686 | 0.974 | 0.956 | 0.992 | 0.744 |
| **H-HOMA** | 0.767 | 0.095 | 6.218 | 0.803 | 1.159 | 0.126 | 10.623 | 0.897 | 0.892 | 0.857 | 0.929 | 0.489 |
| **H-Cholesterol** | 1.503 | 0.184 | 12.284 | 0.703 | 0.9 | 0.866 | 0.936 | 0.459 | 5.647 | 0.557 | 57.201 | 0.1 |
| **H-Triglycerides** | 1.133 | 0.232 | 5.526 | 0.877 | 0.754 | 0.083 | 6.855 | 0.802 | 1.785 | 0.182 | 17.52 | 0.616 |
| **L-HDL** | 0.889 | 0.22 | 3.59 | 0.861 | 0.739 | 0.081 | 6.725 | 0.789 | 1.179 | 0.164 | 8.475 | 0.871 |
| **H-LDL** | 2.692 | 0.325 | 22.334 | 0.341 | 0.94 | 0.912 | 0.968 | 0.572 | 10.963 | 1.037 | 115.915 | 0.013 |
| | | | | | | | **rs60258111** | | | | | |
| | | **Total** | | | | | **Men** | | | | **Women** | |
| **Clinical marker** | **OR** | **95% CI** | | ***p*-value** | **OR** | **95% CI** | | ***p*-value** | **OR** | **95% CI** | | ***p*-value** |
| **H-BMI** | 1.222 | 0.289 | 5.169 | 0.785 | 5.747 | 0.59 | 56.007 | 0.09 | 0.684 | 0.633 | 0.739 | 0.176 |
| **H- Waist** | 1.358 | 0.321 | 5.746 | 0.677 | 3.072 | 0.425 | 22.224 | 0.244 | 0.584 | 0.06 | 5.684 | 0.641 |
| **H-WHI** | 0.811 | 0.192 | 3.427 | 0.776 | 2.204 | 0.226 | 21.448 | 0.487 | 0.717 | 0.667 | 0.77 | 0.211 |
| **H-WHR** | 1.336 | 0.331 | 5.395 | 0.684 | 4.059 | 0.417 | 39.505 | 0.194 | 0.44 | 0.045 | 4.28 | 0.469 |
| **H-%BF** | 0.841 | 0.224 | 3.166 | 0.799 | 2.786 | 0.286 | 27.119 | 0.359 | 0.296 | 0.033 | 2.679 | 0.252 |
| **H-Insulin** | 1.478 | 0.152 | 14.405 | 0.736 | 1.656 | 0.147 | 18.683 | 0.682 | 0.858 | 0.813 | 0.906 | 0.686 |
| **H-Glucose** | 0.971 | 0.957 | 0.985 | 0.604 | 0.968 | 0.948 | 0.989 | 0.713 | 0.974 | 0.956 | 0.992 | 0.718 |
| **H-HOMA** | 0.857 | 0.828 | 0.888 | 0.233 | 0.819 | 0.773 | 0.868 | 0.35 | 0.891 | 0.855 | 0.928 | 0.436 |
| **H-Cholesterol** | 3.422 | 0.691 | 16.961 | 0.11 | 3.024 | 0.304 | 30.057 | 0.323 | 4.191 | 0.444 | 39.577 | 0.176 |
| **H-Triglycerides** | 0.478 | 0.059 | 3.861 | 0.48 | 0.991 | 0.101 | 9.676 | 0.994 | 0.834 | 0.794 | 0.877 | 0.322 |
| **L-HDL** | 0.89 | 0.22 | 3.597 | 0.871 | 0.745 | 0.695 | 0.797 | 0.244 | 1.783 | 0.294 | 10.822 | 0.526 |
| **H-LDL** | 2.683 | 0.323 | 22.254 | 0.342 | 0.94 | 0.912 | 0.968 | 0.614 | 8.139 | 0.825 | 80.33 | 0.034 |
| | | | | | | | **rs16878426** | | | | | |
| | | **Total** | | | | | **Men** | | | | **Women** | |

| Clinical marker | OR | 95% CI | | *p-value* | OR | 95% CI | | *p-value* | OR | 95% CI | | *p-value* |
|---|---|---|---|---|---|---|---|---|---|---|---|---|
| **H-BMI** | 0.661 | 0.624 | 0.701 | 0.018 | 0.641 | 0.587 | 0.7 | 0.069 | 0.68 | 0.629 | 0.735 | 0.127 |
| **H- Waist** | 0.219 | 0.028 | 1.72 | 0.113 | 0.746 | 0.697 | 0.799 | 0.156 | 0.426 | 0.047 | 3.862 | 0.437 |
| **H-WHI** | 0.777 | 0.225 | 2.685 | 0.69 | 0.728 | 0.144 | 3.673 | 0.701 | 0.641 | 0.071 | 5.819 | 0.692 |
| **H-WHR** | 0.563 | 0.51 | 0.622 | 0.004 | 0.559 | 0.504 | 0.621 | 0.031 | 0.563 | 0.51 | 0.622 | 0.051 |
| **H-%BF** | 0.382 | 0.1 | 1.454 | 0.144 | 0.447 | 0.081 | 2.483 | 0.347 | 0.287 | 0.032 | 2.599 | 0.239 |
| **H-Insulin** | 0.554 | 0.068 | 4.498 | 0.576 | 0.762 | 0.704 | 0.824 | 0.215 | 2.111 | 0.213 | 20.966 | 0.516 |
| **H-Glucose** | 0.971 | 0.958 | 0.985 | 0.568 | 0.968 | 0.948 | 0.989 | 0.657 | 0.974 | 0.956 | 0.992 | 0.715 |
| **H-HOMA** | 0.772 | 0.095 | 6.258 | 0.808 | 0.819 | 0.773 | 0.868 | 0.297 | 2.878 | 0.29 | 28.547 | |
| **H-Cholesterol** | 0.92 | 0.898 | 0.942 | 0.329 | 0.897 | 0.862 | 0.933 | 0.408 | 0.941 | 0.915 | 0.968 | 0.347 |
| **H-Triglycerides** | 0.865 | 0.184 | 4.055 | 0.854 | 0.744 | 0.694 | 0.797 | 0.153 | 3.583 | 0.583 | 22.017 | 0.143 |
| **L-HDL** | 1.011 | 0.293 | 3.493 | 0.987 | 0.744 | 0.694 | 0.797 | 0.153 | 4.743 | 0.524 | 42.925 | 0.128 |
| **H-LDL** | 0.954 | 0.937 | 0.971 | 0.467 | 0.94 | 0.912 | 0.968 | 0.536 | 0.967 | 0.948 | 0.987 | 0.682 |
| | | | | | | **rs6872863** | | | | | | |
| | | **Total** | | | | **Men** | | | | **Women** | | |
| **Clinical marker** | **OR** | **95% CI** | | ***p-value*** | **OR** | **95% CI** | | ***p-value*** | **OR** | **95% CI** | | ***p-value*** |
| **H-BMI** | 1.212 | 0.839 | 1.75 | 0.306 | 1.115 | 0.661 | 1.881 | 0.685 | 1.3 | 0.775 | 2.18 | 0.321 |
| **H- Waist** | 1.068 | 0.74 | 1.541 | 0.726 | 1.114 | 0.625 | 1.983 | 0.716 | 1.086 | 0.67 | 1.761 | 0.739 |
| **H-WHI** | 1.094 | 0.777 | 1.541 | 0.606 | 1.414 | 0.86 | 2.324 | 0.173 | 0.748 | 0.449 | 1.246 | 0.265 |
| **H-WHR** | 1.226 | 0.868 | 1.731 | 0.247 | 1.171 | 0.709 | 1.932 | 0.539 | 1.279 | 0.794 | 2.06 | 0.313 |
| **H-%BF** | 1.302 | 0.922 | 1.839 | 0.134 | 1.387 | 0.839 | 2.291 | 0.203 | 1.209 | 0.751 | 1.948 | 0.436 |
| **H-Insulin** | 0.886 | 0.53 | 1.484 | 0.647 | 0.806 | 0.401 | 1.62 | 0.547 | 0.908 | 0.416 | 1.982 | 0.81 |
| **H-Glucose** | 1.19 | 0.408 | 3.473 | 0.75 | 0.595 | 0.156 | 2.27 | 0.445 | 3.638 | 0.432 | 30.608 | 0.205 |
| **H-HOMA** | 1.114 | 0.666 | 1.864 | 0.682 | 1.231 | 0.608 | 2.492 | 0.565 | 0.927 | 0.431 | 1.992 | 0.846 |
| **H-Cholesterol** | 0.825 | 0.445 | 1.53 | 0.543 | 0.912 | 0.406 | 2.047 | 0.824 | 0.651 | 0.244 | 1.737 | 0.389 |
| **H-Triglycerides** | 0.725 | 0.482 | 1.09 | 0.121 | 0.638 | 0.367 | 1.11 | 0.111 | 0.787 | 0.425 | 1.457 | 0.446 |
| **L-HDL** | 0.957 | 0.675 | 1.357 | 0.805 | 0.749 | 0.429 | 1.309 | 0.311 | 1.222 | 0.768 | 1.943 | 0.398 |
| **H-LDL** | 0.483 | 0.223 | 1.048 | 0.061 | 0.406 | 0.151 | 1.089 | 0.066 | 0.582 | 0.165 | 2.055 | 0.397 |
| | | | | | | **rs76641655** | | | | | | |

| | Total | | | | Men | | | | Women | | | |
|---|---|---|---|---|---|---|---|---|---|---|---|---|
| Clinical marker | OR | 95% CI | | *p-value* | OR | 95% CI | | *p-value* | OR | 95% CI | | *p-value* |
| **H-BMI** | 1.008 | 0.373 | 2.728 | 0.988 | 0.927 | 0.227 | 3.79 | 0.916 | 1.092 | 0.267 | 4.464 | 0.902 |
| **H- Waist** | 0.63 | 0.205 | 1.941 | 0.418 | 0.371 | 0.046 | 3.022 | 0.337 | 0.859 | 0.211 | 3.504 | 0.833 |
| **H-WHI** | 0.514 | 0.181 | 1.46 | 0.204 | 0.908 | 0.239 | 3.456 | 0.888 | 0.712 | 0.663 | 0.766 | 0.058 |
| **H-WHR** | 0.84 | 0.321 | 2.198 | 0.722 | 0.654 | 0.16 | 2.669 | 0.553 | 1.065 | 0.28 | 4.044 | 0.927 |
| **H-%BF** | 0.826 | 0.321 | 2.125 | 0.693 | 0.726 | 0.191 | 2.762 | 0.638 | 0.933 | 0.246 | 3.547 | 0.92 |
| **H-Insulin** | 0.29 | 0.038 | 2.232 | 0.207 | 0.403 | 0.049 | 3.308 | 0.385 | 0.857 | 0.812 | 0.905 | 0.283 |
| **H-Glucose** | 2.077 | 0.26 | 16.579 | 0.482 | 0.968 | 0.947 | 0.989 | 0.586 | 5.268 | 0.578 | 48.013 | 0.101 |
| **H-HOMA** | 0.38 | 0.05 | 2.908 | 0.334 | 0.574 | 0.07 | 4.706 | 0.602 | 0.891 | 0.856 | 0.928 | 0.326 |
| **H-Cholesterol** | 0.684 | 0.089 | 5.257 | 0.714 | 1.121 | 0.135 | 9.291 | 0.916 | 0.94 | 0.914 | 0.967 | 0.452 |
| **H-Triglycerides** | 2.579 | 0.978 | 6.798 | 0.048 | 1.522 | 0.371 | 6.248 | 0.559 | 4.452 | 1.152 | 17.204 | 0.019 |
| **L-HDL** | 1.435 | 0.558 | 3.691 | 0.453 | 1.522 | 0.371 | 6.248 | 0.559 | 1.466 | 0.386 | 5.565 | 0.574 |
| **H-LDL** | 0.954 | 0.937 | 0.971 | 0.35 | 0.939 | 0.911 | 0.968 | 0.447 | 0.967 | 0.947 | 0.987 | 0.58 |

**rs114011218**

| | Total | | | | Men | | | | Women | | | |
|---|---|---|---|---|---|---|---|---|---|---|---|---|
| Clinical marker | OR | 95% CI | | *p-value* | OR | 95% CI | | *p-value* | OR | 95% CI | | *p-value* |
| **H-BMI** | 0.179 | 0.023 | 1.393 | 0.065 | 0.641 | 0.587 | 0.7 | 0.069 | 0.43 | 0.05 | 3.73 | 0.432 |
| **H- Waist** | 0.663 | 0.18 | 2.438 | 0.534 | 1.529 | 0.274 | 8.53 | 0.627 | 0.281 | 0.033 | 2.366 | 0.215 |
| **H-WHI** | 0.403 | 0.11 | 1.479 | 0.157 | 0.728 | 0.144 | 3.673 | 0.701 | 0.714 | 0.665 | 0.767 | 0.096 |
| **H-WHR** | 0.823 | 0.266 | 2.547 | 0.736 | 0.653 | 0.118 | 3.624 | 0.625 | 0.996 | 0.219 | 4.529 | 0.996 |
| **H-%BF** | 0.686 | 0.192 | 2.457 | 0.561 | 0.601 | 0.099 | 3.654 | 0.578 | 0.776 | 0.128 | 4.714 | 0.783 |
| **H-Insulin** | 0.563 | 0.069 | 4.572 | 0.587 | 0.768 | 0.711 | 0.83 | 0.275 | 1.575 | 0.17 | 14.573 | 0.688 |
| **H-Glucose** | 0.971 | 0.957 | 0.985 | 0.534 | 0.968 | 0.948 | 0.989 | 0.657 | 0.974 | 0.956 | 0.992 | 0.665 |
| **H-HOMA** | 0.566 | 0.072 | 4.451 | 0.585 | 1.198 | 0.131 | 10.981 | 0.874 | 0.892 | 0.856 | 0.928 | 0.359 |
| **H-Cholesterol** | 0.976 | 0.124 | 7.677 | 0.982 | 0.897 | 0.862 | 0.933 | 0.408 | 2.814 | 0.32 | 24.712 | 0.332 |
| **H-Triglycerides** | 1.175 | 0.318 | 4.336 | 0.809 | 0.592 | 0.068 | 5.149 | 0.632 | 2.133 | 0.402 | 11.316 | 0.364 |
| **L-HDL** | 1.116 | 0.361 | 3.455 | 0.849 | 0.747 | 0.698 | 0.8 | 0.157 | 2.968 | 0.567 | 15.534 | 0.179 |
| **H-LDL** | 1.792 | 0.224 | 14.306 | 0.578 | 0.94 | 0.912 | 0.968 | 0.536 | 5.463 | 0.594 | 50.222 | 0.094 |

| | | | | | | | | | | | | |
|---|---|---|---|---|---|---|---|---|---|---|---|---|
| | | | | | | rs115159664 | | | | | | |
| | Total | | | | Men | | | | Women | | | |
| **Clinical marker** | **OR** | **95% CI** | | ***p-value*** | **OR** | **95% CI** | | ***p-value*** | **OR** | **95% CI** | | ***p-value*** |
| **H-BMI** | 0.62 | 0.224 | 1.718 | 0.355 | 0.454 | 0.094 | 2.179 | 0.314 | 0.811 | 0.21 | 3.128 | 0.762 |
| **H- Waist** | 0.89 | 0.34 | 2.331 | 0.813 | 0.329 | 0.041 | 2.64 | 0.273 | 1.458 | 0.435 | 4.891 | 0.541 |
| **H-WHI** | 0.832 | 0.34 | 2.039 | 0.688 | 0.473 | 0.131 | 1.715 | 0.246 | 1.498 | 0.427 | 5.253 | 0.527 |
| **H-WHR** | 0.654 | 0.26 | 1.644 | 0.364 | 0.32 | 0.067 | 1.536 | 0.136 | 1.111 | 0.332 | 3.723 | 0.865 |
| **H-%BF** | 0.684 | 0.275 | 1.699 | 0.411 | 0.108 | 0.013 | 0.879 | 0.012 | 2.103 | 0.602 | 7.343 | 0.236 |
| **H-Insulin** | 1.235 | 0.336 | 4.536 | 0.751 | 0.463 | 0.056 | 3.868 | 0.47 | 3.259 | 0.571 | 18.599 | 0.162 |
| **H-Glucose** | 1.756 | 0.222 | 13.903 | 0.59 | 0.968 | 0.947 | 0.989 | 0.565 | 4.186 | 0.469 | 37.327 | 0.165 |
| **H-HOMA** | 0.644 | 0.147 | 2.824 | 0.558 | 0.816 | 0.769 | 0.866 | 0.136 | 1.939 | 0.399 | 9.409 | 0.405 |
| **H-Cholesterol** | 2.927 | 0.943 | 9.086 | 0.052 | 0.992 | 0.121 | 8.122 | 0.994 | 7.125 | 1.714 | 29.621 | 0.002 |
| **H-Triglycerides** | 0.402 | 0.092 | 1.749 | 0.21 | 0.741 | 0.691 | 0.794 | 0.063 | 1.167 | 0.244 | 5.568 | 0.847 |
| **L-HDL** | 0.701 | 0.268 | 1.834 | 0.468 | 2.059 | 0.564 | 7.512 | 0.266 | 0.247 | 0.053 | 1.164 | 0.057 |
| **H-LDL** | 3.847 | 1.06 | 13.959 | 0.028 | 1.819 | 0.217 | 15.261 | 0.577 | 8.111 | 1.503 | 43.76 | 0.004 |
| | | | | | | rs4700398 | | | | | | |
| | Total | | | | Men | | | | Women | | | |
| **Clinical marker** | **OR** | **95% CI** | | ***p-value*** | **OR** | **95% CI** | | ***p-value*** | **OR** | **95% CI** | | ***p-value*** |
| **H-BMI** | 1.57 | 1.109 | 2.221 | 0.011 | 1.272 | 0.779 | 2.078 | 0.337 | 1.964 | 1.196 | 3.225 | 0.007 |
| **H- Waist** | 1.299 | 0.916 | 1.841 | 0.142 | 1.241 | 0.725 | 2.124 | 0.433 | 1.315 | 0.825 | 2.094 | 0.25 |
| **H-WHI** | 0.985 | 0.71 | 1.365 | 0.926 | 1.153 | 0.72 | 1.848 | 0.555 | 0.895 | 0.543 | 1.473 | 0.663 |
| **H-WHR** | 1.438 | 1.036 | 1.996 | 0.03 | 1.358 | 0.847 | 2.177 | 0.204 | 1.517 | 0.96 | 2.396 | 0.074 |
| **H-%BF** | 1.473 | 1.058 | 2.051 | 0.022 | 1.441 | 0.894 | 2.323 | 0.134 | 1.522 | 0.959 | 2.415 | 0.074 |
| **H-Insulin** | 1.06 | 0.645 | 1.742 | 0.819 | 0.898 | 0.461 | 1.749 | 0.754 | 1.32 | 0.616 | 2.828 | 0.477 |
| **H-Glucose** | 0.75 | 0.282 | 1.998 | 0.565 | 0.557 | 0.137 | 2.273 | 0.411 | 1.034 | 0.254 | 4.21 | 0.963 |
| **H-HOMA** | 1.2 | 0.739 | 1.95 | 0.462 | 0.982 | 0.515 | 1.874 | 0.957 | 1.658 | 0.774 | 3.554 | 0.192 |
| **H-Cholesterol** | 1.087 | 0.596 | 1.985 | 0.785 | 1.242 | 0.576 | 2.68 | 0.589 | 0.914 | 0.343 | 2.433 | 0.857 |
| **H-Triglycerides** | 1.035 | 0.694 | 1.544 | 0.865 | 1.228 | 0.718 | 2.101 | 0.455 | 0.858 | 0.468 | 1.575 | 0.623 |
| **L-HDL** | 1.004 | 0.718 | 1.404 | 0.981 | 0.781 | 0.454 | 1.342 | 0.372 | 1.148 | 0.734 | 1.797 | 0.546 |

| | | | | | | | | | | | | |
|---|---|---|---|---|---|---|---|---|---|---|---|---|
| **H-LDL** | 1.003 | 0.463 | 2.173 | 0.993 | 1.009 | 0.378 | 2.693 | 0.986 | 1.034 | 0.293 | 3.646 | 0.959 |

H-BMI: High Body Mass Index >25.0 kg/m2; H-WC: High Waist circumference (Women >0.80 cm and Men >90 cm); H-WHI: High Waist-Hip Index (Women >0.85 cm and Men >95 cm); H-WHR: High Waist-Height Ratio >0.50; H-%BF: High Body Fat percent (Women >35% and Men >20%). H-Glucose: High glucose; > 100 mg/dL; H-INS: High Insulin (>14 μU/ml for women and> 11μU/ml for men); H-HOMA: High HOMA index (>2.9 for women and >2.3 for men); H-Cholesterol: High total cholesterol (>200 mg/dL); H-LDL: Elevated low-density lipoproteins (>130 mg/dL); L-HDL: Low high-density lipoproteins (≤50 mg/dL for women and ≤40 mg/dL for men); H-Triglycerides : High triglycerides (>150 mg/dL). The statistical analysis applied to this dataset was a multinomial regression (p=<0.05).
